# Multi-omics provide insights into the regulation of DNA methylation in pear fruit metabolism

**DOI:** 10.1186/s13059-024-03200-2

**Published:** 2024-03-14

**Authors:** Chao Gu, Mao-Song Pei, Zhi-Hua Guo, Lei Wu, Kai-Jie Qi, Xue-Ping Wang, Hong Liu, Zhongchi Liu, Zhaobo Lang, Shaoling Zhang

**Affiliations:** 1 Jiangsu Engineering Research Center for Pear, State Key Laboratory of Crop Genetics and Germplasm Enhancement, Nanjing Agricultural University, Nanjing, 210095 China; 2 Department of Cell Biology and Molecular Genetics, University of Maryland, College Park, MD 20742 USA; 3 Institute of Advanced Biotechnology and School of Life Sciences, Southern University of Science and Technology, Shenzhen, 518055 China; 4 Shanghai Center for Plant Stress Biology, National Key Laboratory of Plant Molecular Genetics, Center of Excellence in Molecular Plant Sciences, Shanghai Institutes for Biological Sciences, Chinese Academy of Sciences, Shanghai, 200032 China

**Keywords:** *Pyrus bretschneideri*, Flesh development, Fruit metabolism, DNA methylation, Multi-omics

## Abstract

**Background:**

Extensive research has been conducted on fruit development in crops, but the metabolic regulatory networks underlying perennial fruit trees remain poorly understood. To address this knowledge gap, we conduct a comprehensive analysis of the metabolome, proteome, transcriptome, DNA methylome, and small RNAome profiles of pear fruit flesh at 11 developing stages, spanning from fruitlet to ripening. Here, we systematically investigate the metabolic landscape and regulatory network involved.

**Results:**

We generate an association database consisting of 439 metabolites and 14,399 genes to elucidate the gene regulatory network of pear flesh metabolism. Interestingly, we detect increased DNA methylation in the promoters of most genes within the database during pear flesh development. Application of a DNA methylation inhibitor to the developing fruit represses chlorophyll degradation in the pericarp and promotes xanthophyll, β-carotene, and abscisic acid (ABA) accumulation in the flesh. We find the gradual increase in ABA production during pear flesh development is correlated with the expression of several carotenoid pathway genes and multiple transcription factors. Of these transcription factors, the zinc finger protein PbZFP1 is identified as a positive mediator of ABA biosynthesis in pear flesh. Most ABA pathway genes and transcription factors are modified by DNA methylation in the promoters, although some are induced by the DNA methylation inhibitor. These results suggest that DNA methylation inhibits ABA accumulation, which may delay fruit ripening.

**Conclusion:**

Our findings provide insights into epigenetic regulation of metabolic regulatory networks during pear flesh development, particularly with regard to DNA methylation.

**Supplementary Information:**

The online version contains supplementary material available at 10.1186/s13059-024-03200-2.

## Background

Fruit development is a distinctive process in the life cycle of higher plants, contributing to the production of metabolites that provide humans with a diet rich in health-promoting compounds. As the metabolites accumulate, a series of physiological changes accompany fruit expansion and ripening. At present, a total of 7118 metabolic peaks were identified in tomato fruit, but only 22% of which have been annotated [1]. Among the annotated metabolites, 362 can be easily detected using the broadly targeted metabolic profiling method [2]. Similarly, more than 2000 metabolite peaks were detected in peach and orange fruits [3, 4], but only 493 metabolites have been annotated [5]. The annotated metabolites in tomatoes are classified into 16 categories, while peaches have 33 classes [1, 5]. Among these categories or classes, sugar, organic acid, flavonoids, fatty acid, polyphenols, terpene, and phytohormone have been extensively studied in various fleshy fruits [6–8]. Although the biosynthesis pathways of the annotated metabolites are similar across different species, the spatio-temporal distribution and natural variation of the metabolites vary widely among different species and even different cultivars within the same species [4].

Metabolic pathways are catalyzed by a series of enzymes that can be partially identified through proteomic sequencing in fleshy fruits [9, 10]. Unfortunately, the investigation of regulatory networks governing fruit metabolic pathways based solely on protein abundance is challenging [11–16]. In comparison, transcriptome analysis is more commonly employed to explore metabolic pathways in fruits. In tomato fruit, the integration of transcriptome and metabolome data has revealed the regulatory networks governing polyphenolic, steroidal glycoalkaloid, and flavonoid metabolisms [17, 18]. Additionally, a gene-metabolite network activated by the SlMYB12 transcription factor (TF) governs the conversion of pink to red pigments [2]. Moreover, the integrative analyses have unveiled a regulatory network governing flavor metabolism in kiwifruit [14].

Transcriptional activity is influenced by chromatin compaction that is regulated by DNA methylation and post-translational modifications of histones (PTMH). Comparing to PTMH, DNA methylation, which has been more extensively investigated in fleshy fruits due to its involvement in anthocyanin accumulation [19, 20], flavor biosynthesis [21, 22], and fruit development and ripening [23–26]. Plant DNA methylation occurs in two symmetrical contexts, CG and CHG, as well as an asymmetrical context, CHH (H represents A, T, or C). In *Arabidopsis*, CG, CHG, and CHH methylations are maintained by METHYLTRANSFERASE 1 (MET1), CHROMOMETHYLASE 3 (CMT3), and CMT2 respectively [27–29]. Moreover, all three contexts are established by DOMAINS REARRANGED METHYLASEs (DRMs) via the RNA-directed DNA methylation (RdDM) pathway. In RdDM, double-stranded RNAs are generated through RNA POLYMERASE IV (POL IV) transcription and RNA-DEPENDENT RNA POLYMERASE 2 (RDR2)-dependent transcript copying, then cleaved by DICER-LIKE PROTEIN 3 (DCL3) to produce 24-nt small interfering RNAs (siRNAs) [27, 30, 31]. Subsequently, the siRNAs are loaded onto ARGONAUTE 4 (AGO4) and AGO6, and they pair with nascent scaffold RNAs produced by POL V to induce cytosine methylation. DNA methylation at all three contexts can be excised by 5’-methylcytosine DNA glycosylase/lyase enzymes, including REPRESSOR OF SILENCING 1 (ROS 1), TRANSCRIPTIONAL ACTIVATOR DEMETER (DME), DEMETER-LIKE PROTEIN 2 (DML2), and DML3 [27, 32, 33]. Regions targeted by ROS1 lack H3K27me and H3K9me2, but are enriched with H3K27me3 and acetylated H3K18 [27, 34]. The chromatin modification of ROS1 targets is regulated by a histone acetyltransferase INCREASED DNA METHYLATION (IDM1) [27, 35]. While mechanisms of DNA methylation and demethylation have been clearly elucidated in *Arabidopsis* and a few fleshy fruits [25–27], the regulatory mechanisms of DNA methylation during fruit development remain poorly understood.

Pear is a globally cultivated fruit crops and extensive research has been conducted to investigate pear DNA methylation, using four methods: methylation-sensitive amplification polymorphism [36], methylation-specific endonuclease-PCR [19, 37, 38], TLC-based detection [39], and genome-wide bisulfite sequencing [40]. It is determined that DNA methylation is increased in colchicine-induced polyploidy [36] and in long-term stored and severely desiccated seeds [39]. Inhibition of DNA methylation using the DNA methylation inhibitor 5-azacytidine (5’-Aza) decreases ethylene production in suspension-cultured pear fruit cells [41]. Moreover, cytosine methylation in the promoters of *MYB10* and *GA2ox8* influences anthocyanin accumulation in fruit skin [19, 37, 38]. However, little is known about the impact of DNA methylation on pear fruit metabolism. In this study, the metabolome, transcriptome, proteome, DNA methylome, and small RNAome were profiled at 11 stages of pear fruit flesh development, ranging from fruitlet to ripening. Integrative analyses of these omics datasets revealed a global increase in DNA methylation and abscisic acid (ABA) content during pear fruit flesh development. Application of 5’-Aza resulted in increased expression of ABA biosynthetic genes and their upstream TFs, leading to a significant accumulation of ABA in the fruit flesh. This novel information sheds light on the regulatory role of DNA methylation in fruit flesh metabolite production in pear.

## Results

### Metabolic profiling during pear flesh development

**Fig. 1:**
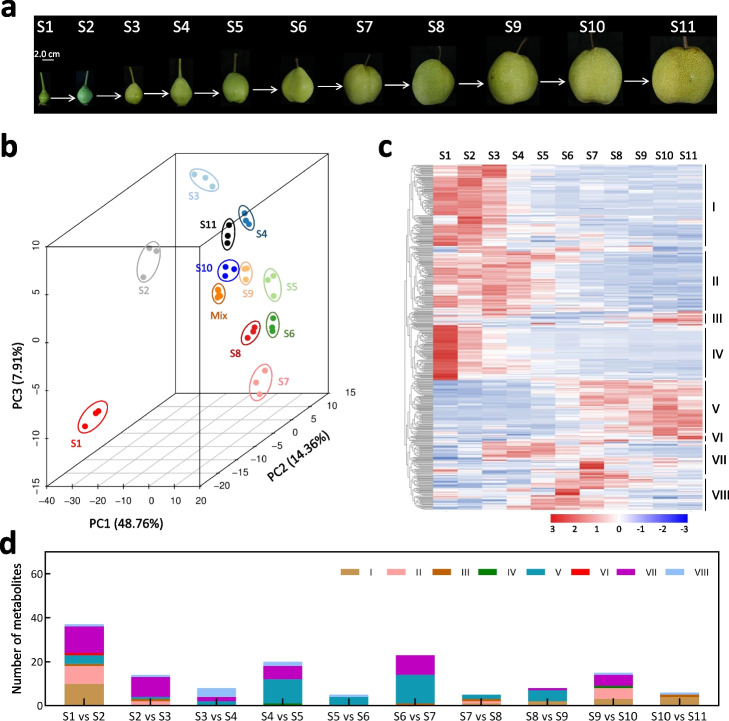
Dynamics of metabolite during pear flesh development. **a** The pear fruits collected from 11 stages. **b** Principal component analysis of metabolome data in the fruit flesh at 11 stages. **c** Clustering analysis of metabolic profiling grouped the differentially accumulated metabolites into eight clusters, I → VIII. *Z*-score standardized values of each metabolite across all 11 stages were used for clustering analysis. The color bar denotes an increase in the contents of each metabolite, transitioning from blue to red color. **d** The number and cluster of the metabolites that were significantly increased in the next stage. S1 to S11 indicate the pear fruit at 4, 6, 8, 10, 12, 14, 16, 18, 20, 22, and 24 weeks after flower blooming, respectively

To survey the changes in metabolites during pear flesh development, cv. Dangshansuli fruits were firstly collected at different time points, specifically at 4 (S1), 6 (S2), 8 (S3), 10 (S4), 12 (S5), 14 (S6), 16 (S7), 18 (S8), 20 (S9), 22 (S10), and 24 (S11) weeks after flower blooming (Fig. 1a). The typical characteristics of fruit development were observed during this period (Additional file 1: Fig. S1). These included a gradual increase in single fruit weight, longitudinal and transverse diameters, and soluble sugar content. Ethylene content showed a substantial increase at S10 and S11. Stone cell content increased from S1 to S3, but then gradually decreased. Flesh firmness decreased gradually from S8 to S11, while soluble solids increased gradually from S4 to S11. Secondly, metabolic measurements identified a total of 492 metabolites in pear flesh. On average, approximately 456 metabolites were detected at each stage, with 371 metabolites being commonly present across all stages (Additional file 2: Table S1). Principal component analysis confirmed distinct differences among the fruit flesh at the 11 stages (Fig. 1b). By comparing the relative content between each pair of stages (Additional file 2: Table S2), a total of 449 differentially accumulated metabolites (DAMs) were identified. These DAMs belonged to 32 different classes, including phytohormones, anthocyanins, amino and their derivatives, carbohydrates, flavone, flavonol, hydroxycinnamoyl derivatives, lipids, nucleotide and its derivatives, and organic acids, and presented eight clusters (I → VIII) based on their accumulation trends (Fig. 1c, Additional file 2: Table S3). Clusters I, II, and IV showed higher levels of metabolites in the fruit flesh at the early stages compared to later stages (Additional file 1: Fig. S2), indicating their association with early flesh development. An example is the sinapyl alcohol in cluster II, which was associated with the lignification of stone cells that are enriched in pear flesh from 15 to 55 days after flowering [42]. Clusters VII and VIII exhibited higher levels of metabolites in the fruit flesh at S7 and S6, respectively (Additional file 1: Fig. S2), indicating that these metabolites, including L-Alanine, L-Phenylalanine, L-Proline, and L-Valine in cluster VII, may be associated with fruit enlargement [43, 44]. It is reported that amino acids are significantly accumulated during pear fruit enlargement [45]. Clusters III, V, and VI showed higher levels of metabolites in the flesh at S10 and S11 compared to other stages (Additional file 1: Fig. S2), indicating their association with fruit ripening. For example, abscisic acid (ABA) in cluster V can increase the content of soluble sugars (including sucrose and glucose) and promote ethylene release, thereby promoting pear fruit ripening [46].

To mine the metabolites that promote flesh development, we further analyzed the DAMs between two adjacent stages (Additional file 1: Fig. S3a) and found that transition from one type of metabolite to another was more frequent in the early stages of flesh development compared to the later stages (Additional file 1: Fig. S3b). A total of 105 metabolites belonging to 27 classes were significantly accumulated in the flesh at the next stage compared to the previous stage, suggesting their potential importance in pear flesh development. Interestingly, the metabolites between any two adjacent stages belonged to at least two clusters (Fig. 1d). Moreover, the transition from S1 to S2 and from S2 to S3 involved 14 and 11 metabolite classes, respectively, while the transition from S9 to S10 and from S10 to S11 involved seven and four metabolite classes, respectively (Table 1). This result indicates that early flesh development is more dependent on the metabolite biosynthesis compared to fruit ripening. During fruit enlargement (from S3 to S9) [47], the transition from S4 to S5 and from S6 to S7 involved 13 and 12 metabolite classes, respectively, while the transitions from S3 to S4, from S5 to S6, from S7 to S8, and from S8 to S9 involved seven, five, five, and eight metabolite classes, respectively (Table 1). These results indicate that the biosynthesis of metabolites from S4 to S5 and from S6 to S7 may play important roles in fruit enlargement.

**Table 1:** The classes of metabolites involved in the transition from one stage to the next

Metabolite class	S1 vs S2	S2 vs S3	S3 vs S4	S4 vs S5	S5 vs S6	S6 vs S7	S7 vs S8	S8 vs S9	S9 vs S10	S10 vs S11
Alcohols and polyols	0	0	0	0	1	0	0	0	0	0
Alkaloids	1	0	0	0	0	0	1	0	1	0
Amino acid derivatives	3	0	0	3	0	0	1	0	3	0
Amino acid	7	0	0	0	1	0	0	1	6	2
Anthocyanins	0	1	0	0	0	0	0	0	0	1
Benzoic acid derivatives	0	1	0	0	0	0	0	0	0	0
Carbohydrates	0	0	1	1	1	1	0	1	0	0
Catechin derivatives	0	0	0	0	0	2	1	1	0	0
Coumarins	0	0	0	1	1	2	0	1	0	0
Flavanone	0	0	0	1	0	0	0	0	0	0
Flavone	4	2	1	3	0	0	0	1	1	0
Flavone C-glycosides	3	1	0	1	0	0	1	0	0	0
Flavonol	3	1	1	0	0	0	0	1	0	0
Hydroxycinnamoyl derivatives	3	3	0	1	0	3	0	0	0	0
Indole derivatives	2	0	0	0	0	0	1	1	0	2
Lipids_Fatty acids	0	0	0	0	0	1	0	1	0	0
Lipids_Glycerolipids	2	0	0	0	1	4	0	0	1	0
Lipids_Glycerophospholipids	2	0	0	0	0	0	0	0	0	1
Nucleotide and its derivates	2	1	1	2	0	1	0	0	1	0
Organic acids	2	1	1	1	0	4	0	0	0	0
Others	1	1	2	2	0	1	0	0	0	0
Phenolamides	0	0	1	2	0	0	0	0	0	0
Phytohormones	0	0	0	1	0	0	0	0	0	0
Proanthocyanidins	0	0	0	0	0	2	0	0	2	0
Quinate and its derivatives	2	1	0	0	0	0	0	0	0	0
Quinate and its derivatives	0	0	0	1	0	1	0	0	0	0
Vitamins	0	1	0	0	0	1	0	0	0	0

### Gene regulation of pear flesh metabolism

**Fig. 2:**
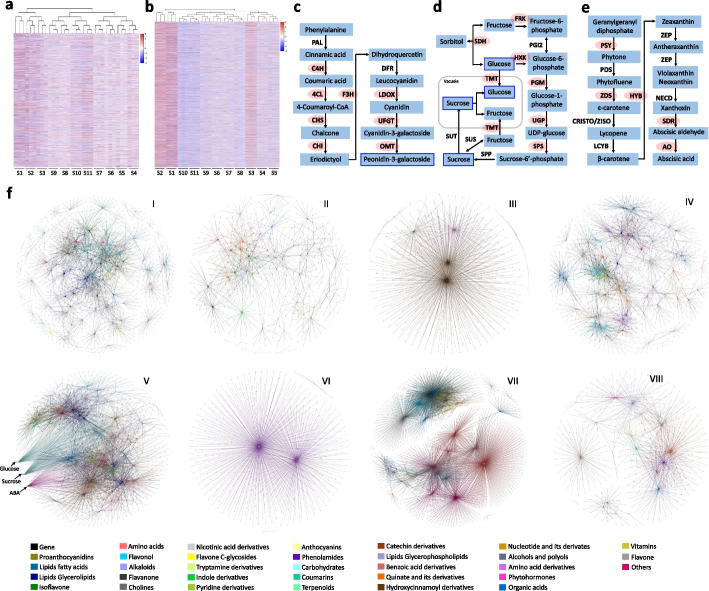
Integrative analysis of metabolome, proteome, and transcriptome data of pear flesh. **a** Clustering analysis of proteome data shows the three reliable replicates of each sample at any stage. *Z*-score standardized value of each protein across all 11 stages (from S1 to S11) were used for clustering analysis. **b** Clustering analysis of transcriptome data shows the three reliable replicates of each sample at any stage. *Z*-score standardized value of each gene across all 11 stages was used for clustering analysis. The color bar indicates the increasing expression levels of both protein and gene from blue to red. **c** Peonidin-3-galactoside biosynthesis pathway. **d** Sugar biosynthesis and metabolic process. **e** Abscisic acid biosynthesis pathway. The pathway genes with the background of red color were positively correlated to the target metabolites (highlighted by blue box). **f** The metabolic regulatory network for each metabolite cluster. I → VIII indicate the different clusters

To establish a relationship between gene activity and pear flesh metabolism, mass spectrometry was conducted to investigate the proteomes at each stage. A total of 5106 proteins were detected in all samples combined. On average, approximately 4074 proteins were detected at each stage, with 2860 proteins being consistently present across all stages (Additional file 2: Table S1). By comparing protein abundance between stages, a total of 3469 differentially expressed proteins (DEPs) were identified in the fruit flesh (Fig. 2a). Integrative analysis of the proteome and metabolome showed that 1038, 1012, 167, 843, 927, 287, 426, and 162 DEPs were positively (Pearson coefficient > 0.85 and false discovery rate < 0.05) were positively correlated with metabolites in clusters I → VIII, respectively, while 834, 883, 68, 509, 922, 60, 458, and 58 DEPs were negatively (Pearson coefficient <  − 0.85 and false discovery rate < 0.05) correlated with metabolites in the same clusters (Additional file 2: Fig. S4a). Overall, 2513 DEPs were correlated with 423 DAMs (Additional file 2: Table S4). Among these, 1581 DEPs were correlated with the metabolites in a single cluster, while 932 DEPs were correlated with metabolites in at least two clusters (Additional file 1: Fig. S4b).

To compensate for the limited number of proteins detected through mass spectrometry, transcriptome sequencing was conducted to investigate the expression patterns of all predicted genes in the pear fruit flesh. A total of 3.06 billion raw reads were generated from all samples (Additional file 2: Table S5), with 931.09 million clean reads successfully assembled into 33,051 genes. On average, approximately 28,748 genes were detected at each stage, and among them, 24,806 genes were consistently present across all 11 stages (Additional file 2: Table S1). By comparing the expression levels between each pair of stages, a total of 22,055 differentially expressed genes (DEGs) were identified in fruit flesh (Fig. 2b). Integrative analysis of the transcriptome and metabolome showed that 9524, 7120, 382, 6257, 2757, 505, 1818, and 433 DEGs were positively (Pearson coefficient > 0.85 and false discovery rate < 0.05) correlated with metabolites in clusters I → VIII, respectively, while 2138, 2564, 80, 1144, 5372, 202, 4050, and 481 DEGs were negatively (Pearson coefficient <  − 0.85 and false discovery rate < 0.05) correlated with metabolites in the same clusters (Additional file 2: Fig. S5a). Overall, 14,399 DEGs, including 2218 DEP-coding genes, were correlated with 439 DAMs (Additional file 2: Table S6). Among these DEGs, 9139 DEGs were correlated with metabolites in a single cluster, while 5260 DEGs were correlated with metabolites in at least two clusters (Additional file 1: Fig. S5b).

After removing the debatable genes presenting an inconsistent correlation with a metabolite in transcript and translation levels, a comprehensive gene-metabolite database was constructed (Additional file 3: Table S7). In this database, 220 metabolites could be detected in the Kyoto Encyclopedia of Genes and Genomes (KEGG) database. However, the transcriptome data of fruit flesh only included survey information for the pathway genes of only 101 metabolites. Out of these 101 metabolites, 63 positively or negatively correlated with their pathway genes (Additional file 3: Table S8). To further evaluate the effectiveness of this database, we conducted tests using previously reported metabolic pathways. For instance, peonidin-3-galactoside, a component of anthocyanin, was positively correlated with various anthocyanin biosynthetic genes, including *Cinnamate 4-hydroxylase*, *4-Coumarate:CoA ligase*, *Chalcone synthase*, *Chalcone isomerase*, *Flavonoid 3-hydroxylase*, *Leucoanthocyanidin dioxygenase*, *UDP-glucuronosyl/UDP-glucosyltransferase*, *O-methyltransferase*, and components of the MYB-bHLH-WD40 complex (Fig. 2c, Additional file 3: Table S7). Additionally, sucrose and glucose are positively correlated with sugar metabolism-related genes including *Sorbitol dehydrogenase*, *Fructokinase*, *Hexokinase*, *Phosphoglucomutase*, *UTP-glucose-1-phosphate uridylyltransferase*, *sucrose-phosphate synthase*, and *tonoplast monosaccharide transporter* (Fig. 2d, Additional file 3: Table S7). Moreover, ABA was positively correlated with its biosynthetic genes, including *Phytoene synthase* (PSY), *Zeta-carotene desaturase* (*ZDS*), *beta-ring hydroxylase* (*HYB*), *Short-chain dehydrogenase/reductase* (*SDR*), and *Aldehyde oxidase* (*AO*) (Fig. 2e, Additional file 3: Table S7). These results suggest that the gene-metabolite database accurately identifies crucial genes involved in previously reported metabolic pathways and thus provides valuable insights into the metabolic regulatory network within fruit flesh.

To survey the potential regulation of various metabolism pathways, the metabolic regulatory networks for different classes of metabolites within each cluster were constructed (Fig. 2f). The result showed that 4764 genes were correlated with at least two classes of metabolites, and among them, 2390 genes were involved in at least two clusters (Additional file 3: Table S7). For instance, out of the 10 ABA biosynthetic genes detected, 8 of them were also correlated with sucrose and glucose (Fig. 2f; Additional file 3: Table S7). This result is consistent with the previous report that ABA can increase soluble sugar content [46]. In addition, by mapping the genes in the database onto the chromosomes of the cv. Dangshansuli genome, visualization of this database provided evidence that the accumulation of each metabolite class is governed by the cooperatively regulation of genes located on multiple chromosomes (Additional file 1: Fig. S6).

### Effect of DNA methylation on gene expression

**Fig. 3:**
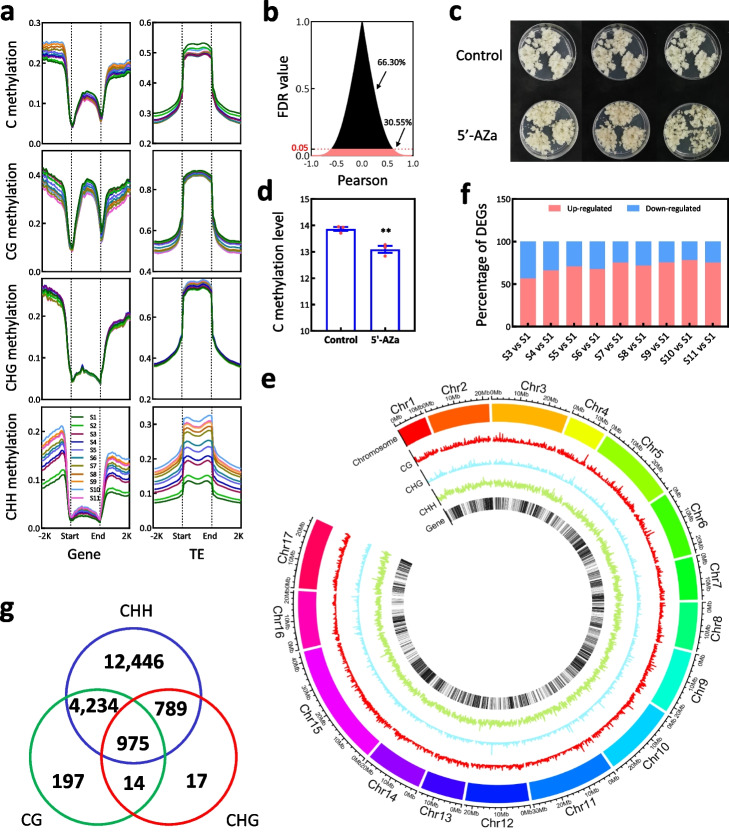
The involvement of DNA methylation in gene transcript during pear flesh development. **a** DNA methylation levels of genes and TEs in the fruit flesh at 11 stages (from S1 to S11). **b** Analysis of the differentially expressed genes (DEGs) correlated with DNA methylation in the promoter. The percentage represents the proportion of the DEGs that were correlated or uncorrelated with DNA methylation in the promoter. **c** Flesh callus was treated by the DNA methylation inhibitor 5-azacytidine (5’-Aza). Control is the untreated callus. **d** Genome-wide bisulfite sequencing of 5’-Aza-treated and control calli revealed the changes in DNA methylation. Standard error bars were calculated based on three replicates. Analysis of variance were calculated by Student’s *t*-test. Single asterisk stand for the level of significance at *P*-value < 0.05. **e** Chromosome locations of DNA methylation sites and all predicted genes revealed the genes modified by DNA methylation of all three context (CG, CHG, and CHH). Chr 1ꟷ17 represent the 17 chromosomes in pear genome. **f** A diagram showed the ratio of upregulated and downregulated genes between S1 and later stages. These genes have been identified to be repressed by 5’-Aza treatment in fruit callus and to be modified by DNA methylation in developing fruits. **g** A venn diagram showed the number of the DEGs modified by CG, CHG, and/or CHH methylations

To investigate the dynamics of DNA methylation in the fruit flesh, genome-wide bisulfite sequencing was performed on flesh samples at each stage and generated 866.75 million raw reads across all samples. Approximately 97.80% of these raw reads were used to examine DNA methylation sites in the whole genome, the number of clean bases obtained for each stage was more than 30 times larger than the size of the pear genome (Additional file 3: Table S9). As a result, a total of 39,016,459 sites were cytosine-methylated in the fruit flesh (Additional file 2: Table S1). On average, each stage displayed cytosine methylation at 27,345,777 sites, with 18,634,952 sites being consistently methylated across all stages. During the fruit flesh development, the DNA methylation level gradually increased in the whole genome and at the CHH region, while it gradually decreased at the CG region, and hardly changed at the CHG region (Additional file 3: Table S9). These trends were also observed in the gene body and its flanking regions, with the exception of a gradual decrease in cytosine methylation level within the gene body (Fig. 3a). Moreover, the CG methylation level gradually decreased in the two flanking regions of transposable elements (TE), while the CHH methylation level gradually increased in the TE body as well as its flanking regions (Fig. 3a). These results suggest that the increase in DNA methylation mainly is primarily driven by an elevation in CHH methylation. This finding is consistent with a previous report on sweet oranges [26].

To survey the effect of DNA methylation on gene expression, we investigated and found that cytosine methylation was present in the promoters of 38,159 genes including 20,322 DEGs (Additional file 2: Fig. S7a). Among these DEGs, the expression levels of 6209 DEGs were correlated with the average levels of cytosine methylation in their corresponding promoters, either positively or negatively (false discovery rate < 0.05; Fig. 3b, Additional file 3: Table S10). This suggests that DNA methylation may have both positive and negative effect on gene expression in pear fruit flesh. To verify this, pear flesh callus was treated with 50 mM 5-azacytidine (5’-AZa), a DNA methylation inhibitor (Fig. 3c). Genome-wide bisulfite sequencing of the 5’-Aza-treated and control (untreated) calli generated a total of 634.06 million raw reads (Additional file 4: Table S11) and showed that cytosine methylation level was reduced upon treatment with 5’-Aza (*p*-value = 0.0071 < 0.05; Fig. 3d). Subsequently, RNA sequencing of the 5’-Aza-treated and control calli generated a total of 267.34 million raw reads (Additional file 4: Table S12) and identified 1426 DEGs (Additional file 4: Table S13). A total of 58,943 differential methylation regions (DMRs) were detected from the promoters of 1328 DEGs, which included 705 DEGs present in the gene-metabolite database (Additional file 4: Table S13). These results suggest that the expression levels of these 1328 genes result from the change in DNA methylation in pear flesh callus.

To identify the methylation regions within the promoters of DEGs in fruit flesh, the methylation levels in a region enriched with cytosine methylation sites were analyzed between stages. A total of 178,499 DMRs were identified within the promoters of 35,176 genes in the fruit flesh (Fig. 3e, Additional file 2: Table S2). Among these DMRs, 92,912 were located within the promoters of 18,672 DEGs (Additional file 4: Table S14), including 12,396 DEGs that were correlated with 437 DAMs (Additional file 1: Fig. S7b, Additional file 4: Table S15). Out of the 12,396 DEGs, 647 were differentially expressed between the 5’-Aza-treated and control calli (Additional file 4: Table S13). After excluding the genes that were not differentially expressed between S1 and other stages, we found that 56.67 to 78.17% of genes, which were repressed by 5’-Aza treatment in fruit callus (Additional file 4: Table S13), were higher expressed in the developing fruits at later stages (from S3 to S11) compared to S1 (Fig. 3f). These results indicate that the expression of these 18,672 DEGs is likely modified by DNA methylation in the fruit flesh. Notably, 95.58% of these DEGs were modified by CHH DMR (Fig. 3g), suggesting that CHH DMR may play a more important role in modifying genome-wide gene expression in the fruit flesh compared to CG and CHG DMRs.

### Increased DNA methylation during flesh development is associated with decreased expression of the genes involved in DNA demethylation

To elucidate the underlying mechanism of increased DNA methylation during pear flesh development, we initially employed phylogenetic analyses to identify genes involved in the RdDM pathway (Additional file 1: Fig. S8a-d). Among these genes, *PbDCL2.2* was not expressed in fruit flesh (Additional file 4: Table S16). Overall, the expression levels of *PbAGO4.1*, *PbAGO4.2*, *PbAGO4.3*, *PbAGO6*, *PbDCL2.1*, *PbDCL3.1*, *PbDCL3.2*, *PbDCL3.3*, *PbDCL4*, *PbNRPD1*, *PbNRPE1*, *PbRDR2.1*, *PbRDR2.2*, and *PbRDR2.3* gradually decreased during flesh development (Additional file 1: Fig. S8e), and of which, most were negatively correlated with C and CHH methylation levels (Additional file 1: Fig. S8f). This result indicates that the decreased expression of these genes is not consistent with the increased DNA methylation during pear flesh development. In contrast, the expression levels of *PbDCL2.3*, *PbDCL2.4*, *PbDCL2.5*, and *PbDCL2.6* gradually increased during flesh development (Additional file 1: Fig. S8e), and notably, the expression level of *PbDCL2.3* was positively correlated with C and CHH methylation levels (Additional file 1: Fig. S8f). Theoretically, the increased expression of these *DCL* genes raises an abundance of RdDM-dependent 24-nt siRNA. To test this hypothesis, small RNAome sequencing was performed on fruit flesh at 11 stages. A total of 620.56 million raw reads were generated from the small RNA-Seq dataset, with 95.63% (clean reads) used for the analysis of 24-nt siRNA clusters (Additional file 4: Table S17). In total, 2,279,387 siRNAs were detected in the fruit flesh with an average of approximately 173,425 siRNAs detected at each stage, and of the 139,462 siRNAs were consistently present across all stages (Additional file 2: Table S1). As a whole, the levels of 24-nt siRNAs in the whole genome, as well as in CHH DMRs, CG DMRs, and CHG DMRs, were reduced in fruit flesh from S5 to S11 compared to those from S1 to S4 (Additional file 1: Fig. S9). This result shows that the expression of the four *DCL* genes does not result in an abundance of 24-nt siRNAs in the fruit flesh. Therefore, the changes in gene expression observed in RdDM pathway are not associated with the increase in DNA methylation during pear flesh development.

**Fig. 4:**
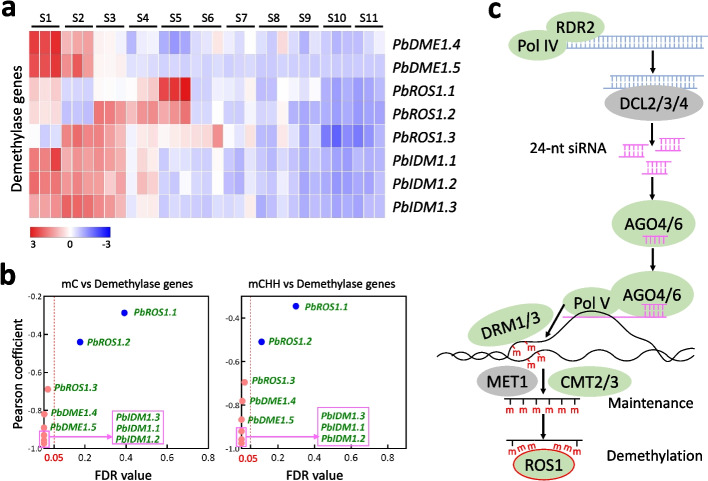
The decreased expression of demethylase genes is consistent with the increased DNA methylation during pear flesh development. **a** Expression patterns of demethylase genes in the fruit flesh at all 11 stages (S1ꟷS11). *Z*-score standardized values of each DEG across all 11 stages were used for clustering analysis. The color bar indicates the increasing expression levels of gene from blue to red. **b** Correlation analysis of demethylase genes with C (left panel) and CHH (right panel) methylations. The dotted line with red color represents the false discovery rate at 0.05. **c** A diagram showing the establishment, maintenance, and elimination of DNA methylation in fruit flesh

The maintenance of plant DNA methylation is governed by DNA methytransferases [27]. To examine the potential association between DNA methytransferases and the increased DNA methylation during pear flesh development, we isolated the 12 pear orthologs of *Arabidopsis* DNA methyltransferase genes from the pear genome (Additional file 1: Fig. S10a). The expression levels of the pear DNA methyltransferase genes, *PbDRM1.1*, *PbDRM1.2*, *PbDRM3*, *PbCMT2.1*, *PbCMT2.2*, *PbCMT2.3*, *PbCMT2.4*, *PbCMT3.1*, and *PbCMT3.2*, were higher in the fruit flesh at S1 and S2 compared to other stages (Additional file 1: Fig. S10b), and of which, most were negatively correlated with C and CHH methylation levels (Additional file 1: Fig. S10c). Moreover, the expression level of *PbMET1.2* was higher in the fruit flesh at S3 and S5 compared to S10 and S11 (Additional file 1: Fig. S10b), and was not correlated with C and CHH methylation levels (Additional file 1: Fig. S10c). These results indicate that the differential expression of these genes does not consistent with the increased DNA methylation during pear flesh development. In contrast, the expression level of *PbMET1.1* gradually increased during flesh development (Additional file 1: Fig. S10b) and was positively correlated with C and CHH methylation levels (Additional file 1: Fig. S10c). Notably, MET1.1 is responsible for maintaining CG cytosine methylation [27]. However, CG methylation level gradually decreased during pear flesh development (Fig. 4a) and was negatively correlated with the expression level of *PbMET1.1* (false discovery rate = 0.002 < 0.05). Therefore, the changes in the expression of the DNA methyltransferase genes are also not associated with the increased DNA methylation during pear flesh development.

The regulation of plant methylome involves the dynamic interplay between DNA methylation and DNA demethylation processes [27]. To determine the expression patterns of DNA demethylase genes in pear fruit flesh, we identified the eight pear orthologs of the *Arabidopsis ROS1* gene from the pear genome (Additional file 1: Fig. S11). Among these genes, *PbDME1.1*, *PbDME1.2*, and *PbDME1.3* were not expressed in the fruit flesh (Additional file 4: Table S16). Overall, the expression levels of *PbDME1.4*, *PbDME1.5*, and *PbROS1.3* gradually decreased during flesh development (Fig. 4a) and were negatively correlated with C and CHH methylation levels (Fig. 4b). Additionally, the expression levels of *PbROS1.1* and *PbROS1.2* gradually decreased in the fruit flesh from S5 to S11 (Fig. 4a). These results suggest that the decreased expression of these five genes is consistent with the increased DNA methylation during pear flesh development (Fig. 4c). Moreover, considering the role of *Arabidopsis* IDM1 in catalyzing histone H3K18 acetylation to create a chromatin environment that facilitates ROS1 function [35], we identified the three pear orthologs of *Arabidopsis IDM1* gene from the pear genome (Additional file 1: Fig. S11). The expression levels of *PbIDM1.1*, *PbIDM1.2*, and *PbIDM1.3* also gradually decreased during flesh development (Fig. 4a) and were negatively correlated with C and CHH methylation levels (Fig. 4b). Consequently, the decreased expression of all three *IDM* genes is consistent with the increased DNA methylation during flesh development. In conclusion, these findings suggest that the increased DNA methylation during pear flesh development may be contributed by the decreased expression of genes involved in DNA demethylation.

### DNA methylation is involved in pear fruit metabolism

It is determined that 12,396 DMR-modified DEGs correlated with 437 DAMs (Additional file 4: Table S15). To clarify the relationship between these DAMs and DNA methylation in the promoter of the DEGs, a correlation analysis was performed, revealing that DNA methylation in the promoters of 3987 DEGs was correlated (False discovery rate < 0.05) with 316 DAMs present in all eight clusters (Additional file 4: Table S18). Moreover, it was observed that DNA methylation in the whole genome was negatively correlated with DAMs in clusters I, II, and IV, but was positively correlated with DAMs in cluster V (Additional file 4: Table S19). These results indicate that DNA methylation likely plays a role in regulating fruit flesh metabolites by impacting expression of genes involved in metabolite production.

**Fig. 5:**
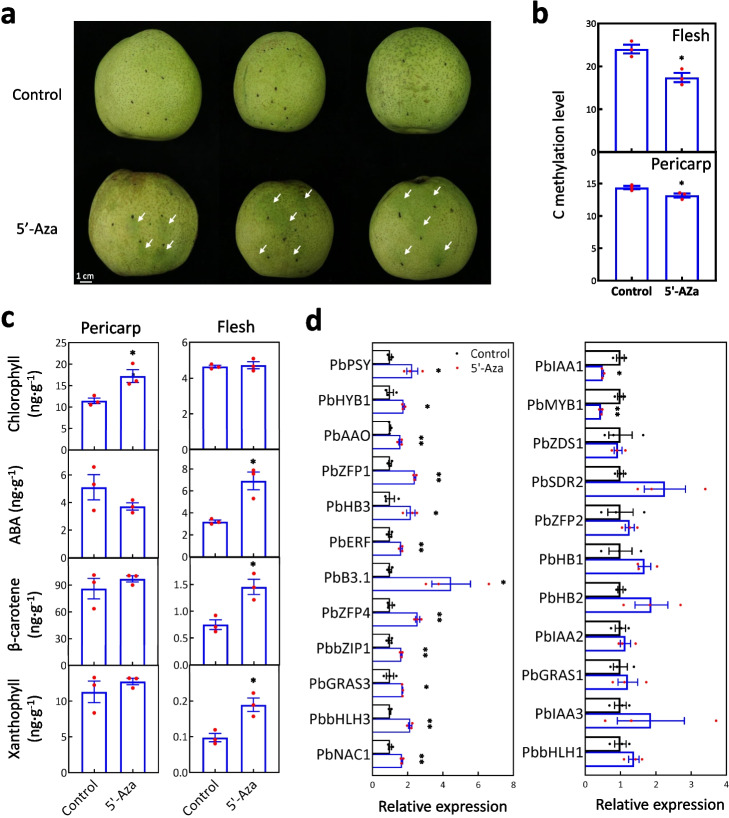
The involvement of DNA methylation in fruit flesh metabolism. **a** Phenotype change was detected in pear fruit treated with 5’-Aza compared to H_2_O (Control). White arrow indicates the dark green pericarp. **b** Genome-wide bisulfite sequencing of 5’-Aza-treated and control fruit revealed the changes in DNA methylation in pericarp and flesh of pear fruits. **c** Measurement of chlorophyll, ABA, β-carotene, and xanthophyll in the flesh and pericarp of the pear fruits treated with 5’-Aza and H_2_O (Control). **d** Expression levels of ABA biosynthetic genes and the positive correlated TFs were determined in the fruit flesh treated with 5’-Aza and H_2_O (Control). The standard error bars were calculated based on three replicates. Analysis of variance was calculated by Student’s *t* test. Single and double asterisks stand for the level of significance at *P*-value < 0.05 and < 0.01, respectively

Furthermore, a majority of the DMR-modified DEGs associated with the synthesis and metabolic processes of ABA, sucrose, and glucose were identified (Additional file 4: Table S15), and the DNA methylation in the promoters of partial DEGs, even in the whole genome, correlated with ABA, sucrose, and glucose (Additional file 4: Tables S18 and S19). ABA is an important phytohormone that promotes pear fruit ripening by increasing soluble sugar (including sucrose and glucose) content and ethylene release [46]. Therefore, DNA methylation may be involved in pear flesh development by influencing ABA biosynthesis. To test this hypothesis, pear fruit flesh was treated with 5’-Aza beneath the pericarp at S9 (Fig. 5a). Genome-wide bisulfite sequencing of the 5’-Aza-treated and control (H_2_O) pericarp generated a total of 674.03 raw reads (Additional file 4: Table S11) and showed a reduction in cytosine methylation level due to the treatment (*p*-value = 0.038 < 0.05; Fig. 5b). Compared to the control, the 5’-Aza treatment promoted chlorophyll accumulation in the pericarp (Fig. 5c), leading to a green pericarp (Fig. 5a). Moreover, genome-wide bisulfite sequencing of the 5’-Aza-treated and control (H_2_O) flesh generated a total of 679.69 raw reads (Additional file 4: Table S11) and showed a reduction in cytosine methylation level caused by the treatment (*p*-value = 0.012 < 0.05; Fig. 5b). Compared to the control, the 5’-Aza treatment promoted ABA, β-carotene, and xanthophyll biosynthesis in the flesh (Fig. 5c). This result suggests that DNA methylation is involved in carotenoid metabolism and perhaps accelerates fruit ripening by increasing ABA production.

To clarify the regulation of DNA methylation on carotenoid metabolism, quantitative real-time polymerase chain reaction (qRT-PCR) analysis was performed on the fruit flesh treated with 5’-Aza and H_2_O. Among the DMR-modified DEGs, 10 pathway genes and 35 TFs in expression level were positively correlated with ABA content (Additional file 4: Table S15). Out of these 10 pathway genes, *PbPSY*, *PbZDS1*, *PbHYB1*, *PbSDR2*, and *PbAAO* were expressed at higher levels in pear flesh from S5 to S11 compared to those from S1 to S4 (Additional file 1: Fig. S12). The expression levels of *PbPSY*, *PbHYB1*, and *PbAAO* were upregulated in the fruit flesh with the 5’-Aza treatment compared to the control, while no significant difference was observed for *PbZDS1* and *PbSDR2* expressions (Fig. 5d). These results suggest that DNA methylation represses the expressions of *PbPSY*, *PbHYB1*, and *PbAAO* to limit ABA production in fruit flesh. Of the 35 TFs, 18 were relatively highly expressed in the fruit flesh from S5 to S11 compared to other stages within their respective families (Additional file 1: Fig. S12). The expression levels of *PbZFP1*, *PbHB3*, *PbERF*, *PbB3.1*, *PbZFP4*, *PbbZIP1*, *PbGRAS3*, *PbbHLH3*, and *PbNAC1* were upregulated in the fruit flesh with the 5’-Aza treatment compared to the control, while no significant difference was observed for *PbZFP2*, *PbHB1*, *PbHB2*, *PbIAA2*, *PbIAA3*, *PbbHLH2*, and *PbbHLH1* expressions (Fig. 5d). These results suggest that the decreased DNA methylation may promote the expressions of several TFs to induce the expression of *PbPSY*, *PbHYB1*, and *PbAAO*. In addition, *PbIAA1* and *PbMYB1* were downregulated in the fruit flesh with the 5’-Aza treatment compared to the control (Fig. 5d), suggesting that the decreased DNA methylation can also inhibit gene expression.

### Verification of a novel transcription factor with roles in regulating ABA biosynthesis

**Fig. 6:**
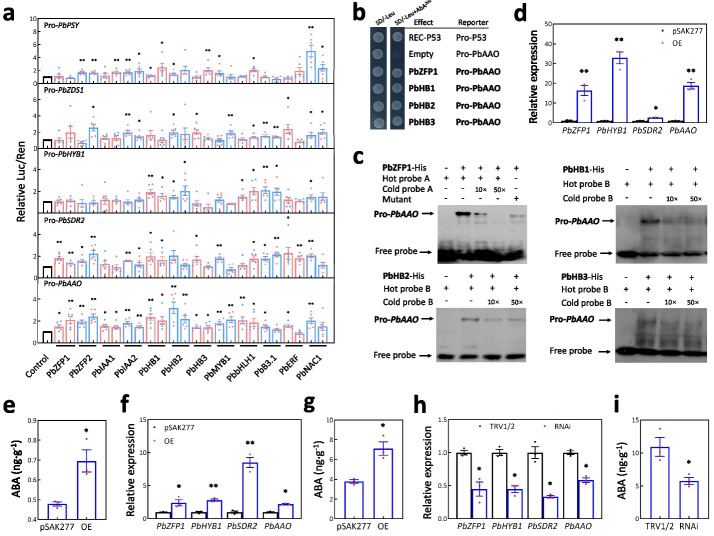
PbZFP1 positively mediates ABA biosynthesis in fruit flesh. **a** Dual-luciferase assays reveal the regulatory effects of 12 selected TFs on the promoters of ABA biosynthetic genes. The standard error bars were calculated from at least five replicates, and two independent experiments were performed. **b** A yeast-one-hybrid assay shows the binding of PbZFP1, PbHB1, PbHB2, and PbHB3 to the upstream region (− 500 bp to − 100 bp) of the initiation codon of *PbAAO*. SD/-Leu, SD medium lacking Leu; SD/Leu + AbA^200^, 200 ng/mL Aureobasidin A was added in the SD medium lacking Leu. **c** Electrophoretic mobility shift assays reveal the binding of the *PbAAO* promoter by PbZFP1, PbHB1, PbHB2, and PbHB3. “ + ” and “–” indicate the presence and absence of recombinant TF protein, biotin-labeled probe, cold probe, or biotin-labeled mutant, respectively. The concentrations of cold probe were tenfold (10 ×) and 50-fold (50 ×) of labeled probes. The expression level of *PbZFP1*, *PbHYB1*, *PbSDR2*, and *PbAAO* were tested by qRT-PCR in the transgenic calli (**d**) and transiently transformed fruit flesh (**f, h**). ABA content was measured in the transgenic calli (**e**) and transiently transformed fruit flesh (**g, i**). OE represents the over-expression of *PbZFP1*, while pSAK277 represents the empty vector and served as the control. RNAi represents the virus-induced gene silencing of *PbZFP1*, while TRV1/2 represents the empty vectors of pTRV1 and pTR2. Analysis of variance was calculated by Student’s *t* test. Single and double asterisks stand for the level of significance at *P*-value < 0.05 and < 0.01, respectively

To clarify the potential involvement of the tested TFs in ABA biosynthesis, a dual-luciferase assay was conducted using the promoters of ABA biosynthetic genes. A total of 12 TFs were selected based on expression and DNA methylation analyses (Fig. 5d, Additional file 4: Table S15). The result showed that the *LUC* gene, under the control of the *PbPSY*, *PbZDS1*, *PbHYB1*, *PbSDR2*, and *PbAAO* promoters, was significantly activated by at least three of the twelve TFs tested (Fig. 6a). This suggests that these 12 TFs are likely to activate a subset of genes involved in ABA biosynthesis.

AAO is a crucial enzyme catalyzing the conversion of abscisic aldehyde to ABA (Fig. 2e). The dual-luciferase assay had showed that the activity of the *PbAAO* promoter could be increased by nine TFs belonging to five different families (Fig. 6a). Of these families, ZFP, Homeobox, MYB, and bHLH TFs has been reported to be involved in fruit ripening [48–51]. To test the direct interaction between the identified TFs in these families and the *PbAAO* promoter, a dual-luciferase assay was performed and showed that the possible binding regions of PbMYB1 and PbbHLH1 were located within the upstream regions ranging from − 1000 bp to the initiation codon, and from − 2000 to − 500 bp of the initiation codon of *PbAAO*, respectively (Additional file 1: Figs. S13a, b). Further analysis showed that the possible binding regions of PbHB1 and PbHB2/PbHB3 were located within the upstream region ranging from − 300 bp to − 100 bp, and from − 200 bp to − 100 bp of the initiation codon of *PbAAO*, respectively (Additional file 1: Fig. S13c). In contrast, the identification of binding regions for PbZFP1 and PbZFP2 was challenging due to the distribution of predicted binding sites of C_2_H_2_-type ZFP in multiple positions within the *PbAAO* promoter (Additional file 1: Figs. S13 b-d). Yeast-one-hybrid (Y1H) assay showed that PbZFP1, PbHB1, PbHB2, and PbHB3 could bind to the upstream region ranging from − 500 bp to − 100 bp of the initiation codon of *PbAAO* (Fig. 6b). Electrophoretic mobility shift assay (EMSA) also showed that PbZFP1 directly interacted with probe A containing a predicted binding site of C_2_H_2_-type ZFP, while PbHB1, PbHB2, and PbHB3 could directly interact with probe B in the identified binding regions of the three HB TFs (Fig. 6c, Additional file 1: Fig. S13d). These results suggest that these TFs can physically interact with the *PbAAO* promoter to enhance its activity.

Moreover, the nucleus-localized *PbZFP1* was selected for function testing in ABA biosynthesis (Additional file 1: Fig. S14). Firstly, the over-expression vector of *PbZFP1*, driven by 35S promoter, was introduced into *A. tumefaciens* and used to infect pear fruit callus to produce the positive transgenic calli (Additional file 1: Fig. S15a). In the flesh calli over-expressing *PbZFP1*, the expression levels of *PbZFP1*, *PbHYB1*, *PbSDR2*, and *PbAAO*, as well as the ABA content, were significantly increased compared to the flesh calli infected with *A. tumefaciens* containing the empty vector pSAK277 (Fig. 6d, e). Secondly, *A. tumefaciens* containing the over-expression vector of *PbZFP1* was used to infiltrate the flesh of cv. Dangshansuli fruit at S9 (Additional file 2: Fig. S15b). The result showed that the expression levels of *PbZFP1*, *PbHYB1*, *PbSDR2*, and *PbAAO*, as well as the ABA content, were also increased in the fruit flesh over-expressing *PbZFP1*, compared to the fruit flesh infected by *A. tumefaciens* containing the empty vector pSAK277 (Fig. 6f, g). Finally, the virus-induced gene silencing (VIGS) vector of *PbZFP1* was introduced into *A. tumefaciens* and used to infiltrate the flesh of cv. Dangshansuli fruit at S9 (Additional file 1: Fig. S15c). The result showed that the expression levels of *PbZFP1*, *PbHYB1*, *PbSDR2*, and *PbAAO*, as well as the ABA content, were decreased in the fruit flesh over-expressing *PbZFP1*, compared to the fruit flesh infected by *A. tumefaciens* containing the empty vectors pTRV1 and pTRV2 (Fig. 6h, i). These results suggest that PbZFP1 positively mediates ABA biosynthesis in fruit flesh.

## Discussion

Fruit development is influenced by the accumulation and breakdown of various metabolites. The accumulation of metabolites during fruit development has been widely studied in fleshy fruits from pear to tomato, focusing initially on a few categories or classes of metabolites, such as sugars, organic acids, flavonoids, and polyphenols [6–8]. With the advent of broadly targeted metabolome methods, a large number of metabolites (> 400) were identified, providing a foundation for exploring fruit metabolism and its regulatory networks [2, 17]. In tomato, 980 distinct analytes, including 362 annotated metabolites, were identified from the pericarp of 442 accessions at the ripening stage, and of which, 371 metabolites were associated with 970 SNPs and 535 genes, as determined by the overlap of mGWAS and eQTL analyses [2]. A total of 540 metabolites detected across 20 major tomato tissues and growth stages were served for rewiring the tomato metabolic regulatory network by correlating them with genome-wide gene expression patterns [17]. In this study, the broadly targeted metabolome method was employed, leading to the identification of 492 annotated metabolites from pear fruit flesh at 11 stages, ranging from fruitlet to ripening. This number is close to the previously identified metabolites in peach fruit [5]. Moreover, 449 metabolites were differentially accumulated in fruit flesh at different stages (Additional file 2: Table S3). To explore the regulatory network of these DAMs, proteome and transcriptome analyses were performed to identify associated genes. Consequently, a gene-metabolite correlation database was constructed, encompassing 439 DAMs and 14,399 DEG (Additional file 3: Table S7), providing a valuable resource for investigating metabolic regulatory networks during pear flesh development.

The constructed gene-metabolite database allowed us to validate known metabolic regulatory networks and discover new ones. Previous studies have confirmed that anthocyanin production is mediated by the biosynthetic genes and MYB-bHLH-WD40 complex [52–54]. Herein, we observed similar expression patterns between the anthocyanin biosynthetic genes, components of MYB-bHLH-WD40 complex, and the content of peonidin-3-galactoside, a specific component of anthocyanin (Additional file 3: Table S7). This finding further supports the role of these genes and complex in anthocyanin biosynthesis. Moreover, the database served as an unbiased resource for identifying new TFs involved in ABA biosynthesis. ABA can increase soluble sugar content and ethylene release, promoting pear fruit ripening [44]. It is reported that several TF families, such as ERF, NAC, and TCP, positively mediate ABA production and fruit ripening [55–57]. In this study, we identified 37 TFs whose expression patterns correlated with ABA content (Additional file 3: Table S7). Among these TFs, PbZFP1, PbHB1, PbHB2, and PbHB3 directly bind to the *PbAAO* promoter to enhance its activity (Fig. 6a–c). Over-expression of *PbZFP1* in fruit flesh or flesh callus increases the *PbAAO* expression and ABA production (Fig. 6d–g), while knockdown of *PbZFP1* in fruit flesh decreases the *PbAAO* expression and ABA production (Fig. 6h, i). All these data indicate that PbZFP1 acts as a new regulator promoting ABA biosynthesis, and perhaps accelerating pear fruit ripening.

DNA methylation plays a significant role in gene expression and has been shown to regulate fruit ripening [24–26]. In tomato and strawberry, decreased DNA demethylation accelerates fruit ripening by upregulating ripening-induced genes and downregulating many ripening-repressed genes [24, 25]. In sweet orange, 5’-Aza treatment inhibits fruit ripening by increasing the expression of a ripening-induced gene Cs3g20300 [26]. Different from annual plants tomato and strawberry, as the long-lived woody perennial plants, pear fruit is formed by the expansion of receptacle, while orange fruit develops from the ovary. To survey the impact of DNA methylation on gene transcripts in pear fruit flesh, the DNA methylation inhibitor 5’-Aza was used to treat flesh callus and immature fruit flesh. In callus, 5’-Aza treatment decreased the level of DNA methylation, which was observed in 93.17% of the DEGs when comparing the 5'-Aza-treated calli to the control calli (Additional file 4: Table S13). In fruit flesh, we tested the expression level of 24 genes by qRT-PCR and found that 5’-Aza treatment led to the increased expression of 12 genes and the decreased expression of two genes (Fig. 5d). These results suggest that DNA methylation can regulate gene transcript during pear flesh development, either positively or negatively. Our results are consistent with previous reports on this subject [24, 26].

DNA methylation also plays a role in fruit metabolism, such as the accumulation of anthocyanins in peach fruit [58]. In this study, DMR-modified DEGs included 66.30% of all DEGs that were correlated with the 437 DAMs (Fig. 3b, Additional file 4: Table S15), indicating the involvement of DNA methylation in pear fruit metabolism. To confirm this, we measured the contents of ABA, β-carotene, and xanthophyll in 5’-Aza-treated and control flesh. The results showed significantly increased contents of these three metabolites in 5’-Aza-treated flesh compared to control (Fig. 5c). Meanwhile, 5’-Aza treatment enhanced the expression levels of 12 genes, including the ABA biosynthetic genes *PbPSY*, *PbHYB1*, and *PbAAO*, as well as transcription regulators *PbZFP1* and *PbHB3* (Fig. 5d). Therefore, DNA methylation is involved in fruit flesh metabolism by regulating the expression of ABA pathway genes during flesh development.

It is very interesting that both ABA content and DNA methylation level gradually increased during pear flesh development (Fig. 3a, Additional file 2: Table S3), but decreased DNA methylation promoted ABA accumulation (Fig. 5c). Similarly, both anthocyanin content and DNA methylation decreased in peach fruit under 0℃ condition compared to 16℃ condition, but decreased DNA methylation promoted anthocyanin accumulation [58]. These results differ from the previous reports. In strawberry, DNA methylation decreased during fruit ripening, and this decrease promoted fruit ripening [25]. In sweet orange, DNA methylation increased during fruit ripening, and this decrease inhibited fruit ripening [26]. These discrepancies suggest that the role of DNA methylation in fleshy fruit is elusive when assessed solely through correlation analysis with fruit phenotype. Based on these results, we observed a pattern in which DNA methylation increases during fruit development in woody perennial plants (pear and orange), but decreases in an annual plant (strawberry). Further evidence is necessary to verify whether this pattern is universally applicable to other woody and herbaceous plants.

**Fig. 7:**
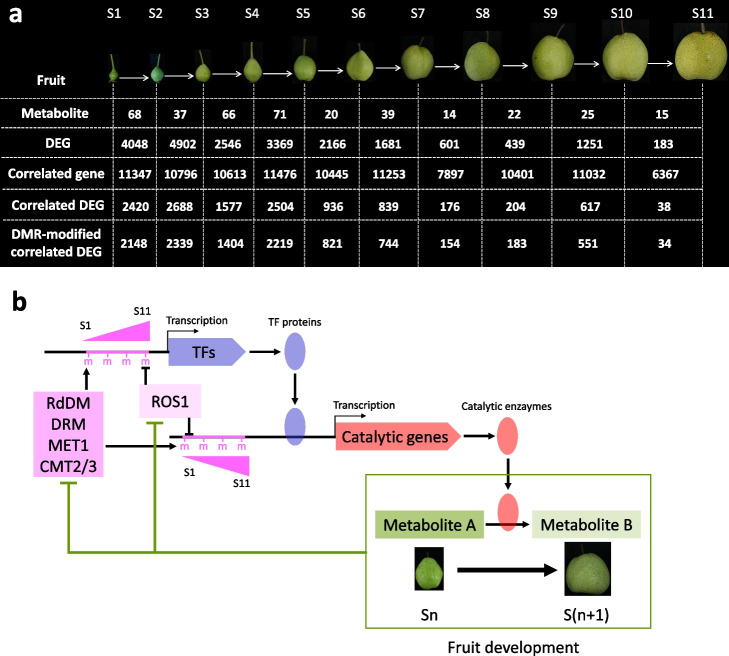
Summary of the metabolites, genes, and DNA methylation involved in pear flesh development. **a** The numbers of metabolites and genes were calculated between two adjacent stages during pear flesh development. DAM was calculated from metabolome data, while DEG was calculated from transcriptome data. Genes correlated with DAMs between adjacent stages was isolated from the gene-metabolite database and referred to as “correlated genes”. The intersection of DEGs and correlated genes is referred to as “Correlated DEGs.” Correlated DEGs that were modified by DNA methylation are denoted as “DMR-modified correlated DEG.” S1 to S11 indicate the different stages of pear flesh development. **b** A model shows the molecular pathway through which development-induced DNA methylation affects pear flesh development. During flesh development, TFs induce the expression of catalytic genes, leading to increased accumulation of catalytic enzymes that convert metabolite from one type (metabolite A) to another (metabolite B), thereby promoting the flesh development process. However, this process also represses the expression of demethylase, RdDM pathway, and methyltransferase genes, resulting in increased DNA methylation. This elevated DNA methylation affects the transcription of TFs and downstream catalytic genes, thereby influencing pear flesh development

The process of fruit development is a multifaceted phenomenon involving a series of physiological and metabolic changes, for which the molecular mechanism is extensively investigated. In this study, comprehensive analyses of the metabolome, transcriptome, proteome, and DNA methylome were performed in pear fruit flesh at 11 distinct stages to elucidate the metabolic regulatory network underlying flesh development. To identify DNA methylation-modified genes involved in the transition between consecutive stage, we employed differential analyses of metabolic, proteomic, and transcriptomic data to ascertain the DAMs and DEGs between each pair of adjacent stages (Additional file 2: Tables S3, S4, and S6), enabling the construction of a gene-metabolite correlation database (Additional file 3: Table S7). We found that the genes, correlated to the DAMs between two adjacent stages, encompass 20.77% (S11 vs S10) to 74.32% (S5 vs S4) of the DEGs between adjacent stages (designed as the correlated DEGs; Fig. 7a). In the correlated DEGs, most (> 87%) were modified by DMRs (Fig. 7a; Additional file 4: Table S20). The molecular network driving this transition can be delineated as follows (Fig. 7b): firstly, DNA methylation, subject to dynamic regulation by DNA methyltranferase and DNA demethylase, occurs within the promoters of target genes, thus influencing gene transcription. Secondly, TFs are transcribed under the effect of DNA methylation, and the resultant protein products bind the promoters of genes encoding catalytic enzymes. Thirdly, catalytic enzyme genes are transcribed under the effect of DNA methylation and TF proteins. Finally, the catalytic enzymes convert one metabolite to another, resulting in the transition from one stage to another until fruit ripening occurs. Throughout the progressive development of pear fruit flesh, most of the genes associated with DNA methylation and DNA demethylation are downregulated, leading to an overall increase in cytosine methylation.

## Conclusions

In summary, the establishment of a gene-metabolite database facilitates the exploration of metabolic regulatory networks, which serves as a basis for improving pear fruit quality. The increased DNA methylation during pear flesh development inhibits development-induced ABA accumulation, accomplished through the modulation of genes involving in the molecular network of ABA biosynthesis. This indicates that DNA methylation involves in flesh metabolism to mediate the process of pear flesh development, and perhaps delays fruit ripening.

## Methods

### Plant material

Pear cultivar Dangshansuli (*Pyrus bretschneideri*) is maintained at Jiangpu Orchard, Nanjing Agricultural University (Nanjing, China). The fruits at 11 distinct stages were harvested every 2 weeks, starting from 4 weeks after flower blooming until maturity. These fruits were collected from a 12-year-old pear tree. Each collection consisted of 12–16 fruits, which were further divided into three groups containing a minimum of four fruits per group. The fruits in each group were peeled, and their flesh was cut into small pieces and mixed together. The mixed samples were immediately frozen using liquid nitrogen and stored at − 80℃ until use.

### Measurements of metabolites

A total of 20 ± 0.2 g of the stored sample was subjected to freeze-drying, followed by weighting and grounding into a fine powder. Subsequently, 0.1 g of the resulting dry powder was dissolved in 1.2 mL of 70% methanol. To ensure a thorough suspension, the mixture was vortexed for 30 s, with a 30-min interval, and this process was repeated six times. After refrigerating the suspension overnight at 4 °C, it was then centrifugated at 12,000 rpm for 10 min. The resulting supernatant was filtered using a microporous member with a pore size of 0.22 μm. The filtrate obtained was used for measurements of broadly targeted metabolites. For the relative quantification of these metabolites, a liquid chromatography-electrospray ionization-tandem mass spectrometry system was employed, using a scheduled multiple reaction monitoring method [59, 60]. The monitored signal intensities were normalized by the internal standard (lidocaine, at a concentration of 0.1 mg l^−1^). Confirmation of the broadly targeted metabolites was achieved through the use of authentic standards. To ensure robustness, three biological replicates were performed for each sample.

The extraction and quantification procedures for carotenoid, ABA, and chlorophyll were performed in the same manner as previously described in reports pertaining to orange [61], strawberry [62], and pear [63] fruits, respectively. Carotenoid and ABA were identified using a high-performance liquid chromatography (Waters Crop., Milford, USA). The ABA standard was purchased from Sigma Company, while the xanthophyll and β-carotene standards were purchased from Yuanye Bio-Technology Company (Shanghai, China). Chlorophyll content was measured using a multimode reader (SpectraMax iD5, MD, USA). To ensure reliability, three biological replicates were performed for each sample.

### Protein extraction and sequencing

The flesh samples from each replicate were powdered in liquid nitrogen. Three biological replicates were performed. In each replicate, 150 mg of the freeze-dried powder was lysed in a solution (8 M urea, 1% SDS, 100 mM Tris–HCl, pH 8.0) containing a protease inhibitor cocktail (Sigma, St Louis, MO, USA). Following a 20 min incubation on ice, the supernatant was mixed with 4 volumes of 10 mM dithiothreitol (DTT) in cold acetone to extract proteins at a temperature of − 20℃ for the duration of overnight. The proteins were subsequently dissolved in a buffer (8 M urea, 100 mM Tris–HCl, pH 8.0). A total of 0.1 mg of proteins underwent a process involving DTT reduction and iodoacetamide alkylation, followed by digestion with Trypsin Gold (Promega, Madison, WI) at a temperature of 37℃ for a duration of 16 h. The resulting peptides were subjected to desalting suing a C18 cartridge and subsequently dried using vacuum centrifugation.

Peptide quantification was performed using liquid chromatography tandem mass spectrometry (LC–MS/MS) with a C18 column (150 µm inner-diameter, 360 µm outer-diameter × 15 cm, 1.9 µm C18, Reprosil-AQ Pur, Dr. Maisch). Data-dependent and data-independent acquisition protocols were employed, following a previous study [64] with few modifications. For a top 40 data-dependent acquisition (DDA) experiments, all MS scan resolutions and AGC target values were consistent with those used in the previous study [64]. The mass range was set from 350 to 1500 m/z, with a maximum ion injection time (max IT) of 45 ms, an isolation window of 1.6 m/z, and a normalized collision energy (CE) of 27%. For data-independent acquisition (DIA) experiments, the full MS scan resolutions were set to 60,000, with an AGC target value of 3E6 and an ion injection time of 50 ms. The mass range was set from 350 to 1200 m/z. For MS2, the resolutions were 30,000, with an AGC target value of 1E6 and an automatically adjusted ion injection time. The normalized CE remained at 27%.

The DDA data obtained were subjected to a separate database search against the pear protein database using Proteome Discoverer 2.2 (PD 2.2, Thermo). The parameters were set as follows: a mass tolerance of 10 ppm for precursor ion scans and 0.02 Da for the product ion scans. The fixed modification was carbamidomethyl, while the variable modifications contained oxidation of methionine (M) and acetylation of the N-terminus. Two mismatched sites were allowed. The results of the DDA search were used to build spectral libraries during the processing of DIA data, which were analyzed using the Skyline software package [65]. For each peptide, chromatograms were extracted from precursors with charge states of 2, 3, and/or 4. Chromatograms were also extracted from the precursor ions M, M1, and M2 of MS data, as well as from the y-ion series of the MS/MS data. The ion match tolerance was set at 0.05. The integration of chromatographic peak was evaluated based on two criteria: the similarity between the area of the precursor peaks and the distribution of theoretical isotope, and the similarity between area of fragment ion chromatograms from the DIA data and the spectral library. The integration of the five best product ion transitions was selected for testing. The error rates and feature scores were calculated using the mProphet algorithm [66] with the default parameters. Quantitative analysis of each peptide was performed using the MSstats program [67]. Peptides that could be mapped to multiple reference proteins were excluded. The expression patterns of all identified proteins are presented in Additional file 4: Table S21.

### mRNA sequencing

The total RNA from each sample was extracted using the RNAprep Pure Plant Kit of Polysaccharides & Polyphenolics-rich (Tiangen, Beijing, China). Three biological replicates were performed for each sample. The purity, concentration, and integrity of the extracted RNA samples were assessed, and only those with a higher RIN number (> 7) were used for library construction and sequencing. For mRNA sequencing library construction, ribosomal RNA was eliminated using the Epicentre Ribo-zero™ rRNA Removal Kit (Epicentre, USA). The remaining RNAs were processed using the rRNA-depleted RNA by NEBNext® Ultra™ Directional RNA Library Prep Kit for Illumina® (NEB, USA) to construct the sequencing library. The library was then sequenced on an Illumina Hiseq 4000 platform (Illumina) in 150 bp paired-end reads. The high-quality reads (clean reads) were mapped to the reference genome V1.0 of pear cultivar Dangshansuli (http://peargenome.njau.edu.cn/) using bowtie 2 v2.2.8 and HISAT2 v2.0.4 [68], allowing for three mismatches. The mapped reads from each sample were assembled using StringTie v1.3.1 [69] in a reference-based approach. The expression level of transcripts was calculated using Cufflinks v2.1.1 [70], based on the reads per kilobase of transcript per million mapped fragments (RPKM).

### Association analyses of metabolites, mRNAs, and proteins

Differential analysis was conducted to compare the metabolites, mRNAs, and proteins between each pair of stages. This analysis was based on two criterions: absolute fold-change ≥ 2 and a false discovery rate < 0.05. DAM was identified by evaluating the signal intensity of metabolites. DEP was identified based on the quantitative concentration of polypetides. DEG was analyzed using edgeR bioconductor package for R [71]. This analysis was based on the normalized count of reads mapped to the reference genome. Correlation analyses were performed in python script (https://github.com/Peims/calculate-the-pearsoner) [47] to examine the relationship between metabolites and mRNAs, proteins, and DNA methylation. The average value of three replicates was used in all the tested samples. The thresholds for significant correlation were set as a of Pearson coefficient > 0.85 or <  − 0.85 and a false discovery rate < 0.05.

### Genome-wide bisulfite sequencing

Genomic DNA was extracted using the CTAB-based method. The purity and concentration of the extracted DNA were checked using a Nanophotometer® spectrophotometer (IMPLEN, CA, USA) and the Qubit® DNA Assay Kit in Qubit 2.0 Flurometer (Life Technologies, CA, USA), respectively. To prepare the DNA for sequencing, it was fragmented into 200–300-bp fragments and subjected to end repair with adenylation. Subsequently, bisulfite treatment was performed twice using the EZ DNA Methylation-GoldTM Kit (Zymo Research, Irvine, CA, USA). The resulting single-strand DNA fragments were then amplified using the KAPA HiFi HotStart Uracil + ReadyMix (2X; Zymo Research) and sequenced on an Illumina Hiseq 4000 (Illumina, USA) in 150 bp paired-end reads. Following the removal of low-quality reads, the remaining reads were mapped to the pear reference genome v1.0 (http://peargenome.njau.edu.cn/) using Bismark software v0.16.3 [72], with a requirement of no mismatch allowed. Methylation sites were identified, and their levels were calculated according to a previously established methodology [73]. DMR was identified within the promoter of each gene using the DSS software [74].

### Small RNA sequencing

For a library of small RNA sequencing, total RNAs were processed using the NEBNext® Multiplex Small RNA Library Prep Set for Illumina® (NEB). Index codes were added to assign sequences to each sample, and two biological replicates were performed for each sample. Following the generation of clusters from the index-coded samples, the libraries were sequenced on an Illumina Hiseq 2500 platform (Illumina) in 50 bp single-end reads. After removing low-quality reads, the high-quality reads (clean reads) with length less than 18 bp and greater than 30 bp were discarded. The remaining clean reads were mapped to the pear reference genome using Bowtie [75], with no mismatches allowed. The 24-nt siRNAs were defined using ShortStack [76]. The abundance of 24-nt siRNAs was calculated by normalizing the mapped reads to the total clean reads.

### Protein-DNA interaction

The interaction relationship between the tested TFs and promoters was tested using the dual-luciferase, EMSA, and Y1H assays. For the dual-luciferase assay, the full-length coding sequences of each TF were inserted into the pSAK277 vector to a generate gene over-expression vector. Simultaneously, approximately 2000-bp promoter sequences were inserted into the pGreenII 0800-LUC vector. These constructs were then transformed into the *Agrobacterium tumefaciens* strain GV3101. Luminescence assays and measurement of firefly luciferase (LUC) and Renilla luciferase (REN) activities were performed according to a previously published protocol [3]. EMSA involved inserting the full-length coding sequences of each TF into the pCold-TF expression vector, which included a His tag, to create recombinant proteins. Biotin-labeled probes were synthesized by Sangon Biotech Company (Shanghai, China). EMSA experiments were carried out as previously described [77]. For the Y1H assay, the full-length coding sequences of each TF were inserted into the pGADT7 vector. The DNA fragment within the *PbAAO* promoter was amplified from the genomic DNA of “Dangshansuli” and subsequently inserted into the pAbAi vector. The Y1H assay was performed using the Matchmaker Gold Yeast One-Hybrid Library Screening System (Clontech, Palo Alto, CA). All primer sequences are listed in Additional file 4: Table S22.

### DNA methylation inhibitor treatment

The DNA methylation inhibitor 5’-Aza was used to treat cv. Dangshansuli fruit at S9 and cv. Clapp Favorite (*Pyrus communis*) flesh callus. In the fruit, approximately 200 μL of 40 mM solution of 5’-Aza (Sigma) dissolved in the double-distilled water was injected into the flesh along with pericarp. Images of the pear samples were captured at 10 days after injection. In the callus, young calli were treated with 50 mM 5’-Aza, and images of the calli samples were taken at 15 days after treatment.

To survey the effect of 5’-Aza treatment on DNA methylation, genomic DNA was extracted from the 5’-Aza-treated fruits and calli. Subsequently, the extracted genomic DNA was subjected to bisulfite treatment using the EZ DNA Methylation-GoldTM Kit (Zymo Research). DNA methylation levels were examined through genome-wide bisulfite sequencing.

To investigate the effect of 5’-Aza treatment on gene expression and carotenoid metabolism in callus, total RNAs were extracted from both wild and 5’-Aza-treated calli. The process of transcriptome sequencing and analysis was identical to that used for mRNA sequencing. The transcriptome reads were mapped to the reference genome of pear cultivar Bartllet DH (*Pyrus communis*; https://www.rosaceae.org/). Moreover, the contents of ABA, carotenoid, and chlorophyll were measured in the 5’-Aza-treated fruit flesh and pericarp, and the significant difference was calculated within Microsoft Office Excel 2016 (Microsoft Corp., Albuquerque, New Mexico, USA).

### Phylogenetic analysis

The pear orthologs of DNA methyltransferase, DNA demethylase, and RdDM pathway genes in *Arabidopsis* were identified by performing BLAST searches in Phytozome (https://phytozome-next.jgi.doe.gov/) against the strawberry and orange genomes. Additionally, the pear genome was also searched to identify these orthologs. The amino acid sequences of the identified genes were used to construct Neighbor-Joining phylogenetic trees using MEGA version 6. The bootstrap values were calculated based on 1000 replicate analyses.

### Transformation in pear fruit

To perform the over-expression study, the *Agrobacterium* harboring either the *PbZFP1* over-expression vector or the empty vector pSAK277 was used to infect the flesh calli of “Clapp’s Favorite,” following a previously reported protocol [78]. Similarly, for the injection study, *Agrobacterium* was used to inject the flesh of pear fruit at S9, as described in a previous report [77]. The calli were collected at 10 days after subculture, while the pericarp and flesh of the fruit were collected at 15 days after injection. For the virus-induced gene silencing (VIGS) assay, a specific fragment (ranging from 1260 to 1619 bp) was amplified and inserted into the pTRV2 vector. The constructed vector, along with pTRV1, was introduced into *A. tumefaciens* EHA105, which was then injected into the pear fruit at S9, following a previously reported procedure [77]. Co-injection of the *Agrobacterium* harboring pTRV1 and pTRV2 served as the negative control. The pericarp and flesh were collected at 20 days after injection. QRT-PCR was carried out in LightCycler 480® II/96 Thermal Cycler (Roche Diagostics). All analyses were achieved with three biological replicates. All primer sequences are listed in Additional file 4: Table S22. Moreover, the ABA content was measured in the transient over-expression and silencing fruit samples.

### Supplementary Information

**Additional file 1: Figure S1.** The changes of single fruit weight, longitudinal diameter, transverse diameter, stone cell, soluble sugars, ethylene, flesh firmness, and soluble solids during fruit development. S1 to S11 indicate the pear fruit at 4, 6, 8, 10, 12, 14, 16, 18, 20, 22, and 24 weeks after flower blooming, respectively. N/A represents unmeasurable data. **Figure S2.** Clustering analysis revealed that the profiling of differentially accumulated metabolites were grouped into eight groups, I → VIII. S1 to S11 indicate different stages. Z-score standardized values of each metabolite were used for clustering analysis. The y axis depicts the Z-score standardized value of each metabolite across all 11 stages. **Figure S3.** Analysis of the metabolites differentially accumulated between two adjacent stages. **a** Identification of the metabolites differentially accumulated between two adjacent stages. The metabolites above arrows are significantly increased in the subsequent stage when compared to the proceding stage. Conversely, the metabolites below arrows are significantly decreased. **b** The number and cluster of the metabolites differentially accumulated between two adjacent stages. **Figure S4.** Clustering analysis revealed the profiling and number of differentially expressed proteins (DEPs) correlating with the DAMs in eight clusters. **a** The expression patterns of DEPs correlating with the DAMs in eight clusters. The y axis depicts the Z-score standardized value of each protein across all 11 stages (from S1 to S11). The numbers in each box are the number of proteins in each cluster. **b** Two venn diagrams displays the numbers of DEPs among eight clusters. ‘Positive’ represents the positive correlation (Pearson coefficient > 0.85 and false discovery rate < 0.05) of proteins and metabolites in a same cluster, while ‘negative’ represents the negative correlation (Pearson coefficient < -0.85 and false discovery rate < 0.05). I → VIII indicate the different clusters. **Figure S5.** Clustering analysis revealed the profiling and number of differentially expressed genes (DEGs) correlating with the DAMs in eight clusters. **a** The expression patterns of DEGs correlating with the DAMs in eight clusters. The y axis depicts the Z-score standardized value of each protein across all 11 stages (from S1 to S11). The numbers in each box are the number of proteins in each cluster. **b** Two venn diagrams displays the numbers of DEGs among eight clusters. ‘Positive’ represents the positive correlation (Pearson coefficient > 0.85 and false discovery rate < 0.05) of genes and metabolites in a same cluster, while ‘negative’ represents the negative correlation (Pearson coefficient < -0.85 and false discovery rate < 0.05). I → VIII indicate the different clusters. **Figure S6.** The number of DEGs correlated to the DAMs in each metabolic class were counted in each chromosome. Others contained all scaffolds that were unanchored into the 17 chromosomes (Chr 1ꟷ17). ‘Value’ represents the number of genes. The number of genes is positively correlated with the increasing size of the circles and the transition from blue to red color. **Figure S7.** Integrative analysis of DNA methylation and DEGs. **a** Identification of the DEGs modified by DNA methylation in the promoters. **b** Identification of the DEGs that are correlated with DAMs and are also modified by DMRs. **Figure S8.** Identification and expression analysis of RdDM pathway genes in the fruit flesh. **a** Phylogenetic analysis classified the members of *AGO* gene family into six groups, AGO1, AGO10, AGO5, AGO6, AGO4/8/9, and AGO2/3. **b** Phylogenetic analysis classified the members of *DCL* gene family into four groups, DCL1, DCL2, DCL3, and DCL4.** c** Phylogenetic analysis classified the components of POL IV and V into four groups, RPB1, RPC2, NRPD1, and NRPE1. **d** Phylogenetic analysis classified the members of *RDR* gene family into three groups, RDR1/2, RDR3/4/5, and RDR6. The genes in each phylogenetic tree derive from pear, strawberry, orange, and Arabidopsis genomes. **e** Expression patterns of RdDM pathway genes in the fruit flesh at all 11 stages (S1ꟷS11). Z-score standardized values of each DEG across all 11 stages were used for clustering analysis. The color bar indicates the increasing expression levels of gene from blue to red. **f** Correlation analysis of RdDM pathway genes with C (left panel) and CHH (right panel) methylations. The dotted line with red color represents the false discovery rate at 0.05. **Figure S9.** Boxplots showing 24-nt small interfering RNA (siRNA) enrichment at whole genome and DMRs of all three contexts. S1 to S11 indicate different stages. R1 and R2 represent the two replicates. **Figure S10.** Identification and expression analysis of DNA methyltransferase genes in the fruit flesh. **a** Phylogenetic analysis classified the DNA methyltransferase genes into three groups, CMT, MET1, and DRM. The genes in phylogenetic tree derive from pear, strawberry, orange, and Arabidopsis genomes. **b** Expression patterns of DNA methyltransferase genes in the fruit flesh at all 11 stages (S1ꟷS11). Z-score standardized values of each DEG across all 11 stages were used for clustering analysis. The color bar indicates the increasing expression levels of gene from blue to red. **c** Correlation analysis of DNA methyltransferase genes with C (left panel) and CHH (right panel) methylations. The dotted line with red color represents the false discovery rate at 0.05. **Figure S11.** Identification of DNA demethylase genes in pear. Phylogenetic analysis classified the DNA demethylase genes into two groups, DME/DML/ROS and IDM. The genes in phylogenetic tree derive from pear, strawberry, orange, and Arabidopsis genomes. **Figure S12.** Expression pattern of ABA biosynthetic genes and the TFs positively correlated with ABA production. S1 to S11 indicate different stages. **Figure S13.** Identification of the possible binding regions of multiple TFs in the *PbAAO* promoter. **a** The constructed reporters and effectors. **b** The *PbAAO* promoter was divided into four fragments (P1 to P4; top panel) and was then used for dual-luciferase assay (bottom panel) to determine the binding regions of PbZFP1, PbZFP2, PbHB1, PbHB2, PbHB3, PbMYB1, and PbbHLH1 in the *PbAAO* promoter. **c** The *PbAAO* promoter was further divided into four additional fragments (P5 to P8; top panel) and was then used for dual-luciferase assay (bottom panel) to narrow the binding regions of PbZFP1, PbZFP2, PbHB1, PbHB2, and PbHB3 in the *PbAAO* promoter. Analysis of variance were calculated by Student’s *t* test. Single and double asterisks stand for the level of significance at *P*-value < 0.05 and < 0.01, respectively. **d** Prediction of *cis*-elements of GRAS, MYB, bHLH, and ZFP TFs. Probes A to D are selected for EMSA. **Figure S14.** Subcellular localization of PbZFP1-GFP fusion protein. PbZFP1 represents the PbZFP1-GFP fusion protein, while control represents the GFP protein. **Figure S15.** Pear transformation of *PbZFP1* in pear flesh callus and fruit flesh. **a** Stable over-expression of PbZFP1 (OE) in flesh callus. **b** Transient over-expression of PbZFP1 (OE) in fruit flesh. pSAK277 is the empty vector and used for the control of OE. **c** Virus-induced gene silencing of PbZFP1 (RNAi) in fruit flesh. TRV1/2 represents the empty vectors of pTRV1 and pTR2 and was used as the control of RNAi.

**Additional file 2: Table S1**: The isolated metabolites, proteins, genes, DNA methylation sites, and 24-nt siRNA clusters in pear fruit flesh at each stage. **Table S2**: The differential analysis of metabolites, proteins, genes, and DMRs between each pair of stages. **Table S3**: The differential accumulated metabolites during pear flesh development. **Table S4**: Identification of the proteins correlated with DAMs. **Table S5**: The transcriptome-sequenced reads and their mapping results in pear. **Table S6** Identification of the genes correlated with DAMs.

**Additional file 3: Table S7**: A gene-metabolite database was constructed by integrative analysis of DAMs, DAP, and DEGs. **Table S8**: KEGG enrichments of the genes correlated with the corresponding compound. **Table S9**: The DNA methylation-sequenced reads and their mapping results in pear flesh. **Table S10**: Identification of the genes modified by DNA methylation in pear flesh.

**Additional file 4: Table S11**: The DNA methylation-sequenced reads and their mapping results in flesh callus. **Table S12**: The transcriptome-sequenced reads of flesh calli and their mapping results in pear. **Table S13**: The differentially expressed genes observed between the 5'-Aza-treated and control calli. **Table S14**: Identification of the DEGs modified by DNA methylation. **Table S15**: Integrative analysis of DAMs, DEGs, and DMRs. **Table S16**: Expression levels of methyltransferase, RdDM pathway, and demethylase genes in pear flesh at 11 stages. **Table S17**: The small RNA sequenced reads and their mapping results in pear flesh. **Table S18**: Correlation analysis of DAMs and cytosine methylation in the promoters of DEGs. **Table S19**: Correlation analysis of DAMs and DNA methylation in whole genome. **Table S20**: Isolation of the DNA methylation-modified DEGs correlated with the DAMs between two adjacent stages. **Table S21**: The expression patterns of all identified proteins in pear flesh. **Table S22**: Primers used in this study.

**Additional file 5.** The uncropped picture for the EMSA image for ZFP1 in Fig. 6c.

**Additional file 6.** The uncropped picture for the EMSA image for HB1 in Fig. 6c.

**Additional file 7.** The uncropped picture for the EMSA image for HB2 in Fig. 6c.

**Additional file 8.** The uncropped picture for the EMSA image for HB3 in Fig. 6c.

**Additional file 9.** Review history.

## Data Availability

Transcriptome, DNA methylome, and small RNAome sequencing reads of the fruit flesh at all 11 stages were deposited into the NCBI BioProject under the accession numbers PRJNA1074939  [79], PRJNA838771 [80], and PRJNA838797 [81], respectively. The mass spectrometry proteomics and metabolomics data have been deposited to the ProteomeXchange Consortium (https://proteomecentral.proteomexchange.org) via the iProX partner repository [82, 83] with the dataset identifier PXD049025 [84]. Transcriptome and DNA methylome of the 5’-Aza-treated and control flesh calli were deposited into the NCBI BioProject under the accession numbers PRJNA1007363 [85] and PRJNA1015450  [86]. DNA methylome of the 5’-Aza-treated and control fruits were deposited into the NCBI BioProject under the accession numbers PRJNA1007412 [87] and PRJNA1007414  [88]. The reference genomes of cvs. Dangshansuli and Bartllet DH was accessed from the Pear Genome Project  [89] and Genome Database for Rosaceae  [90], respectively. The gene-metabolite database was deposited in Zenodo repository with the DOI number of https://doi.org/10.5281/zenodo.10674225 [91]. Data supporting the findings of this work are available within the paper and its Supplementary Information files. No other scripts and software were used other than those mentioned in the “Methods” section.

## References

[1] Ara T, Sakurai N, Takahashi S, Waki N, Suganuma H, Aizawa K, Matsumura Y, Kawada T, Shibata D (2021). TOMATIMET: A metabolome database consists of 7188 accurate mass values detected in mature fruits of 25 tomato cultivars. Plant Direct.

[2] Zhu G, Wang S, Huang Z, Zhang S, Liao Q, Zhang C, Lin T, Qin M, Peng M, Yang C, Cao X, Han X, Wang X, van der Knaap E, Zhang Z, Cui X, Klee H, Fernie AR, Luo J, Huang S (2018). Rewiring of the fruit metabolome in tomato breeding. Cell.

[3] Gu C, Xu HY, Zhou YH, Yao JL, Xie ZH, Chen YY, Zhang SL (2020). Multiomics analyses unveil the involvement of microRNAs in pear fruit senescence under high- or low-temperature conditions. Hortic Res.

[4] Wang S, Hu T, Wan J, Chen W, Liu X, Luo J, Xu J, Zhang H (2016). Spatio-temporal distribution and natural variation of metabolites in citrus fruits. Food Chem.

[5] Ying H, Shi J, Zhang S, Pingcuo G, Wang S, Zhao F, Cui Y, Zeng X (2016). Transcriptomic and metabolomics profiling provide novel insights into fruit development and flesh coloration in *Prunus mira* Koehne, a special wild peach species. BMC Plant Biol.

[6] Acquavia MA, Pascale R, Foti L, Carlucci G, Scrano L, Martelli G, Brienza M, Coviello D, Bianco G, Lelario F (2021). Analytical methods for extraction and identification of primary and secondary metabolites of apple (*Malus domestica*) fruits: a review. Separations.

[7] Bae H, Yun SK, Jun JH, Yoon IK, Nam EY, Kwon JH (2014). Assessment of organic acid and sugar composition in apricot, plumcot, plum, and peach during fruit development. J App Bo Food Qual.

[8] Quinet M, Angosto T, Yuste-Lisbona FJ, Blanchard-Gros R, Bigot S, Martinez J-P, Lutts S (2019). Tomato fruit development and metabolism. Front Plant Sci.

[9] Molassiotis A, Tanou G, Filippou P, Fotopoulos V (2013). Protenomics in the fruit tree science arena: new insights into fruit defense, development, and ripening. Proteomics..

[10] Palma JM, Corpas FJ, del Río LA (2011). Proteomics as an approach to the understanding of the molecular physiology of fruit development and ripening. Proteomics.

[11] Buts K, Hertog ML, Ho QT, America AH, Cordewener J, Vercammen J, Carpentier SC, Nicolai B (2016). Influence of pre-harvest calcium, potassium and triazole application on the proteome of apple at harvest. J Sci Food Agric.

[12] Mata CI, Fabre B, Hertog MLA, Parsons HT, Deery MJ, Lilley KS, Nicolaï BM (2017). In-depth characterization of the tomato fruit pericarp proteome. Proteomics.

[13] Wang J, Liu J, Chen K, Li H, He J, Guan B, He L (2017). Comparative transcriptome and proteome profiling of two *Citrus sinensis* cultivars during fruit development and ripening. BMC Genomics.

[14] Wang R, Shu P, Zhang C, Zhang J, Chen Y, Zhang Y, Du K, Xie Y, Li M, Ma T, Zhang Y, Li Z, Grierson D, Pirrello J, Chen K, Bouzayen M, Zhang B, Liu M (2022). Integrative analyses of metabolome and genome-wide transcriptome reveal the regulatory network governing flavor formation in kiwifruit (*Actinidia chinensis*). New Phytol.

[15] Xiao L, Li T, Jiang G, Jiang X, Duan X (2019). Cell wall proteome analysis of banana fruit softening using iTRAQ technology. J Proteomics.

[16] Zhang HP, Su Y, Tu Q, Qin GH (2021). Quantitative proteomic analysis of pear (Pyrus pyrifolia cv. “Hosui”) flesh provides novel insights about development and quality characteristics of fruit. Planta..

[17] Li Y, Chen Y, Zhou L, You S, Deng H, Chen Y, Alseekh S, Yuan Y, Fu R, Zhang Z, Su D, Fernie AR, Bouzayen M, Ma T, Liu M, Zhang Y (2020). MicroTom Metabolic Network: Rewiring Tomato Metabolic Regulatory Network throughout the Growth Cycle. Mol Plant.

[18] Tohge T, Scossa F, Wendenburg R, Frasse P, Balbo I, Watanabe M, Alseekh S, Jadhav SS, Delfin JC, Lohse M, Giavalisco P, Usadel B, Zhang Y, Luo J, Bouzayen M, Fernie AR (2020). Exploiting Natural Variation in Tomato to Define Pathway Structure and Metabolic Regulation of Fruit Polyphenolics in the *Lycopersicum* Complex. Mol Plant.

[19] Wang Z, Meng D, Wang A, Li T, Jiang S, Cong P, Li T (2013). The methylation of the PcMYB10 promoter is associated with green-skinned sport in Max Red Bartlett pear. Plant Physiol.

[20] Sicilia A, Scialò E, Puglisi I, Lo Piero AR (2020). Anthocyanin Biosynthesis and DNA methylation dynamics in sweet orange fruit [Citrus sinensis. (Osbeck)] under cold stress. J Agri Food Chem..

[21] Zhang B, Tieman DM, Jiao C, Klee HJ (2016). Chilling-induced tomato flavor loss is associated with altered volatile synthesis and transient changes in DNA methylation. Proc Natl Acad Sci USA.

[22] Wei C, Liu H, Cao X, Zhang M, Li X, Chen K, Zhang B (2021). Synthesis of flavour-related linalool is regulated by *PpbHLH1* and associated with changes in DNA methylation during pear fruit ripening. Plant Biotechnol J.

[23] Daccord N, Celton JM, Linsmith G, Becker C, Choisne N, Schijlen E, van de Geest H, Bianco L, Micheletti D, Velasco R, Pierro EAD, Gouzy J, Rees DJG, Guérif P, Muranty H, Durel CE, Laurens F, Lespinasse Y, Gaillard S, Aubourg S, Quesneville H, Weigel D, van de Weg E, Troggio M, Bucher E (2017). High-quality de novo assembly of the apple genome and methylome dynamics of eary fruit development. Nat Genet.

[24] Lang Z, Wang Y, Tang K, Tang D, Datsenka T, Cheng J, Zhang Y, Handa AK, Zhu JK (2017). Critical roles of DNA demethylation in the activation of ripening-induced genes and inhibition of ripening-repressed genes in tomato fruit. Proc Natl Acad Sci USA.

[25] Cheng JF, Niu QF, Zhang B, Chen KS, Yang RH, Zhu JK, Zhang YJ, Lang ZB (2018). Downregulation of RdDM during strawberry fruit ripening. Genome Biol.

[26] Huang H, Liu RE, Niu QF, Tang K, Zhang B, Zhang H, Chen KS, Zhu JK, Lang ZB (2019). Global increase in DNA methylation during orange fruit development and ripening. Proc Natl Acad Sci USA.

[27] Zhang H, Lang Z, Zhu JK (2018). Dynamics and function of DNA methylation in plants. Nat Rev Mol Cell Biol.

[28] Law JA, Jacobsen SE (2010). Establishing, maintaining and modifying DNA methylation patterns in plants and animals. Nat Rev Genet.

[29] Zemach A, Kim MY, Silva P, Rodrigues JA, Dotson B, Brooks MD, Zilberman D (2010). Local DNA hypomethylation activates genes in rice endosperm. Proc Natl Acad Sci USA.

[30] Matzke MA, Mosher RA (2014). RNA-directed DNA methylation: an epigenetic pathway of increasing complexity. Nat Rev Genet.

[31] Zhang H, Zhu JK (2011). RNA-directed DNA methylation. Curr Opin Plant Biol.

[32] Gong Z, Morales-Ruiz T, Ariza RR, Roldan-Arjona T, David L, Zhu JK (2002). ROS1, a repressor of transcriptional gene silencing in Arabidopsis, encodes a DNA glycosylase/lyase. Cell.

[33] Ortega-Galisteo AP, Morales-Ruiz T, Ariza RR, Roldan-Arjona T (2008). Arabidopsis DEMETER-LIKE proteins DML2 and DML3 are required for appropriate distribution of DNA methylation marks. Plant Mol Biol.

[34] Tang K, Lang Z, Zhang H, Zhu JK (2016). The DNA demethylase ROS1 targets genomic regions with distinct chromatin modifications. Nat Plants.

[35] Qian W, Miki D, Zhang H, Liu Y, Zhang X, Tang K, Kan Y, La H, Li X, Li S, Zhu X, Shi X, Zhang K, Pontes O, Chen X, Liu R, Gong Z, Zhu JK (2012). A histone acetyltransferase regulates DNA demethylation in *Arabidopsis*. Science.

[36] Liu J, Zhou F, Cui S, Yang Y, Sun Q, Guan Q, Li D, Zhang S, Wang R (2022). Effects of ploidy variation on DNA methylation and gene expression in pear (Pyrus communis L.). Sci Hortic..

[37] Qian M, Sun Y, Allan AC, Teng Y, Zhang D (2014). The red sport of ‘Zaosu’ pear and its red-striped pigmentation pattern are associated with demethylation of the *PyMYB10* promoter. Phytochemistry.

[38] Zhai R, Wang Z, Yang C, Kui LW, Espley R, Liu J, Li X, Wu Z, Li P, Guan Q, Ma F, Xu L (2019). PbGA2ox8 induces vascular-related anthocyanin accumulation and contributes to red stripe formation on pear fruit. Hortic Res..

[39] Michalak M, Barciszewska MZ, Barciszewski J, Plitta BP, Chmielarz P (2013). Global changes in DNA methylation in seeds and seedlings of *Pyrus communis* after seed desiccation and storage. PLoS ONE.

[40] Li Q, Qiao X, Yin H, Zhou Y, Dong H, Qi K, Li L, Zhang S (2019). Unbiased subgenome evolution following a recent whole-genome duplication in pear (Pyrus bretschneideri Rehd.). Hortic Res..

[41] Romani RJ (1998). DNA methylation levels and ethylene production in senescent, suspension-cultured pear fruit cells: implications for epigenetic control?. Physiol Plant.

[42] Gong X, Qi K, Chen J, Zhao L, Xie Z, Yan X, Khanizadeh S, Zhang S, Tao S (2023). Multi-omics analyses reveal stone cell distribution pattern in pear fruit. Plant J.

[43] Weaver RJ, Shindy W, Kliewer M (1969). Growth regulator induced movement of photosynthetic products into fruits of `Black Corinth' grapes. Plant Physiol.

[44] Omel S, Ohmel G, Salama ASM (2014). Effect of proline and tryptophan amino acids on yield and fruit quality of Manfalouty pomegranate variety. Sci Hortic..

[45] Oikawa A, Otsuka T, Nakabayashi R, Jikumaru Y, Isuzugawa K, Murayama H, Saito K, Shiratake K (2015). Metabolic profiling of developing pear fruits reveals dynamic variation in primary and secondary metabolites, including plant hormones. PLoS ONE.

[46] Dai S, Li P, Chen P, Li Q, Pei Y, He S, Sun Y, Wang Y, Kai W, Zhao B, Liao Y, Leng P (2014). Transcriptional regulation of genes coding ABA metabolism enzymes during the fruit development and dehydration stress of pear ‘Gold Nijisseiki’. Plant Physiol Biochem.

[47] Pei M-S, Cao S-H, Wu L, Wang GM, Xie ZH, Gu C, Zhang S-L (2020). Comparative transcriptome analyses of fruit development among pears, peaches, and strawberries provide new insights into single sigmoid patterns. BMC Plant Biol.

[48] Fan ZQ, Ba LJ, Shan W, Xiao YY, Lu WJ, Kuang JF, Chen JY (2018). A banana R2R3-MYB transcription factor MaMYB3 is involved in fruit ripening through modulation of starch degradation by repressing starch degradation-related genes and MabHLH6. Plant J.

[49] Gu C, Guo ZH, Cheng HY, Zhou YH, Qi KJ, Wang GM, Zhang SL (2019). A HD-ZIP II HOMEOBOX transcription factor, PpHB.G7, mediates ethylene biosynthesis during fruit ripening in peach. Plant Sci..

[50] Han YC, Fu CC, Kuang JF, Chen JY, Lu WJ (2016). Two banana fruit ripening-related C2H2 zinc finger proteins are transcriptional repressors of ethylene biosynthetic genes. Postharvest Biol Tec.

[51] Zhu ZG, Chen GP, Guo XH, Yin WC, Yu XH, Hu JT, Hu ZL (2017). Overexpression of SlPRE2, an atypical bHLH transcription factor, affects plant morphology and fruit pigment accumulation in tomato. Sci Rep..

[52] Hichri I, Barrieu F, Bogs J, Kappel C, Delrot S, Lauvergeat V (2011). Recent advances in the transcriptional regulation of the flavonoid biosynthetic pathway. J Exp Bot.

[53] Xu W, Dubos C, Lepiniec L (2015). Transcriptional control of flavonoid biosynthesis by MYB-bHLH-WDR complexes. Trends Plant Sci.

[54] Zhang Y, Butelli E, Martin C (2014). Engineering anthocyanin biosynthesis in plants. Curr Opin Plant Biol.

[55] Meng C, Yang D, Ma X, Zhao W, Liang X, Ma N, Meng Q (2016). Suppression of tomato *SlNAC1* transcription factor delays fruit ripening. J Plant Physiol.

[56] Wang X, Zeng W, Ding Y, Niu L, Yao J-L, Pan L, Lu Z, Cui G, Li G, Wang Z (2019). PpERF3 positively regulates ABA biosynthesis by activating PpNCED2/3 transcription during fruit ripening in peach. Hortic Res.

[57] Xie YG, Ma YY, Bi PP, Wei W, Liu J, Hu Y, Gou YJ, Zhu D, Wen YQ, Feng JY (2020). Transcription factor FvTCP9 promotes strawberry fruit ripening by regulating the biosynthesis of abscisic acid and anthocyanins. Plant Physiol Biochem.

[58] Zhu YC, Zhang B, Allan AC, Lin-Wang K, Zhao Y, Wang K, Chen KS, Xu CJ (2020). DNA demethylation is involved in the regulation of temperature-dependent anthocyanin accumulation in peach. Plant J.

[59] Chen W, Gong L, Guo Z, Wang W, Zhang H, Liu X, Yu S, Xiong L, Luo J (2013). A novel integrated method for large-scale detection, identification, and quantification of widely targeted metabolites: application in the study of rice metabolomics. Mol Plant.

[60] Chen W, Wang W, Peng M, Gong L, Gao Y, Wan J, Wang S, Shi L, Zhou B, Li Z, Peng X, Yang C, Qu L, Liu X, Luo J (2015). Comparative and parallel genome-wide association studies for metabolic and agronomic traits in cereals. Nat Comm.

[61] Liu Q, Xu J, Liu Y, Zhao X, Deng X, Guo L, Gu J (2007). A novel bud mutation that confers abnormal patterns of lycopene accumulation in sweet orange fruit (Citrus sinensis L. Osbeck). J Exp Bot..

[62] Jia HF, Chao YM, Li CL, Lu D, Luo JJ, Qin L, Shen YY. Abscisic acid plays an important role in the regulation of strawberry fruit ripening. Plant Physiol. 2011;157:188–99. 10.1104/pp.111.177311 PMC3165869 21734113

[63] Cheng Y, Dong Y, Yan H, Ge W, Shen C, Guan J, Liu L, Zhang Y (2012). Effects of 1-MCP on chlorophyll degradation pathway-associated genes expression and chloroplast ultrastructure during the peel yellowing of Chinese pear fruits in storage. Food Chem.

[64] Bekker-Jensen DB, Bernhardt OM, Hogrebe A, Martinez-Val A, Verbeke L, Gandhi T, Kelstrup CD, Reiter L, Olsen JV (2020). Rapid and site-specific deep phosphoproteome profiling by data-independent acquisition without the need for spectral libraries. Nat Commun.

[65] MacLean B, Tomazela DM, Shulman N, Chambers M, Finney GL, Frewen B, Kern R, Tabb DL, Liebler DC, MacCoss MJ (2010). Skyline: an open source document editor for creating and analyzing targeted proteomics experiments. Bioinformatics.

[66] Reiter L, Rinner O, Picotti P, Hüttenhain R, Beck M, Brusniak MY, Hengartner MO, Aebersold B (2011). mProphet: automated data processing and statistical validation for large-scale SRM experiments. Nat Methods.

[67] Choi M, Chang CY, Clough T, Broudy D, Killeen T, MacLean B, Vitek O (2014). MSstats: an R package for statistical analysis of quantitative mass spectrometry-based proteomic experiments. Bioinformatics.

[68] Langmead B, Salzberg SL (2012). Fast gapped-read alignment with Bowtie 2. Nat Methods.

[69] Pertea M, Kim D, Pertea GM, Leek JT, Salzberg SL (2016). Transcript-level expression analysis of RNA-seq experiments with HISAT. StringTie and Ballgown Nat Protoc.

[70] Trapnell C (2010). Transcript assembly and quantification by RNA-Seq reveals unannotated transcripts and isoform switching during cell differentiation. Nat Biotechnol.

[71] Robison MD, Oshlack A (2010). A scaling normalization method for differential expression analysis of RNA-seq data. Genome Biol.

[72] Krueger F, Andrews SR. Bismark: a flexible aligner and methylation caller for Bisulfite-Seq applications. Bioinformatics. 2011;27:1571–2. 10.1093/bioinformatics/btr167 PMC3102221 21493656

[73] Lister R, Mukamel EA, Nery JR, Urich M, Puddifoot CA, Johnson ND, Lucero J, Huang Y, Dwork AJ, Schultz MD, Yu M, Tonti-Felippini J, Heyn H, Hu S, Wu JC, Rao A, Esteller M, He C, Haghighi FG, Sejnowski TJ, Behrens MM, Ecker JR (2013). Global epigenomic reconfiguration during mammalian brain development. Science.

[74] Wu H, Xu T, Feng H, Chen L, Li B, Yao B, Qin Z, Jin P, Conneely KN (2015). Detection of differentially methylated regions from whole-genome bisulfite sequencing data without replicates. Nucleic Acids Res.

[75] Langmead B, Trapnell C, Salzberg SL (2009). Ultrafast and memory-efficient alignment of short DNA sequences to the human genome. Genome Biol.

[76] Axtell MJ (2013). ShortStack: comprehensive annotation and quantification of small RNA genes. RNA.

[77] Guo ZH, Zhang YJ, Yao JL, Xie ZH, Zhang YY, Zhang SL, Gu C (2021). The NAM/ATAF1/2/CUC2 transcription factor PpNAC.A59 enhances PpERF.A16 expression to promote ethylene biosynthesis during peach fruit ripening. Hortic Res..

[78] Bai S, Tao R, Yin L, Ni J, Yang Q, Yan X, Yang F, Guo X, Li H, Teng Y (2019). Two B-box proteins, PpBBX18 and PpBBX21, antagonistically regulate anthocyanin biosynthesis via competitive association with *Pyrus pyrifolia* ELONGATED HYPOCOTYL 5 in the peel of pear fruit. Plant J.

[79] Gu C, Pei M-S, Guo, Z-H, Wu L, Qi, K-J, Wang X-P, Liu H, Liu Z, Lang Z, Zhang, S-L. Multi-omics studies provide a novel insight into the regulation of DNA methylation on pear fruit metabolism. PRJNA1074939. Transcriptome sequencing of pear fruit. https://www.ncbi.nlm.nih.gov/bioproject/?term=PRJNA1074939 (2024). 10.1186/s13059-024-03200-2 PMC10938805 38486226

[80] Gu C, Pei M-S, Guo, Z-H, Wu L, Qi, K-J, Wang X-P, Liu H, Liu Z, Lang Z, Zhang, S-L. Multi-omics studies provide a novel insight into the regulation of DNA methylation on pear fruit metabolism. PRJNA838771. DNA methylation sequencing of pear fruit. https://www.ncbi.nlm.nih.gov/bioproject/?term=PRJNA838771 (2022). 10.1186/s13059-024-03200-2 PMC10938805 38486226

[81] Gu C, Pei M-S, Guo, Z-H, Wu L, Qi, K-J, Wang X-P, Liu H, Liu Z, Lang Z, Zhang, S-L. Multi-omics studies provide a novel insight into the regulation of DNA methylation on pear fruit metabolism. PRJNA838797. Small RNA sequencing of pear fruit. https://www.ncbi.nlm.nih.gov/bioproject/?term=PRJNA838797 (2022). 10.1186/s13059-024-03200-2 PMC10938805 38486226

[82] Ma J, Chen T, Wu S, Yang C, Bai M, Shu K, Li K, Zhang G, Jin Z, He F, Hermjakob H, Zhu Y (2019). iProX: an integrated proteome resource. Nucleic Acids Res.

[83] Chen T, Ma J, Liu Y, Chen Z, Xiao N, Lu Y, Fu Y, Yang C, Li M, Wu S, Wang X, Li D, He F, Hermjakob H, Zhu Y (2022). iProX in 2021: connecting proteomics data sharing with big data. Nucleic Acids Res.

[84] Gu C, Pei M-S, Guo, Z-H, Wu L, Qi, K-J, Wang X-P, Liu H, Liu Z, Lang Z, Zhang, S-L. Multi-omics studies provide a novel insight into the regulation of DNA methylation on pear fruit metabolism. PXD049025. Multiple-omics studies of pear fruit flesh. https://www.iprox.cn/page/project.html?id=IPX0008046000 (2024). 10.1186/s13059-024-03200-2 PMC10938805 38486226

[85] Gu C, Pei M-S, Guo, Z-H, Wu L, Qi, K-J, Wang X-P, Liu H, Liu Z, Lang Z, Zhang, S-L. Multi-omics studies provide a novel insight into the regulation of DNA methylation on pear fruit metabolism. PRJNA1007363. DNA methylation in pear flesh callus. https://www.ncbi.nlm.nih.gov/bioproject/?term=PRJNA1007363 (2023). 10.1186/s13059-024-03200-2 PMC10938805 38486226

[86] Gu C, Pei M-S, Guo, Z-H, Wu L, Qi, K-J, Wang X-P, Liu H, Liu Z, Lang Z, Zhang, S-L. Multi-omics studies provide a novel insight into the regulation of DNA methylation on pear fruit metabolism. PRJNA1015450. Transcriptome analysis of pear flesh callus with 5'-AZa treatment. https://www.ncbi.nlm.nih.gov/bioproject/?term=PRJNA1015450 (2023).

[87] Gu C, Pei M-S, Guo, Z-H, Wu L, Qi, K-J, Wang X-P, Liu H, Liu Z, Lang Z, Zhang, S-L. Multi-omics studies provide a novel insight into the regulation of DNA methylation on pear fruit metabolism. PRJNA1007412. DNA methylation in pear pericarp. https://www.ncbi.nlm.nih.gov/bioproject/?term=PRJNA1007412 (2023). 10.1186/s13059-024-03200-2 PMC10938805 38486226

[88] Gu C, Pei M-S, Guo, Z-H, Wu L, Qi, K-J, Wang X-P, Liu H, Liu Z, Lang Z, Zhang, S-L. Multi-omics studies provide a novel insight into the regulation of DNA methylation on pear fruit metabolism. PRJNA1007414. DNA methylation in pear flesh. https://www.ncbi.nlm.nih.gov/bioproject/?term=PRJNA1007414 (2023). 10.1186/s13059-024-03200-2 PMC10938805 38486226

[89] Wu J, Wang Z, Shi Z, Zhang S, Ming R, Zhu S, Khan MA, Tao S, Korban SS, Wang H, Chen NJ, et al. The genome of the pear (*Pyrus bretschneideri* Rehd.). Pear Genome Project. http://peargenome.njau.edu.cn (2013). 10.1101/gr.144311.112 PMC3561880 23149293

[90] Linsmith G, Rombauts S, Montanari S, Deng CH, Celton J-M, Guérif P, Liu C, et al. Pseudo-chromosome length genome assembly of a double haploid ‘Bartlett’ pear (*Pyrus communis* L.). Genome Database for Rosaceae. *Pyrus communis* Bartlett DH Genome v2.0. https://www.rosaceae.org/species/pyrus/pyrus_communis/genome_ v2.0 (2019). 10.1093/gigascience/giz138 PMC6901071 31816089

[91] Gu C, Zhang S-L. A gene-metabolite dataset of pear fruit development. 2024. Zenodo. 10.5281/zenodo.10674225.

