# Supplementary material for: Multi-omics provide insights into the regulation of DNA methylation in pear fruit metabolism
Source: Genome Biol. 2024 Mar 14;25:70. doi: 10.1186/s13059-024-03200-2 (PMC10938805; doi:10.1186/s13059-024-03200-2)
Supplement: Supplementary file 1 — Additional file 1: Figure S1. The changes of single fruit weight, longitudinal diameter, transverse diameter, stone cell, soluble sugars, ethylene, flesh firmness, and soluble solids during fruit development. S1 to S11 indicate the pear fruit at 4, 6, 8, 10, 12, 14, 16, 18, 20, 22, and 24 weeks after flower blooming, respectively. N/A represents unmeasurable data. Figure S2. Clustering analysis revealed that the profiling of differentially accumulated metabolites were grouped into eight groups, I → VIII. S1 to S11 indicate different stages. Z-score standardized values of each metabolite were used for clustering analysis. The y axis depicts the Z-score standardized value of each metabolite across all 11 stages. Figure S3. Analysis of the metabolites differentially accumulated between two adjacent stages. a Identification of the metabolites differentially accumulated between two adjacent stages. The metabolites above arrows are significantly increased in the subsequent stage when compared to the proceding stage. Conversely, the metabolites below arrows are significantly decreased. b The number and cluster of the metabolites differentially accumulated between two adjacent stages. Figure S4. Clustering analysis revealed the profiling and number of differentially expressed proteins (DEPs) correlating with the DAMs in eight clusters. a The expression patterns of DEPs correlating with the DAMs in eight clusters. The y axis depicts the Z-score standardized value of each protein across all 11 stages (from S1 to S11). The numbers in each box are the number of proteins in each cluster. b Two venn diagrams displays the numbers of DEPs among eight clusters. ‘Positive’ represents the positive correlation (Pearson coefficient > 0.85 and false discovery rate < 0.05) of proteins and metabolites in a same cluster, while ‘negative’ represents the negative correlation (Pearson coefficient < -0.85 and false discovery rate < 0.05). I → VIII indicate the different clusters. Figure S5. Clu [file 13059_2024_3200_MOESM1_ESM.docx]

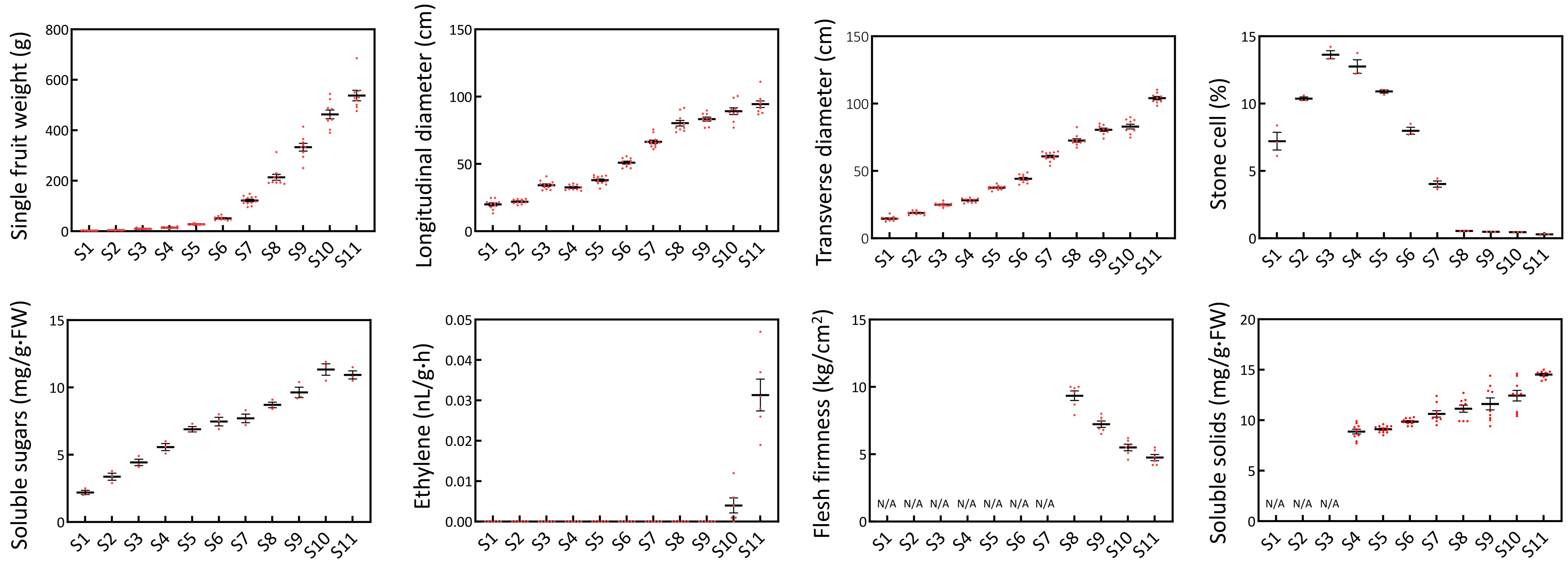


**Figure S1.** The changes of single fruit weight, longitudinal diameter, transverse diameter, stone cell, soluble sugars, ethylene, flesh firmness, and soluble solids during fruit development. S1 to S11 indicate the pear fruit at 4, 6, 8, 10, 12, 14, 16, 18, 20, 22, and 24 weeks after flower blooming, respectively. N/A represents unmeasurable data.


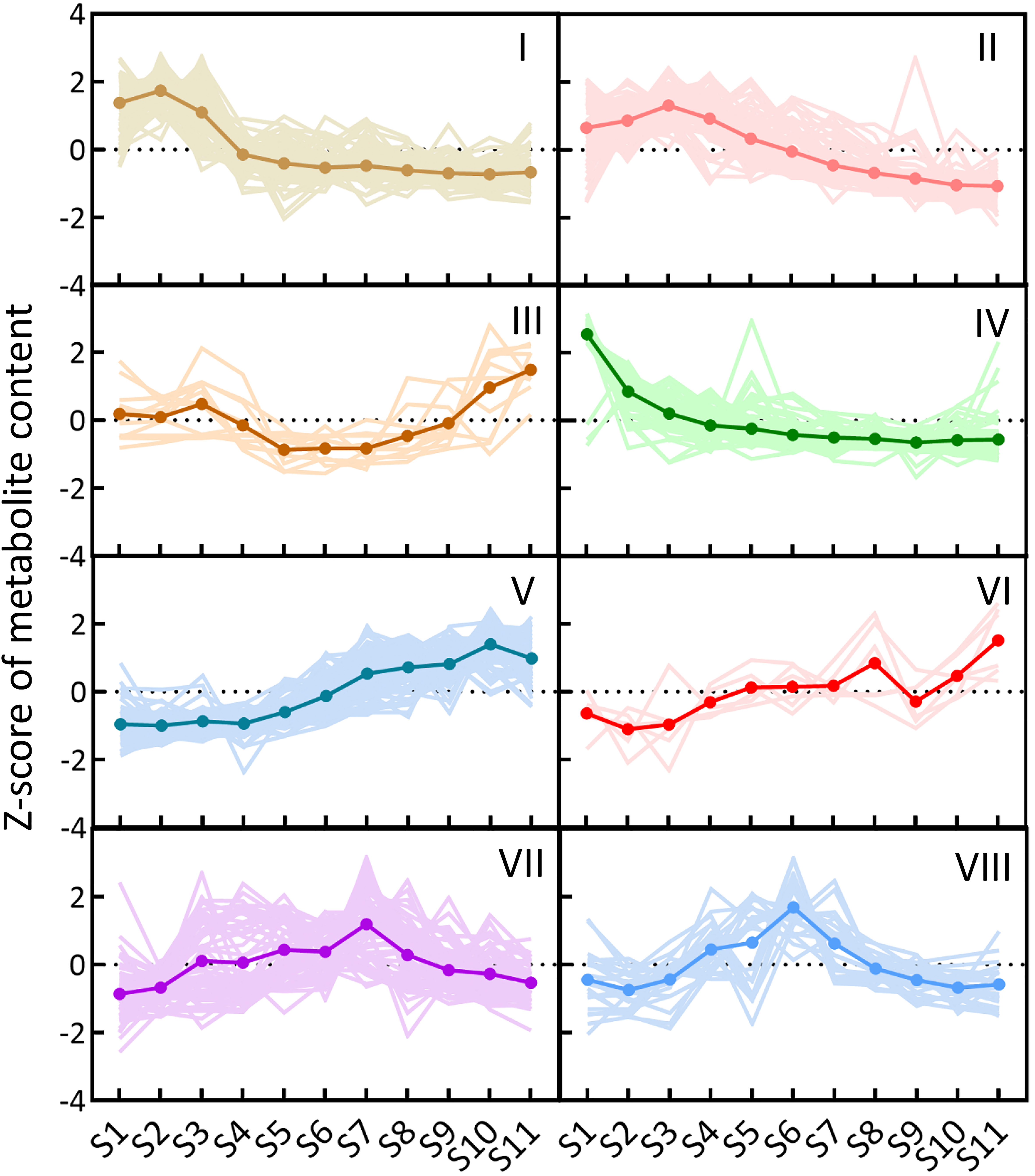


**Figure S2.** Clustering analysis revealed that the profiling of differentially accumulated metabolites were grouped into eight clusters, I→VIII. S1 to S11 indicate different stages. Z-score standardized values of each metabolite were used for clustering analysis. The y axis depicts the Z-score standardized value of each metabolite across all 11 stages.


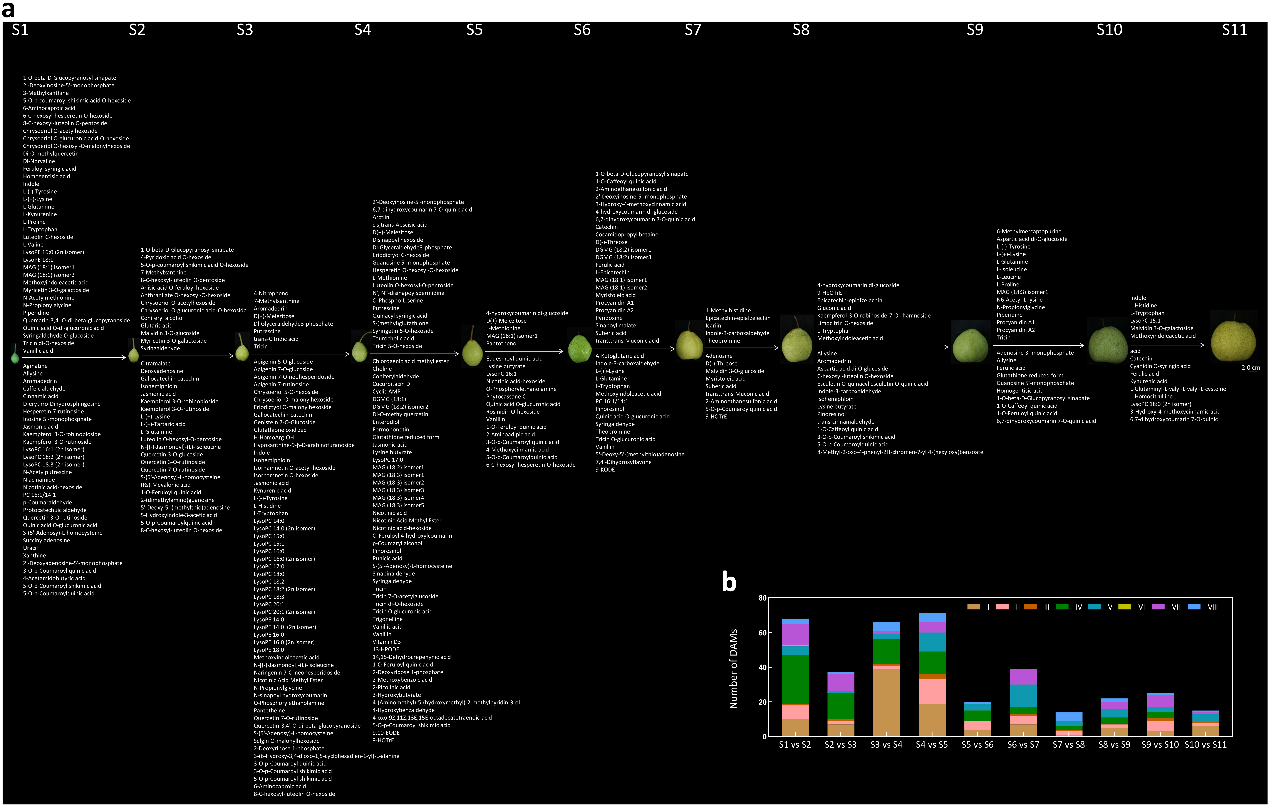


**Figure S3.** Analysis of the metabolites differentially accumulated between two adjacent stages. **a** Identification of the metabolites differentially accumulated between two adjacent stages. The metabolites above arrows are significantly increased in the subsequent stage when compared to the preceding stage. Conversely, the metabolites below arrows are significantly decreased. **b** The number and cluster of the metabolites differentially accumulated between two adjacent stages.


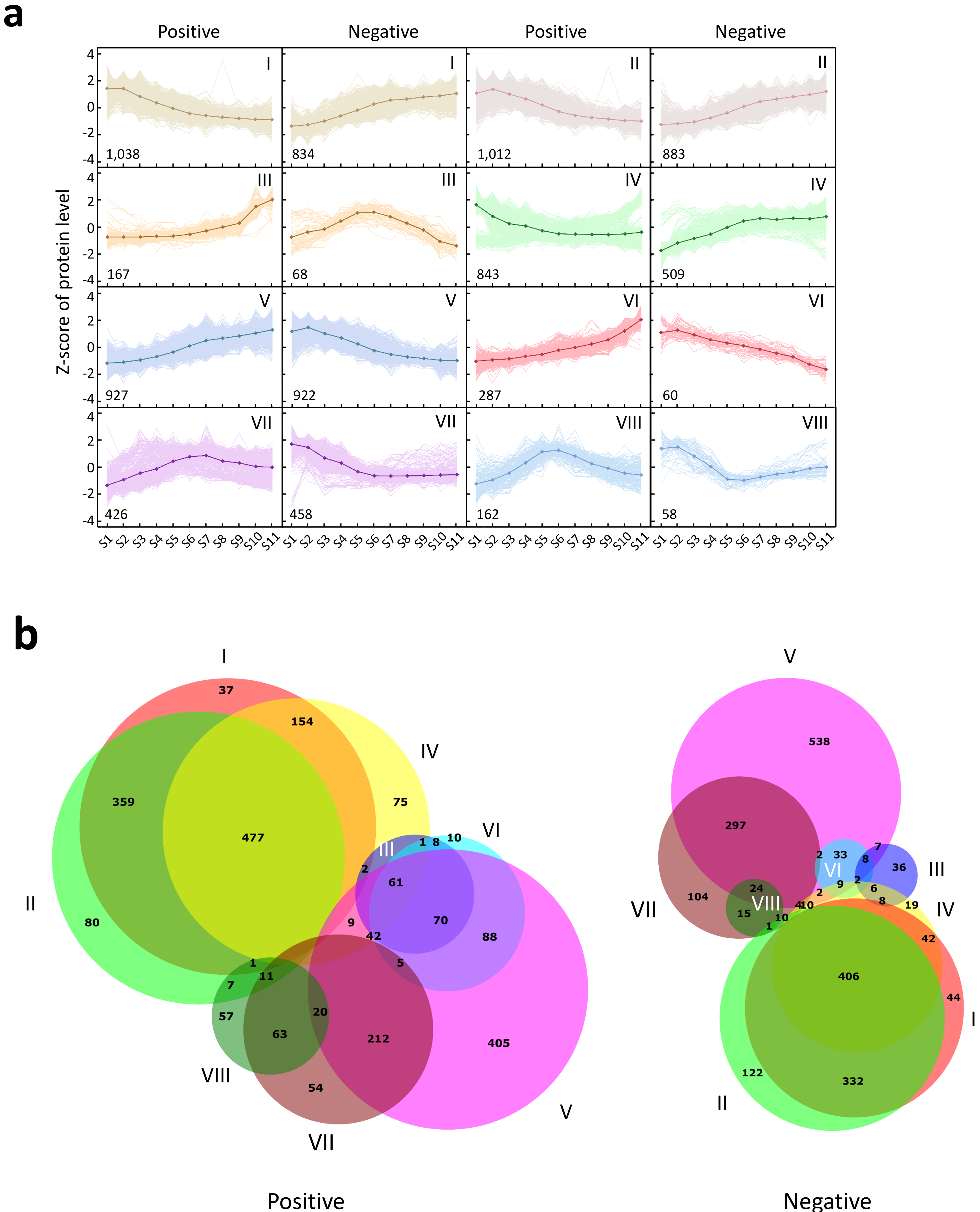


**Figure S4.** Clustering analysis revealed the profiling and number of differentially expressed proteins (DEPs) correlating with the DAMs in eight clusters. **a** The expression patterns of DEPs correlating with the DAMs in eight clusters. The y axis depicts the Z-score standardized value of each protein across all 11 stages (from S1 to S11). The numbers in each box are the number of proteins in each cluster. **b** Two venn diagrams displays the numbers of DEPs among eight clusters. ‘Positive’ represents the positive correlation (Pearson coefficient > 0.85 and false discovery rate < 0.05) of proteins and metabolites in a same cluster, while ‘negative’ represents the negative correlation (Pearson coefficient < -0.85 and false discovery rate < 0.05). I→VIII indicate the different clusters.


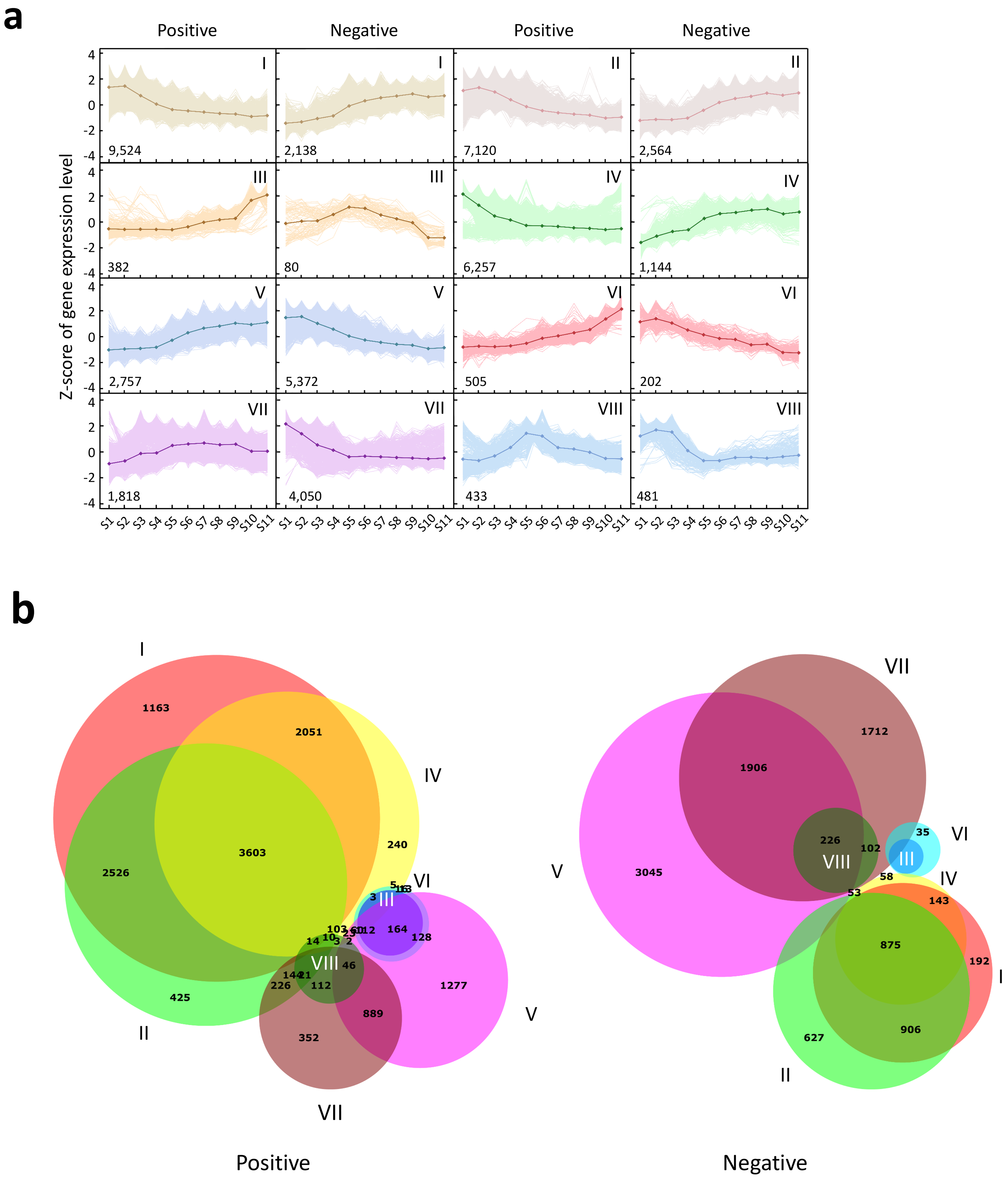


**Figure S5.** Clustering analysis revealed the profiling and number of differentially expressed genes (DEGs) correlating with the DAMs in eight clusters. **a** The expression patterns of DEGs correlating with the DAMs in eight clusters. The y axis depicts the Z-score standardized value of each protein across all 11 stages (from S1 to S11). The numbers in each box are the number of proteins in each cluster. **b** Two venn diagrams displays the numbers of DEGs among eight clusters. ‘Positive’ represents the positive correlation (Pearson coefficient > 0.85 and false discovery rate < 0.05) of genes and metabolites in a same cluster, while ‘negative’ represents the negative correlation (Pearson coefficient < -0.85 and false discovery rate < 0.05). I→VIII indicate the different clusters.


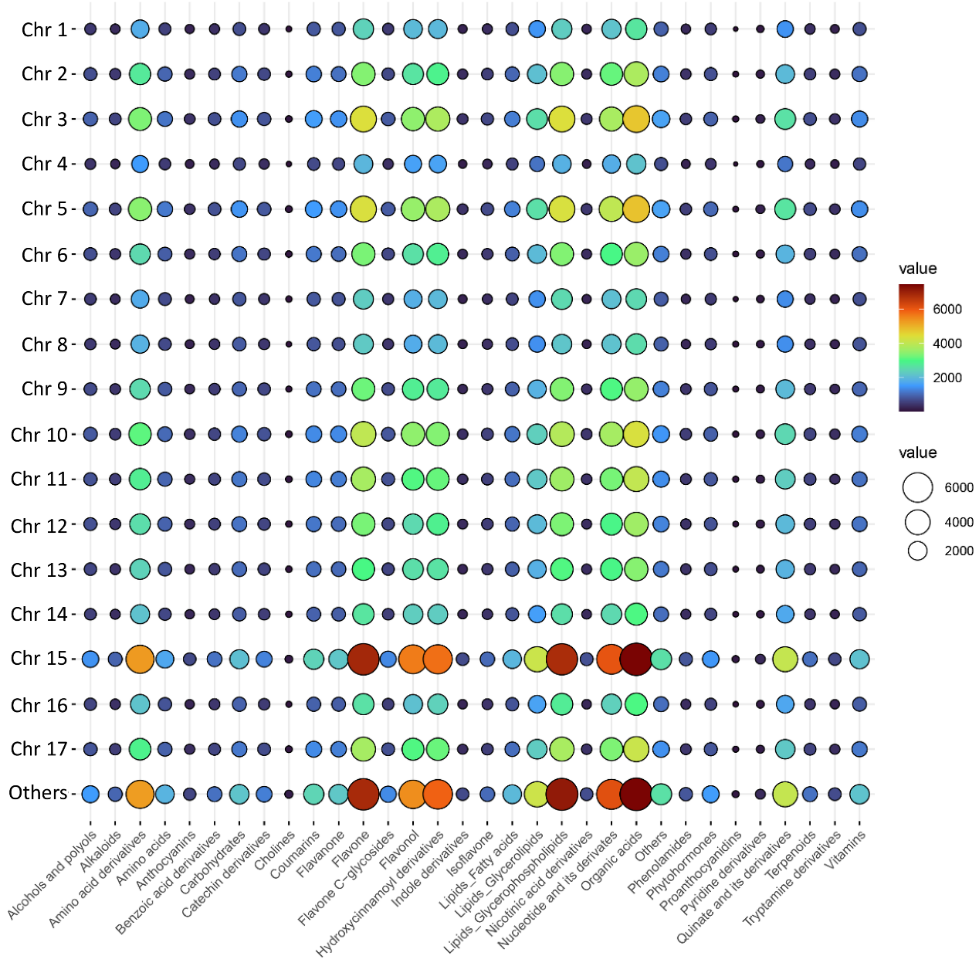


**Figure S6.** The number of DEGs correlated to the DAMs in each metabolic class were counted in each chromosome. Others contained all scaffolds that were unanchored into the 17 chromosomes (Chr 1ꟷ17). ‘Value’ represents the number of genes. The number of genes is positively correlated with the increasing size of the circles and the transition from blue to red color.


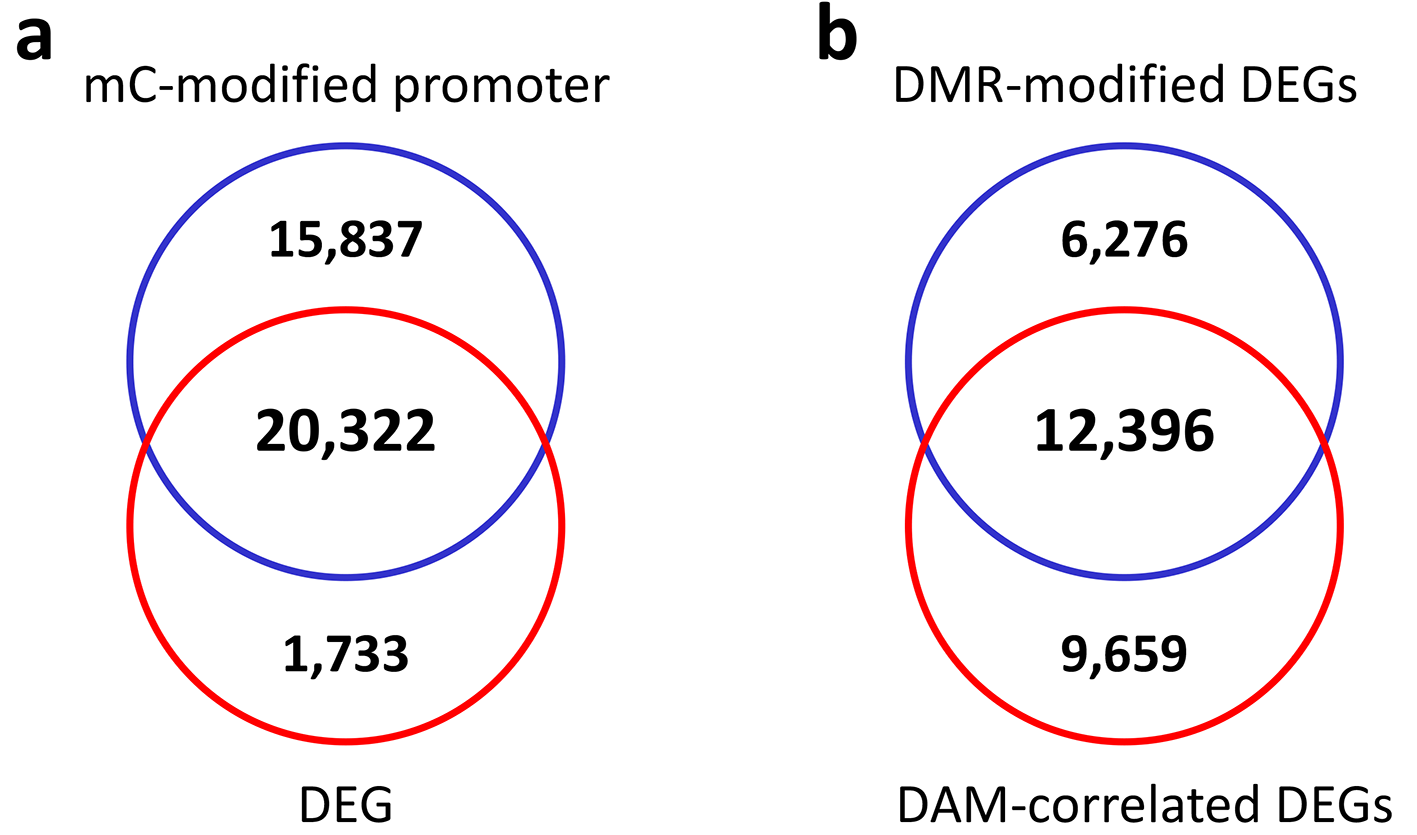


**Figure S7.** Integrative analysis of DNA methylation and DEGs. **a** Identification of the DEGs modified by DNA methylation in the promoters. **b** Identification of the DEGs that are correlated with DAMs and are also modified by DMRs.


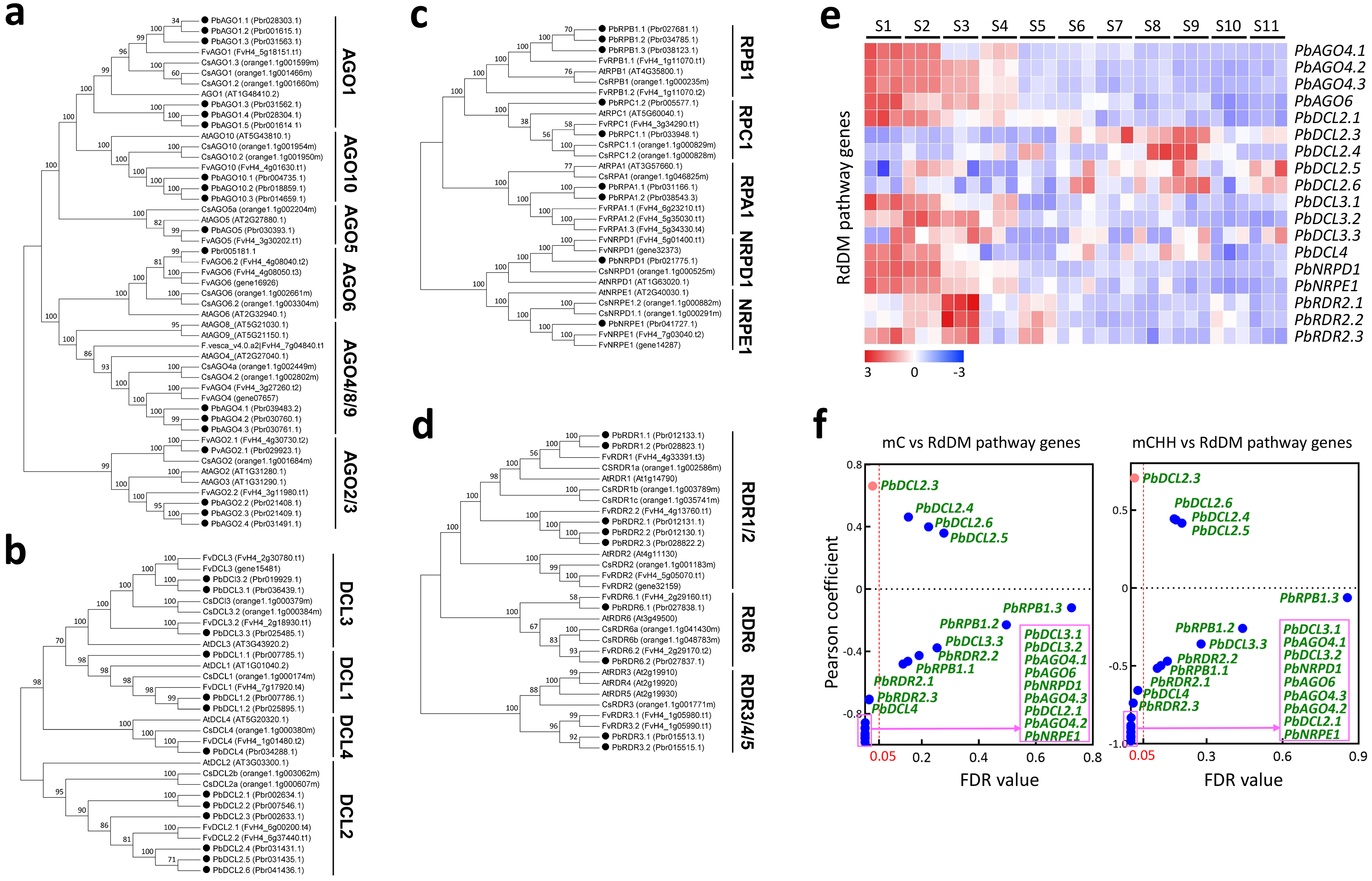


**Figure S8.** Identification and expression analysis of RdDM pathway genes in the fruit flesh. **a** Phylogenetic analysis classified the members of *AGO* gene family into six groups, AGO1, AGO10, AGO5, AGO6, AGO4/8/9, and AGO2/3. **b** Phylogenetic analysis classified the members of *DCL* gene family into four groups, DCL1, DCL2, DCL3, and DCL4. **c** Phylogenetic analysis classified the components of POL IV and V into four groups, RPB1, RPC2, NRPD1, and NRPE1. **d** Phylogenetic analysis classified the members of *RDR* gene family into three groups, RDR1/2, RDR3/4/5, and RDR6. The genes in each phylogenetic tree derive from pear, strawberry, orange, and Arabidopsis genomes. **e** Expression patterns of RdDM pathway genes in the fruit flesh at all 11 stages (S1ꟷS11). Z-score standardized values of each DEG across all 11 stages were used for clustering analysis. The color bar indicates the increasing expression levels of gene from blue to red. **f** Correlation analysis of RdDM pathway genes with C (left panel) and CHH (right panel) methylations. The dotted line with red color represents the false discovery rate value at 0.05.


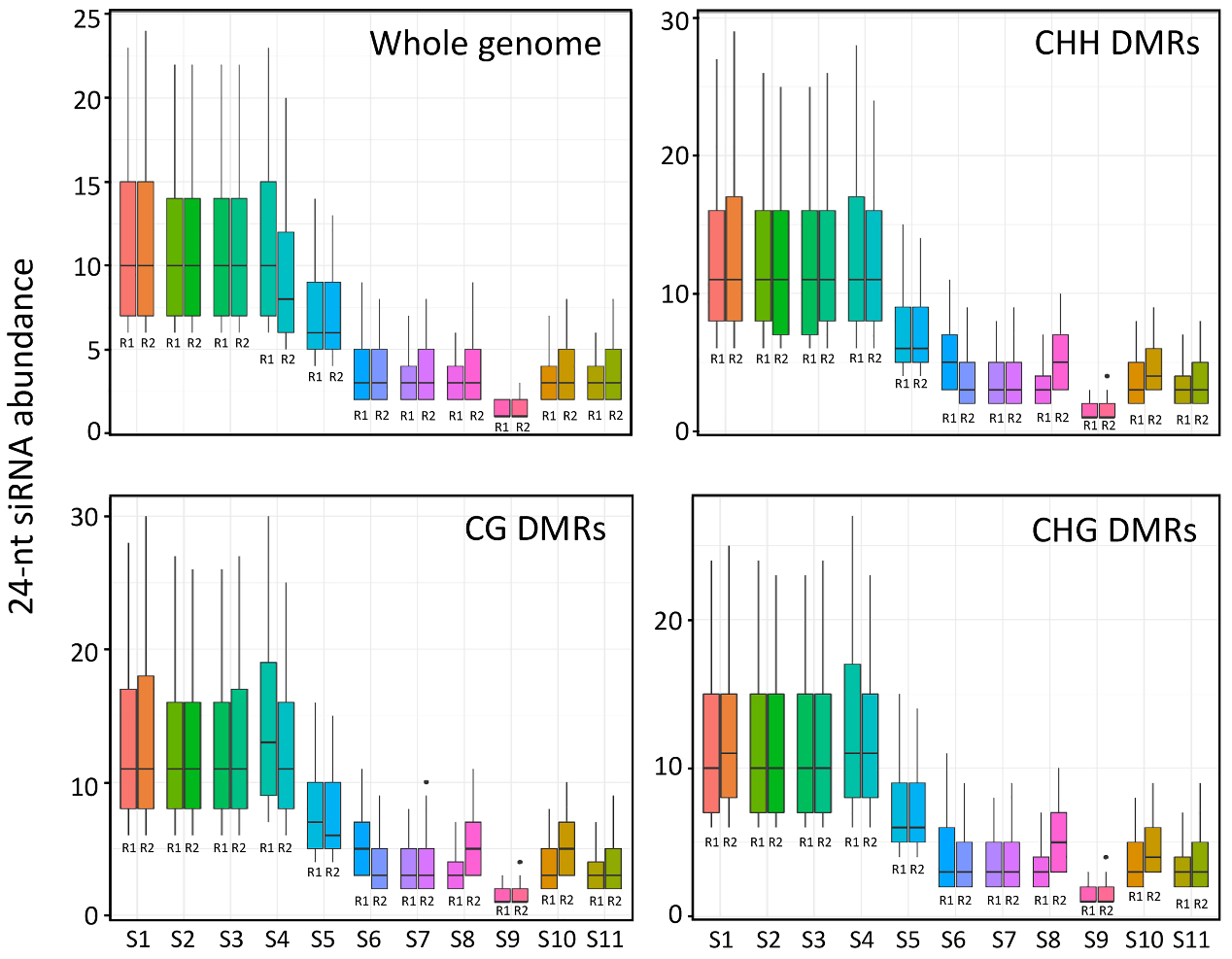


**Figure S9.** Boxplots showing 24-nt small interfering RNA (siRNA) enrichment at whole genome and DMRs of all three contexts. S1 to S11 indicate different stages. R1 and R2 represent the two replicates.


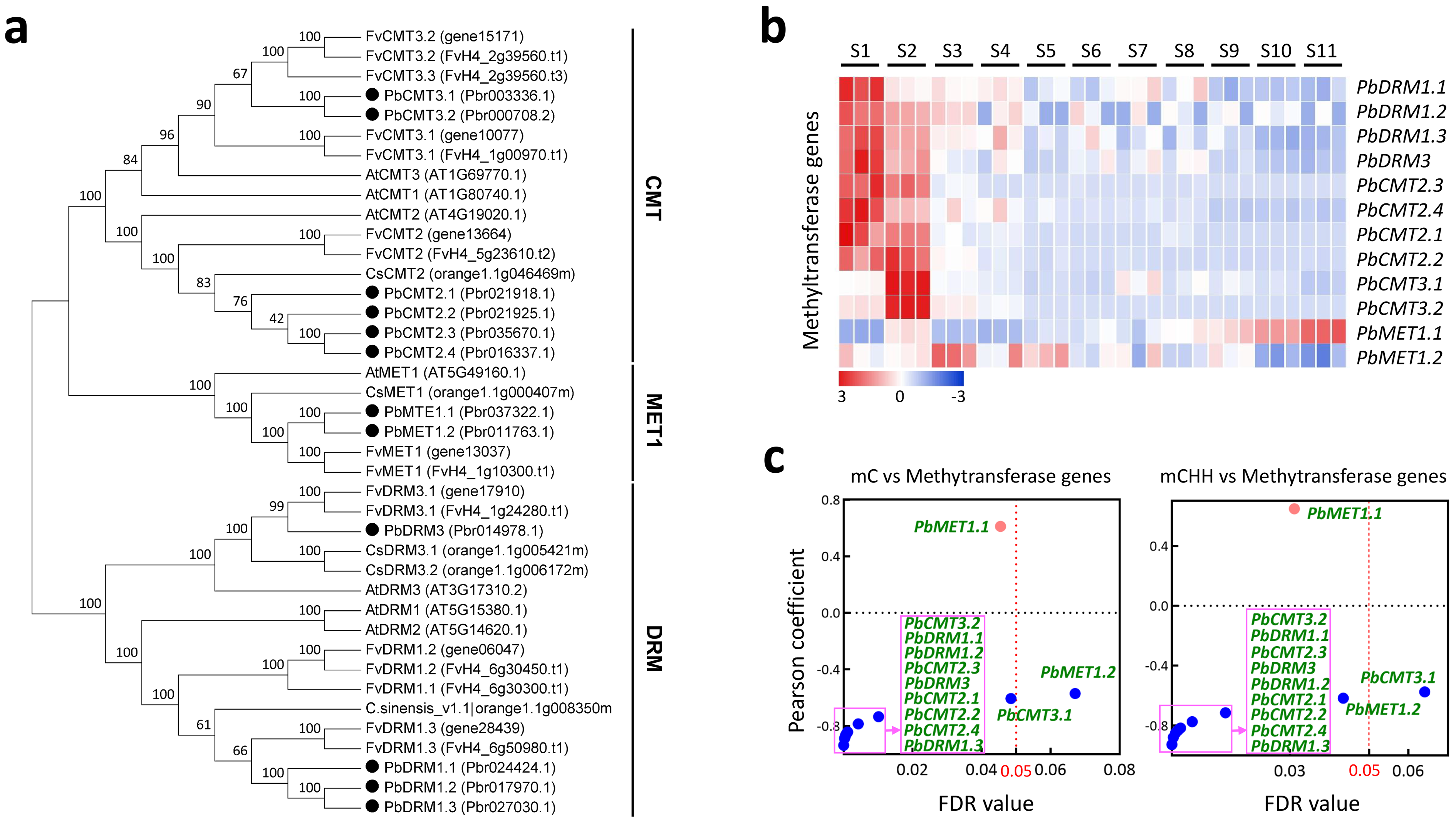


**Figure S10.** Identification and expression analysis of DNA methyltransferase genes in the fruit flesh. **a** Phylogenetic analysis classified the DNA methyltransferase genes into three groups, CMT, MET1, and DRM. The genes in phylogenetic tree derive from pear, strawberry, orange, and Arabidopsis genomes. **b** Expression patterns of DNA methyltransferase genes in the fruit flesh at all 11 stages (S1ꟷS11). Z-score standardized values of each DEG across all 11 stages were used for clustering analysis. The color bar indicates the increasing expression levels of gene from blue to red. **c** Correlation analysis of DNA methyltransferase genes with C (left panel) and CHH (right panel) methylations. The dotted line with red color represents the false discovery rate value at 0.05.


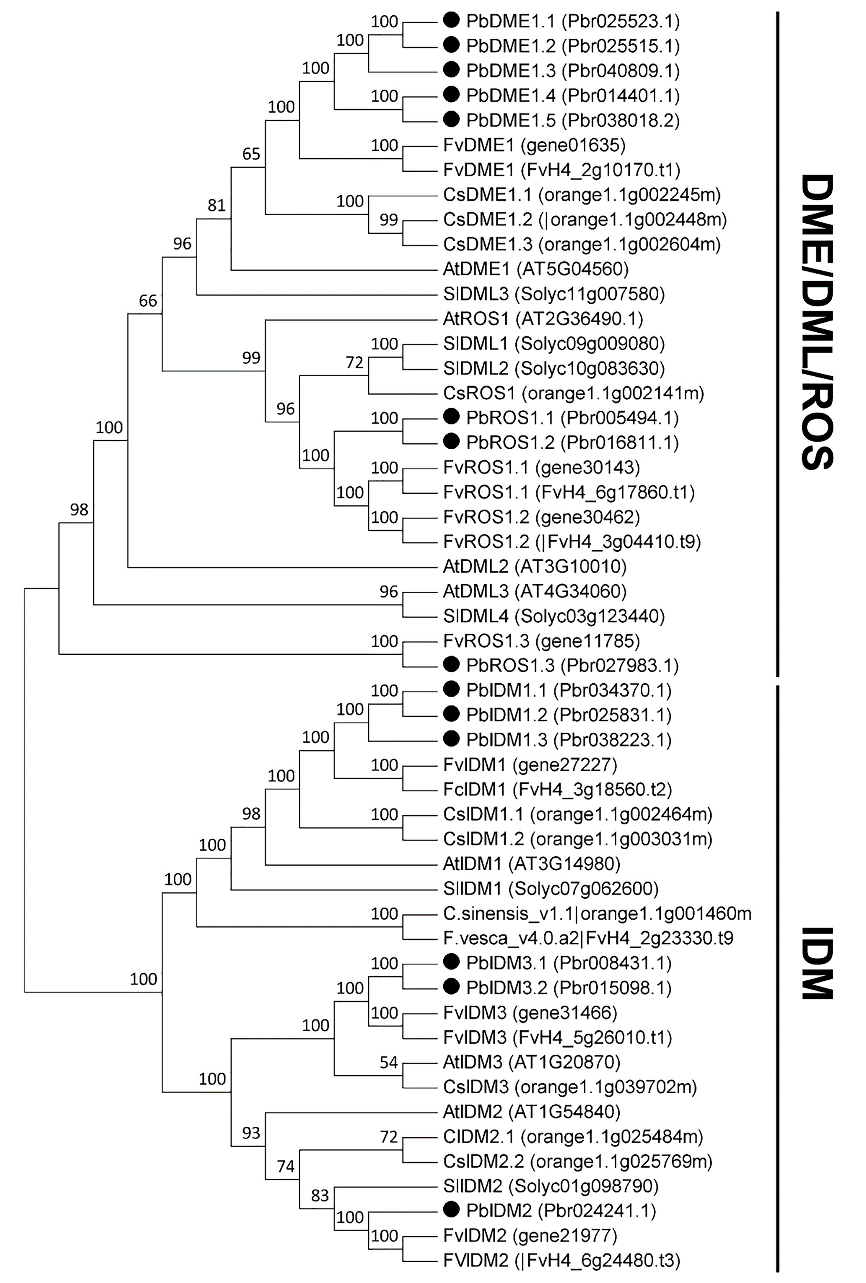


**Figure S11.** Identification of DNA demethylase genes in pear. Phylogenetic analysis classified the DNA demethylase genes into two groups, DME/DML/ROS and IDM. The genes in phylogenetic tree derive from pear, strawberry, orange, and Arabidopsis genomes.


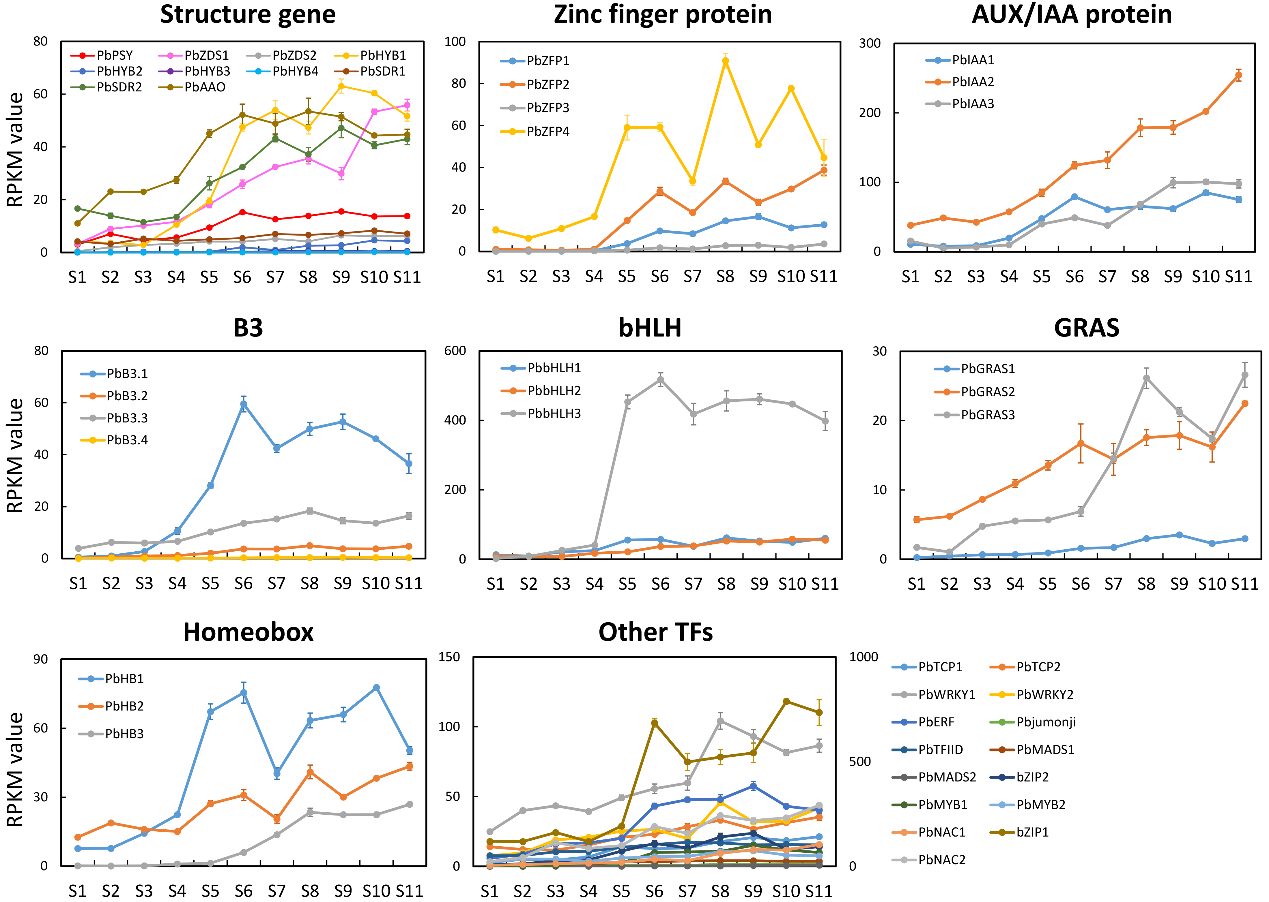


**Figure S12.** Expression pattern of ABA biosynthetic genes and the TFs positively correlated with ABA production. S1 to S11 indicate different stages.


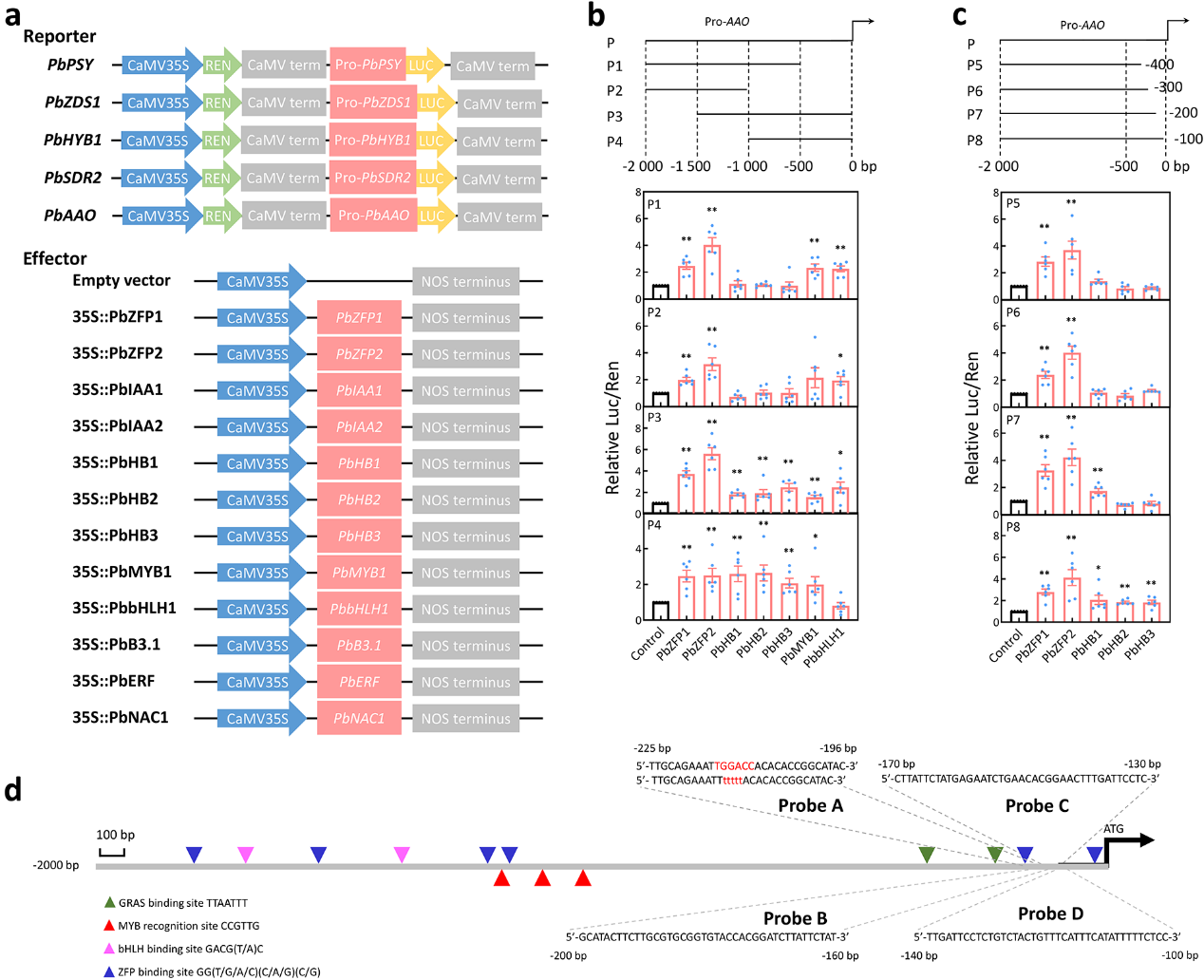


**Figure S13.** Identification of the possible binding regions of multiple TFs in the *PbAAO* promoter. **a** The constructed reporters and effectors. **b** The *PbAAO* promoter was divided into four fragments (P1 to P4; top panel) and was then used for dual-luciferase assay (bottom panel) to determine the binding regions of PbZFP1, PbZFP2, PbHB1, PbHB2, PbHB3, PbMYB1, and PbbHLH1 in the *PbAAO* promoter. **c** The *PbAAO* promoter was further divided into four additional fragments (P5 to P8; top panel) and was then used for dual-luciferase assay (bottom panel) to narrow the binding regions of PbZFP1, PbZFP2, PbHB1, PbHB2, and PbHB3 in the *PbAAO* promoter. Analysis of variance were calculated by Student’s *t*-test. Single and double asterisks stand for the level of significance at *P*-value < 0.05 and < 0.01, respectively. **d** Prediction of *cis*-elements of GRAS, MYB, bHLH, and ZFP TFs. Probes A to D are selected for EMSA.


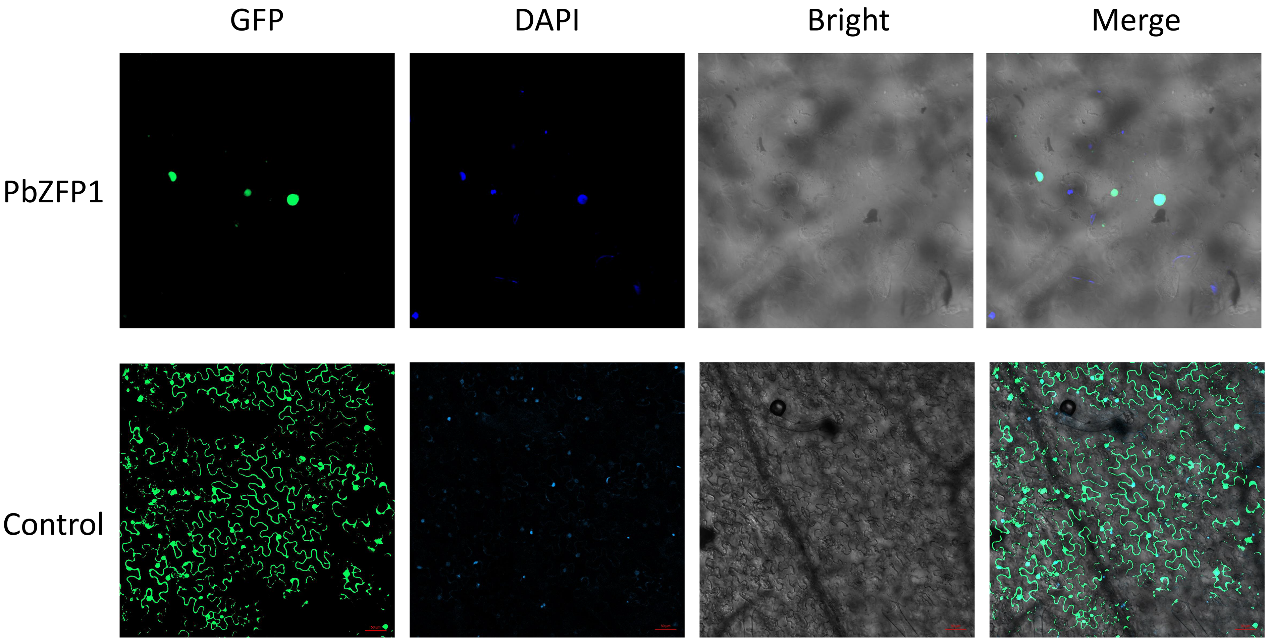


**Figure S14.** Subcellular localization of PbZFP1-GFP fusion protein. PbZFP1 represents the PbZFP1-GFP fusion protein, while control represents the GFP protein.


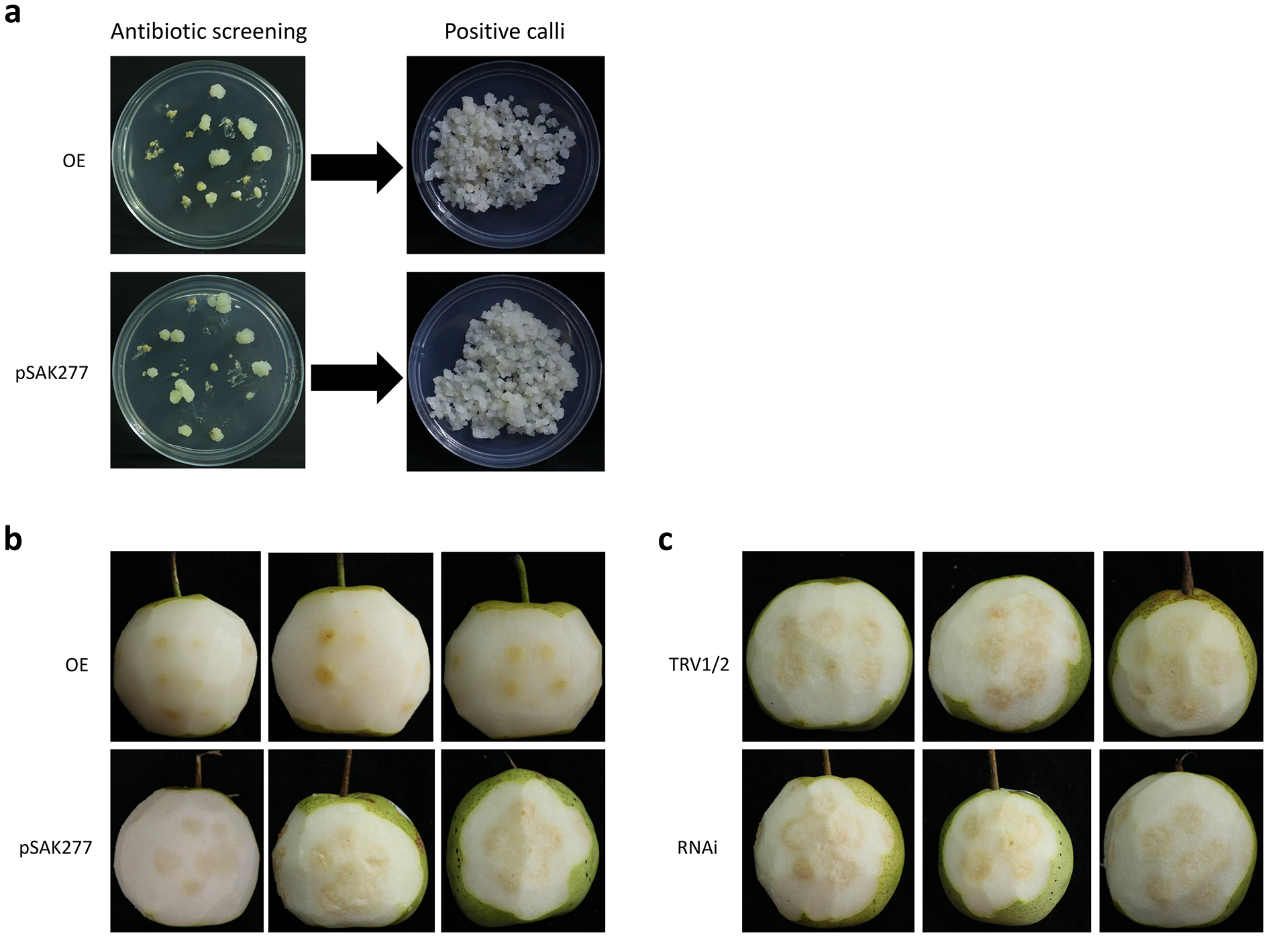


**Figure S15.** Pear transformation of *PbZFP1* in pear flesh callus and fruit flesh. **a** Stable over-expression of PbZFP1 (OE) in flesh callus. **b** Transient over-expression of PbZFP1 (OE) in fruit flesh. pSAK277 is the empty vector and used for the control of OE. **c** Virus-induced gene silencing of PbZFP1 (RNAi) in fruit flesh. TRV1/2 represents the empty vectors of pTRV1 and pTR2 and was used as the control of RNAi.
