# Supplementary figures and images for: Multi-omics provide insights into the regulation of DNA methylation in pear fruit metabolism
Source: Genome Biol. 2024 Mar 14;25:70. doi: 10.1186/s13059-024-03200-2 (PMC10938805; doi:10.1186/s13059-024-03200-2)

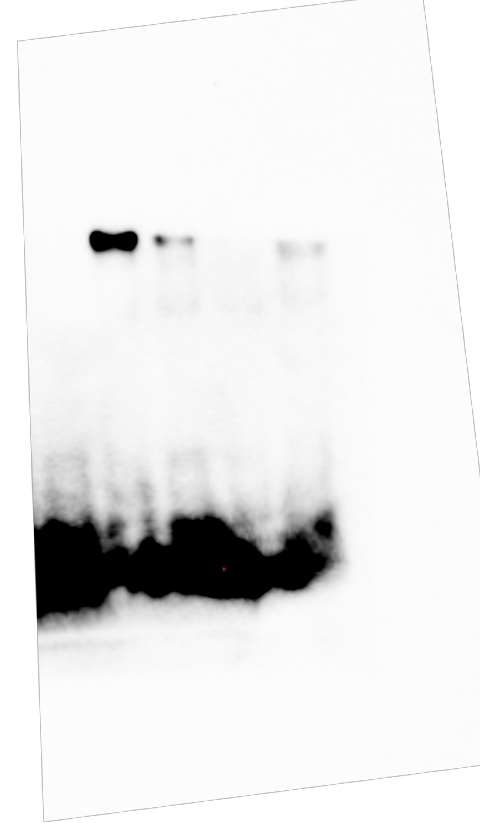

Supplement: Supplementary file 5 — Additional file 5. The uncropped picture for the EMSA image for ZFP1 in Fig. 6c. [file 13059_2024_3200_MOESM5_ESM.tif]

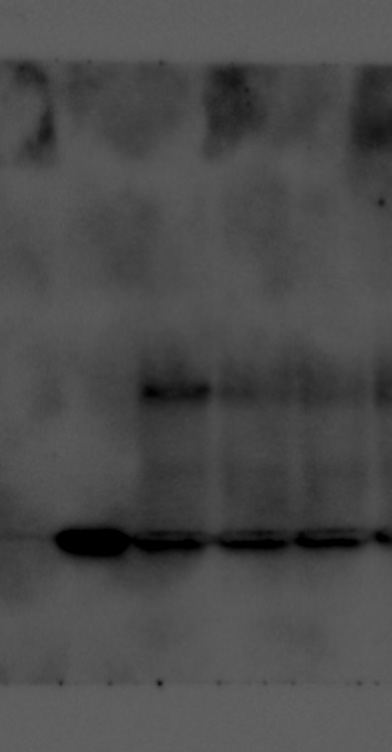

Supplement: Supplementary file 6 — Additional file 6. The uncropped picture for the EMSA image for HB1 in Fig. 6c. [file 13059_2024_3200_MOESM6_ESM.jpg]

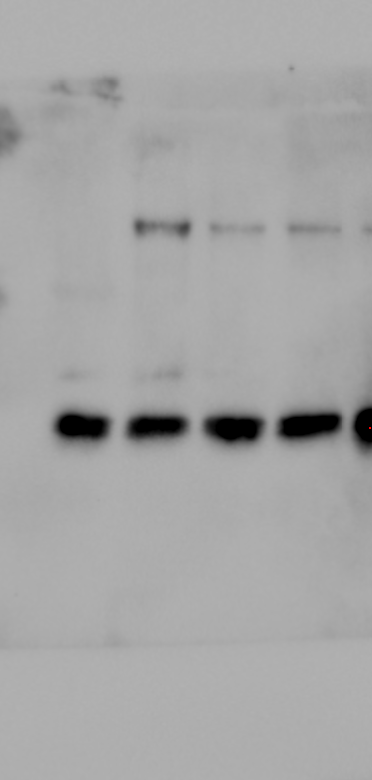

Supplement: Supplementary file 7 — Additional file 7. The uncropped picture for the EMSA image for HB2 in Fig. 6c. [file 13059_2024_3200_MOESM7_ESM.tif]

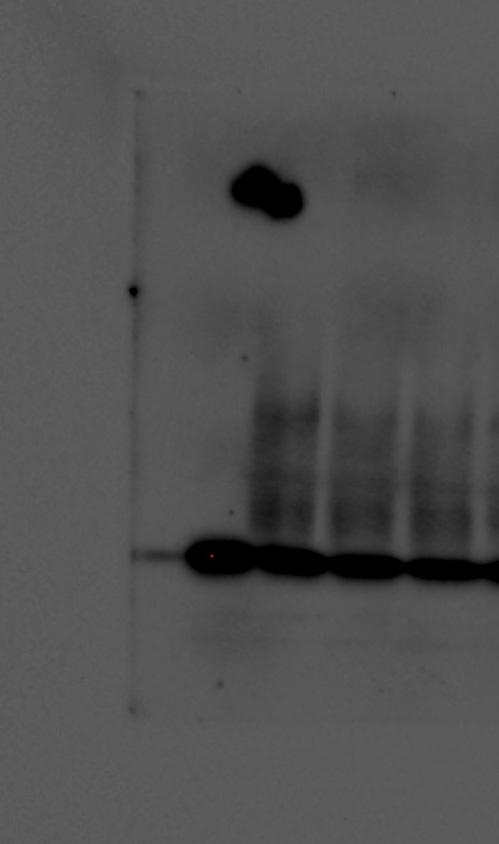

Supplement: Supplementary file 8 — Additional file 8. The uncropped picture for the EMSA image for HB3 in Fig. 6c. [file 13059_2024_3200_MOESM8_ESM.jpg]
