# Supplementary material for: Multi-omics provide insights into the regulation of DNA methylation in pear fruit metabolism
Source: Genome Biol. 2024 Mar 14;25:70. doi: 10.1186/s13059-024-03200-2 (PMC10938805; doi:10.1186/s13059-024-03200-2)
Supplement: Supplementary file 9 — Additional file 9. Review history. [file 13059_2024_3200_MOESM9_ESM.docx]

Review History

**First round of review**

**Reviewer 1**

**Were you able to assess all statistics in the manuscript, including the appropriateness of statistical tests used?**

Yes, and I have assessed the statistics in my report.

**Comments to author:**

1. P20 Line 13-16, Did the fruits come from a single or different tree?

2. P20 Line 16-18, the fruits were peeled and used for further analysis. It seems that the fruit peel was discarded and only the flesh was used for multi-omics analysis. The fruit flesh and peel need to be measured at the same time, individually, or at least measured together. Also, other omics methods need to be conducted in flesh and peel. Otherwise, it can't represent the regulation of metabolic regulatory networks of pear fruit development.

3. P20 Line 26-28, 'An amount of 0.1 g of dry powder was extracted and used for measurements…'. The water percentage of fruit will be different towards fruit ripening, the metabolite content in dry powder of the same amount can't reflect the real situation. It's better to normalize the metabolite content to fresh fruit weight.

4. P20 Line 26-28, please specify how the samples were extracted.

5. Additional file 1, I feel it important that the metabolomics data is reported following recent updates to reporting standards published last year in Nature Methods.

6. Figure 1, the fruit color in stage 6 seems to be different compared to S5 and S7, please explain it.

7. P6 Line 1-2, '…including L-Alanine, L-Phenylalanine, L-Proline, L-Valine in cluster VII, may be associated with fruit enlargement.' Is there any evidence that these amino acids will affect fruit enlargement?

8. The physical data like fruit weight, pericarp and flesh color, texture, ripening index, stone cells, soluble sugar, ethylene release et al. should be included in the manuscript. Because these indexes will be very different from samples of different years or even from different orchards. Additionally, when the authors compare the metabolites or other omics data that will largely depend on the exact physical data.

9. P13 Line 33-44, The authors didn't measure the methylation level of pericarp during pear fruit development. How can the early developmental fruits with green pericarp correlate with the low level of DNA methylation?

10. Figure 6. More experiments like Y1H, sub-localization, etc. need to be conducted to testify to the transcriptional regulation of a new gene. In addition, how about other selected transcription factors? Do they have coregulation effects or they are not involved?

11. At last, I should say I see a lot of data in the manuscript but it has not been fully analyzed. The combination and comparison analysis among different omics were not enough, more methods or software need to be used to further investigate the correlation and potential regulation of different metabolism pathways.

**Reviewer 2**

**Were you able to assess all statistics in the manuscript, including the appropriateness of statistical tests used?**

Yes, and I have assessed the statistics in my report.

**Comments to author:**

On P10 the authors describe their gene-metabolite database. They note a collection of functionally known genes whose expression correlates with metabolites associated with known function. They cite these correlations as evidence of the robustness of their correlation analyses. How many known gene-metabolite correlations did not support their hypothesis. While the results shown are encouraging, it would be much more relevant to take say 100 known gene-metabolite correlations and ask how many are recovered in their analysis. This or something similar will give a quantitative measure of robustness of their correlations and give similar weight to the most interesting correlations (those where the relationship is currently unknown).

The 5az treatment of pear callus demonstrated reduced methylation when looking at 2 genes. This is interesting but not very convincing. Given the enormous amount of whole genome bisulfite done in this work why not include whole genome analysis so all 948 DEGs could be similarly analyzed? The jump from analysis of two genes to inference for the meaning of methylation changes in 18,672 genes differentially expressed in pear fruit is not justified. Doing the WGBS on the callus would help but I am not sure would be very convincing in the context of the meaning in developing pear genomes. Can 5az treatments be done in whole pear fruit as was done for the ABA analysis that follows? This would then need to be followed by WGBS as the assessment of a few genes of interest does not allow the broad statements made. This would be a more accurate and direct way of assessing the relevance of methylation changes on fruit development.

The analysis and discussion of RdDM genes and correlation with small RNAs and methylation is not convincing. Many genes are involved and with differences in expression of family members. Simple correlation analyses does not allow the associations the authors attempt to imply.

The treatment of fruit tissues with 5az followed by assessment of said tissue for expression is helpful but again, why not WGBS rather than just a few target genes. This analysis as performed is relevant to the genes in question but WGBS could much better support he larger assertions made regarding whole genome methylation effects and broader influence on expression within the genome of the developing pear.

The identification of ZFP1 TF as a regulator of ABA and ripening in pear is the best supported component of the presentation with solid data indicating target promoters, metabolic outcomes and changes in ripening-related phenotypes. In earlier work in tomato it was shown that an important ripening TF actually bound to the regions changing in methylation at the onset of ripening. With a key TF identified and a role in regulating a key hormone this begs the question of whether or not similar phenomena are occurring in pear which as the authors note has a distinct fruit tissue origin as compared to tomato.

In short the authors have collected an impressive data set on multiple omics parameters during pear ripening. At present the correlation analyses are over-interpreted and need to be better justified. A novel ripening transcription factor has been identified but has been presented in a preliminary manner compared to what has been shown in other systems. Addressing these concerns will greatly enhance the value and impact of this work.

**Authors Response**

**Point-by-point responses to the reviewers’ comments:**

**Reviewer #1:**

1. P20 Line 13-16, Did the fruits come from a single or different tree?

*Response: The developing and ripening fruits were collected from a single 12-year-old pear tree. For each collection, three or four fruits were picked from each of east, south, west, and north.*

2. P20 Line 16-18, the fruits were peeled and used for further analysis. It seems that the fruit peel was discarded and only the flesh was used for multi-omics analysis. The fruit flesh and peel need to be measured at the same time, individually, or at least measured together. Also, other omics methods need to be conducted in flesh and peel. Otherwise, it can't represent the regulation of metabolic regulatory networks of pear fruit development.

*Response: Thanks for your suggestion. Indeed, we focused on the multi-omics analysis of flesh, because the flavor of pear fruit was largely contributed by the flesh part. To clarify the tissue we used in the analysis, we have changed the ‘fruit development’ to ‘flesh development’.*

3. P20 Line 26-28, 'An amount of 0.1 g of dry powder was extracted and used for measurements…'. The water percentage of fruit will be different towards fruit ripening, the metabolite content in dry powder of the same amount can't reflect the real situation. It's better to normalize the metabolite content to fresh fruit weight.

*Response: Thanks for your reminding. The description of sample extraction was incomplete. The metabolite content had been normalized in this study. Please see details in lines 10-23 on page 21.*

4. P20 Line 26-28, please specify how the samples were extracted.

*Response: Thanks for your suggestion. We have supplemented it. Please see details in lines 10-23 on page 21.*

5. Additional file 1, I feel it important that the metabolomics data is reported following recent updates to reporting standards published last year in Nature Methods.

*Response: Thanks for your suggestion. However, the method used in this study is different from the standards published in Nature Methods. In this study, a liquid chromatography-electrospray ionization-tandem mass spectrometry system was used for the relative quantification of the metabolites, and the broadly targeted metabolites were identified by using authentic standards. This method has been used in many crops, including rice (Chen et al., 2013), cereals (Chen et al., 2015), and tomato (Zhu et al., 2018). In the report in Nature Methods, the introduced methods focus on how to identify the metabolites.*

*Reference:*

*Zhu G, Wang S, Huang Z, Zhang S, Liao Q, Zhang C, Lin T, Qin M, Peng M, Yang C, Cao X, Han X, Wang X, van der Knaap E, Zhang Z, Cui X, Klee H, Fernie AR, Luo J, Huang S (2018) Rewiring of the Fruit Metabolome in Tomato Breeding. Cell 172:249-261.*

*Chen W, Gong L, Guo Z, Wang W, Zhang H, Liu X, Yu S, Xiong L, Luo J. (2013) A novel integrated method for large-scale detection, identification, and quantification of widely targeted metabolites: application in the study of rice metabolomics. Molecular Plants. 6: 1769–1780.*

*Chen W, Wang W, Peng M, Gong L, Gao Y, Wan J, Wang S, Shi L, Zhou B, Li Z, Peng X, Yang C, Qu L, Liu X, Luo J. (2015) Comparative and parallel genome-wide association studies for metabolic and agronomic traits in cereals. Nature Communication. 7: 12767.*

6. Figure 1, the fruit color in stage 6 seems to be different compared to S5 and S7, please explain it.

*Response: We are so sorry for this confusion. The difference resulted from the light intensity during photographing. We have renewed the pictures. Please see details in the revised Figure 1a.*

7. P6 Line 1-2, '…including L-Alanine, L-Phenylalanine, L-Proline, L-Valine in cluster VII, may be associated with fruit enlargement.' Is there any evidence that these amino acids will affect fruit enlargement?

*Response: Yes, in pomegranate fruits, exogenous treatments of* ***proline*** *could increase fruit* *weight (Sayed et al. 2014). In grape berries, exogenous treatment of GA3 (Gibberellic acid) could increase the* ***valine*** *content (330%) and fruit weight (Weaver et al., 1969). It is not reported that* ***alanine*** *and* ***phenylalanine*** *are directly involved in fruit enlargement, but both are involved in phenylalanine metabolism that is associated with tricarboxylic acid cycle. Therefore, we proposed that these amino acids may be associated with fruit enlargement.*

*Reference:*

*Weaver RJ, Shindy W, Kliewer M. 1969. Growth regulator induced movement of photosynthetic products into fruits of `Black Corinth' grapes. Plant Physiology, 44: 183-188.*

*Sayed OMEL, Gammal OHMEL, Salama ASM. 2014. Effect of proline and tryptophan amino acids on yield and fruit quality of Manfalouty pomegranate variety. Scientia Horticulturae, 169: 1-5.*

8. The physical data like fruit weight, pericarp and flesh color, texture, ripening index, stone cells, soluble sugar, ethylene release et al. should be included in the manuscript. Because these indexes will be very different from samples of different years or even from different orchards. Additionally, when the authors compare the metabolites or other omics data that will largely depend on the exact physical data.

*Response: Thanks for your suggestion. We have supplemented the physical data, including fruit weight, longitudinal diameter, transverse diameter, soluble solids, stone cells, fruit firmness, soluble sugar, and ethylene production. Please see details in Figure S1. Moreover, the description was supplemented in lines 22-38 on page 5.*

9. P13 Line 33-44, The authors didn't measure the methylation level of pericarp during pear fruit development. How can the early developmental fruits with green pericarp correlate with the low level of DNA methylation?

*Response: Thanks for your suggestion. We have removed the related conclusion from the main text. Please see details in lines 12-13 on page 14.*

10. Figure 6. More experiments like Y1H, sub-localization, etc. need to be conducted to testify to the transcriptional regulation of a new gene. In addition, how about other selected transcription factors? Do they have coregulation effects or they are not involved?

*Response: Thanks for your suggestion. We have supplemented Y1H and sub-localization. Please see details in Figure 6b and S14. Moreover, transient overexpression and silencing of several TFs were performed in pear flesh. The result showed that they were involved in ABA biosynthesis. However, only PbZFP1 was successfully transformed into fruit callus. Thus, we are not sure whether the other TFs must be involved in ABA biosynthesis. In future, more experiments should be performed to determine it.*

11. At last, I should say I see a lot of data in the manuscript but it has not been fully analyzed. The combination and comparison analysis among different omics were not enough, more methods or software need to be used to further investigate the correlation and potential regulation of different metabolism pathways.

*Response: Thanks for your suggestion. To investigate the correlation and potential regulation of different metabolism pathway, the gene-metabolite networks were constructed for each cluster and the results were described in the main text. Please see details in lines 2-24 on page 9 and Figure S6.*

**Reviewer #2:**

1. On P10 the authors describe their gene-metabolite database. They note a collection of functionally known genes whose expression correlates with metabolites associated with known function. They cite these correlations as evidence of the robustness of their correlation analyses. How many known gene-metabolite correlations did not support their hypothesis. While the results shown are encouraging, it would be much more relevant to take say 100 known gene-metabolite correlations and ask how many are recovered in their analysis. This or something similar will give a quantitative measure of robustness of their correlations and give similar weight to the most interesting correlations (those where the relationship is currently unknown).

*Response: Thanks for your suggestion. For each metabolite, the metabolism pathway were investigated based on the KEGG database. A total of 220 metabolites could be detected in the* *kyoto encyclopedia of genes and genomes (KEGG) database, but the pathway genes of only 101 metabolites were surveyed in the transcriptome data of pear flesh. Of the 101 metabolites, 63 positively or negatively correlated with their pathway genes. The details are listed in Table S8. However, this coverage is tightly related to the threshold. If the threshold was designated as Pearson coefficient < -0.80 and false discovery rate < 0.05, 71 metabolites were correlated to their pathway genes. If the threshold was designated as Pearson coefficient < -0.75 and false discovery rate < 0.05, 77 metabolites were correlated to their pathway genes. If the threshold was designated as Pearson coefficient < -0.70 and false discovery rate < 0.05, 82 metabolites were correlated to their pathway genes.*

2. The 5az treatment of pear callus demonstrated reduced methylation when looking at 2 genes. This is interesting but not very convincing. Given the enormous amount of whole genome bisulfite done in this work why not include whole genome analysis so all 948 DEGs could be similarly analyzed? The jump from analysis of two genes to inference for the meaning of methylation changes in 18,672 genes differentially expressed in pear fruit is not justified. Doing the WGBS on the callus would help but I am not sure would be very convincing in the context of the meaning in developing pear genomes. Can 5az treatments be done in whole pear fruit as was done for the ABA analysis that follows? This would then need to be followed by WGBS as the assessment of a few genes of interest does not allow the broad statements made. This would be a more accurate and direct way of assessing the relevance of methylation changes on fruit development.

*Response: Thanks for your suggestion. We have conducted the WGBS in fruit calli with the treatment of 5’-Aza to survey the DNA methylation level of 948 DEGs. The result was supplemented in the main text. Please see details in lines 21-28 on page 10 and Table S11.*

3. The analysis and discussion of RdDM genes and correlation with small RNAs and methylation is not convincing. Many genes are involved and with differences in expression of family members. Simple correlation analyses does not allow the associations the authors attempt to imply.

*Response: Thanks for your suggestion. The role of RdDM genes and small RNAs in DNA* *methylation has been clarified in Arabidopsis, tomato, strawberry, and sweet orange (Lang et al., 2017; Cheng et al., 2018; Zhang et al., 2018; Huang et al., 2019). In this study, to survey the reason why the DNA methylation level was increased during pear flesh development, we also detected the expression pattern of all genes involved in RdDM pathway and the enrichment of small RNAs. We agree with the reviewer that gene expression pattern of related pathway was not sufficient to support our conclusion. However, our conclusion was also supported by the data of the enrichment of small RNAs, which could be used as a direct measurement of RdDM activity. We found that the expression patterns of the genes involved in RdDM pathway and the enrichment of small RNAs are not consistent with the increased DNA methylation level. However, the expression patterns of the genes involved in DNA demethylation are consistent with the increased DNA methylation level. Therefore, we made the conclusion that the increased DNA methylation during pear flesh development may be contributed by the decreased expression of the genes involved in DNA demethylation.*

*We have removed the ‘association’ from the main text to make the conclusion more reasonable. If you have any better suggestion, please do not hesitate to tell us, we will revised it immediately.*

*References:*

*Lang Z, Wang Y, Tang K, Tang D, Datsenka T, Cheng J, Zhang Y, Handa AK, Zhu JK (2017) Critical roles of DNA demethylation in the activation of ripening-induced genes and inhibition of ripening-repressed genes in tomato fruit. Proceedings of the National Academy of Sciences of the United States of America 114: E4511-E4519*

*Cheng JF, Niu QF, Zhang B, Chen KS, Yang RH, Zhu JK, Zhang YJ, Lang ZB. (2018) Downregulation of RdDM during strawberry fruit ripening. Genome Biol. 19: 212.*

*Zhang H, Lang Z, Zhu JK. (2018) Dynamics and function of DNA methylation in plants. Nat Rev Mol Cell Biol. 19: 489-506.*

*Huang H, Liu RE, Niu QF, Tang K, Zhang B, Zhang H, Chen KS, Zhu JK, Lang ZB (2019) Global increase in DNA methylation during orange fruit development and ripening. Proceedings of the National Academy of Sciences of the United States of America 116:1430-1436*

4. The treatment of fruit tissues with 5az followed by assessment of said tissue for expression is helpful but again, why not WGBS rather than just a few target genes. This analysis as performed is relevant to the genes in question but WGBS could much better support he larger assertions made regarding whole genome methylation effects and broader influence on expression within the genome of the developing pear.

*Response: Thanks for your suggestion. We have conducted the WGBS in fruit flesh and pericarp with the treatment of 5’-Aza. The result was supplemented in the main text. Please see details in lines 2-19 on page 14 and Table S11.*

5. The identification of ZFP1 TF as a regulator of ABA and ripening in pear is the best supported component of the presentation with solid data indicating target promoters, metabolic outcomes and changes in ripening-related phenotypes. In earlier work in tomato it was shown that an important ripening TF actually bound to the regions changing in methylation at the onset of ripening. With a key TF identified and a role in regulating a key hormone this begs the question of whether or not similar phenomena are occurring in pear which as the authors note has a distinct fruit tissue origin as compared to tomato.

*Response: Thanks for your suggestion. We have surveyed the methylation level of ZFP1-binding elements, but cannot find the changes in DNA methylation level in the promoter of PbAAO. However, in the promoters of some other DEGs, such as PbPSY, PbZDS1, and PbSDR2, the DNA methylation level of ZFP1-binding elements was changed during pear flesh development. We will pay more attention to this suggestion in following experiments.*

6. In short the authors have collected an impressive data set on multiple omics parameters during pear ripening. At present the correlation analyses are over-interpreted and need to be better justified. A novel ripening transcription factor has been identified but has been presented in a preliminary manner compared to what has been shown in other systems. Addressing these concerns will greatly enhance the value and impact of this work.

*Response: Thanks for your appreciation. We have adjusted the correlation analysis in the main text. The binding elements of the novel ripening transcription factor PbZFP1 were identified from the promoters of ABA biosynthetic genes and other DEGs. The changes in cytosine methylation were surveyed based on the DNA methylation levels across all 11 stages. Finally, we found that the cytosine methylation was not detected in the ZFP1-binding elements in the promoter of PbAAO. However, the DNA methylation level of ZFP1-binding elements was changed in the promoters of some other DEGs. Anyway, thanks for your suggestion. We will pay more attention to this suggestion in following experiments.*

**Second round of review**

**Reviewer 1**

The manuscript has been much improved, but the concerns were not fully illustrated. Please see the attached comments.

1. The authors conducted the work using fruit flesh and did not consider the fruit pericarp, which is a lack in this manuscript.

2. Figure 1 and Figure 2, simply show the variation/expression trends of metabolites, genes, and proteins is not a good way to elucidate the data. It makes the readers bored and thinks the article was prepared poorly. The trends data mostly should be exhibited as supporting information. Emphasize the key point of the manuscript, not to let the large data swallow the readers.

2. Additional file 1, please fulfill the detailed information of all the metabolites, and make it easier for other researchers to follow. The Nature Methods paper can be referred to.

3. The profiled protein and small RNA data were not closely related to the manuscript, two aspects need to be considered. Is it due to the un-robust data analysis that the relationship has not been fully understood? Is it still necessary to keep these data? Such big data and not fully analyzed causes a huge amount of redundant information.

**Reviewer 3**

Upon the request of the editor to assess whether the authors have addressed the comments from referee 2, I would like to say the concerns of referee2 are not fully addressed, and the authors need to pay more attention to try to reassure the readers

Details are below:

1. For the reply to question 1, 220 metabolites were investigated based on KEGG, but only 101 metabolite-related genes were surveyed, why only 101 were chosen, is it because these metabolites have more relationship with pear fruit development or maturity? For any other reasons, this should be clarified.

2. For question 2, the authors have done the WGBS as requested. However, as we know, DNA methylation has no direct impact on gene transcription from the level of the whole genome, for specific genes, we could say cytosine methylation regulates genes positively or negatively. Hence, I guess the 948 differentially expressed genes are not all with significant DNA methylation differences. This needs to be clarified to avoid cognitive confusion.

And since 5-Aza treatment would decrease the whole genome-wide DNA methylation level, are the 948 genes also the DEGs between young and mature pear tissues? If these genes have much overlapping with the DEGs during the pear fruit development, then it would give more confidence to let people believe that the DEGs are caused by DNA methylation dynamics. For instance, if one gene is up-regulated during 5-Aza treatment, then this gene should have higher transcription in the young stage compared to the later stages. Statistics are needed.

3. For question 3, I generally agree with the answer of the authors, the decrease of small RNA abundance during pear fruit development didn't support DNA methylation increasing caused by RdDM pathway strengthening. So the DNG/ROS highly expressed in the younger stage would be a perfect explanation. And the same time, I noticed pear fruit would transformed via Agrobacterium. So why not just repress ROS expression in the early stage via this system, and then detect the genome-wide DNA methylation change, as well as detect whether the ROS knock-down would early-mature the pear or not? It would be direct evidence to say ROS expression pattern is the key reason for the DNA dynamics.

4. For questions 5 and 6, I think the authors have shown sufficient evidence to show the pbZFP1 function.

5. I am curious about the 5'-aza solvent, is it DMSO or water? If it is DMSO another solvent, fruit and molecular phenotype in the Fig. 5 deserve suspicion. Please make it clear in your manuscript.

**Authors Response**

**Point-by-point responses to the reviewers’ comments:**

**Reviewer #1:**

The manuscript has been much improved, but the concerns were not fully illustrated. Please see the attached comments.

**1.** The authors conducted the work using fruit flesh and did not consider the fruit pericarp, which is a lack in this manuscript.

*Response: Given that the flavor of pear fruit is predominantly influenced by its flesh, our research focuses primarily on studying fruit flesh development. This line of investigation is crucial in devising effective strategies to enhance the internal quality of pear fruit. Furthermore, it is important to note that the development of the fruit pericarp differs from that of the fruit flesh. If the pericarp and flesh are combined, the resulting data would not accurately reflect the processes specific to either the flesh or the pericarp.*

*Thanks for your suggestion. The development of the fruit pericarp plays a significant role* *in determining the appearance quality of pear fruit. Given that the pericarp comes into direct contact with the external environment, we inferred that DNA methylation plays a critical role in the developmental process of the pericarp. In the future, we plan to conduct a comprehensive multi-omics study to investigate the regulatory mechanisms by which DNA methylation influences pear pericarp development.*

**2.** Figure 1 and Figure 2, simply show the variation/expression trends of metabolites, genes, and proteins is not a good way to elucidate the data. It makes the readers bored and thinks the article was prepared poorly. The trends data mostly should be exhibited as supporting information. Emphasize the key point of the manuscript, not to let the large data swallow the readers.

*Response: Thanks for your suggestion. The visual representations depicting the trends of metabolites, genes, and proteins have been relocated to the supporting information section. Please see details in the revised Figure 1 and 2.*

**3.** Additional file 1, please fulfill the detailed information of all the metabolites, and make it easier for other researchers to follow. The Nature Methods paper can be referred to.

*Response: Thanks for your suggestion. We have added the detailed information of all the metabolites. Please see details in Additional file 1: Table S3.*

4. The profiled protein and small RNA data were not closely related to the manuscript, two aspects need to be considered. Is it due to the un-robust data analysis that the relationship has not been fully understood? Is it still necessary to keep these data? Such big data and not fully analyzed causes a huge amount of redundant information.

*Response: Thanks for your suggestion. The divergence between protein and mRNA expression patterns is widely recognized. Consequently, relying solely on correlation analysis between mRNAs and metabolites when constructing the gene-metabolite database may result in an unreliable outcome. However, by incorporating correlation analysis between proteins and metabolites, the reliability of the gene-metabolite database can be enhanced. Therefore, it is advisable to include protein-metabolite correlation data in this study. The expression levels* *of all identified proteins were presented in Table S21, which is available for gene regulation of fruit metabolites*.

*The involvement of 24-nt siRNA in the RdDM pathway is well-established. To provide an explanation for the observed increase in DNA methylation levels during pear fruit development, it is imperative to elucidate the enrichment of 24-nt siRNA in all developing fruits. Consequently, it is recommended to include these data in this study.*

*In conclusion, although the protein and small RNA data were not fully analyzed in this study, these data would substantiate the investigation of the molecular regulatory network underlying pear fruit flesh development. Therefore, in my opinion, it is crucial to include these data in this study.*

**Reviewer #3:** Upon the request of the editor to assess whether the authors have addressed the comments from referee 2, I would like to say the concerns of referee2 are not fully addressed, and the authors need to pay more attention to try to reassure the readers

*Response: Thanks for your suggestion. We have made the revisions according to your comments.*

Details are below:

1. For the reply to question 1, 220 metabolites were investigated based on KEGG, but only 101 metabolite-related genes were surveyed, why only 101 were chosen, is it because these metabolites have more relationship with pear fruit development or maturity? For any other reasons, this should be clarified.

*Response: We are very sorry for this trouble. The following steps were undertaken to obtain the presented results:*

*(1) A comprehensive search was conducted in the Kyoto Encyclopedia of Genes and Genomes (KEGG) database to identify all 439 metabolites listed in the gene-metabolite database. This search led to the detection of a total of 220 metabolites.*

*(2) However, not all pathway genes associated with the 220 metabolites were available in the KEGG database. Only pathway genes for 136 metabolites could be identified.*

*(3) Among the 136 metabolites with identified pathway genes, transcriptome data analysis enabled the survey of pathway genes for 101 metabolites.*

*(4) Correlation analysis showed that of the 101 metabolites, 63 positively or negatively correlated with their pathway genes, based on the threshold of Pearson coefficient > 0.85 or < -0.85 and false discovery rate < 0.05. The number of metabolites correlated with DEGs are relative. If the threshold was designated as Pearson coefficient >0.80 or < -0.80 and false discovery rate < 0.05, 71 metabolites were correlated to their pathway genes. If the threshold was designated as Pearson coefficient > 0.75 or < -0.75 and false discovery rate < 0.05, 77 metabolites were correlated to their pathway genes. If the threshold was designated as Pearson coefficient > 0.70 or < -0.70 and false discovery rate < 0.05, 82 metabolites were correlated to their pathway genes.*

2. For question 2, the authors have done the WGBS as requested. However, as we know, DNA methylation has no direct impact on gene transcription from the level of the whole genome, for specific genes, we could say cytosine methylation regulates genes positively or negatively. Hence, I guess the 948 differentially expressed genes are not all with significant DNA methylation differences. This needs to be clarified to avoid cognitive confusion.

*Response: Thanks for your suggestion. To address the challenge of low mapping of sequenced reads to the reference genome of pear cultivar Dangshansuli (Pyrus bretschneideri), the genome of pear cultivar Bartlett DH (Pyrus communis) was employed in order to enhance the mapping rate for DNA methylation and transcriptome analyses of fruit callus. As a result, a total of 1426 DEGs were identified in the 5’-Aza-treanted and control calli. Moreover, it is worth noting that DMRs were detected in 1328 of the 1426 DEGs. Please see details in Table S13. These results have been incorporated into the main text. Please see details in page 10 lines 44-50.*

3. And since 5-Aza treatment would decrease the whole genome-wide DNA methylation level, are the 948 genes also the DEGs between young and mature pear tissues? If these genes have much overlapping with the DEGs during the pear fruit development, then it would give more confidence to let people believe that the DEGs are caused by DNA methylation dynamics. For instance, if one gene is up-regulated during 5-Aza treatment, then this gene should have higher transcription in the young stage compared to the later stages. Statistics are needed.

*Response: Thanks for your suggestion. We have conducted a comprehensive analysis of the 1328 DEGs observed between the 5’-Aza-treated and control calli, and found that over half (705 DEGs) of these DEGs were present in the gene-metabolite database. Remarkably, out of the 705 DEGs, 647 exhibited modifications due to DNA methylation during fruit development.*

*It is important to note that DNA methylation is not the sole determinant influencing gene expression during fruit development. Other factors, including environment condition and phytohormones, also possess the potential to exert influence on gene expression throughout the fruit development process. Therefore, it should be understood that the alteration in gene expression may not solely reflect the influence of DNA methylation.*

*Considering that the number of genes expressed at the fruitlet (S1) was smaller compared to later stages (from S3 to S11), we selected the genes repressed by the 5’-Aza treatment in fruit callus to determine if they are up-regulated in the developing fruits at later stages, which exhibit higher levels of DNA methylation compared to the fruitlet. Unexpectedly, after excluding the genes that were not differentially expressed between S1 and other stages, we found that 56.67% to 78.17% of genes were higher expressed in the developing fruits at later stages (from S3 to S11) compared to S1. Please refer to Figure 3f for more details. Although this result further supports the conclusion that the DEGs are caused by DNA methylation dynamics, it remains uncertain whether this outcome is an inevitable or occasional, given the complexity of gene expression regulation.*

4. For question 3, I generally agree with the answer of the authors, the decrease of small RNA abundance during pear fruit development didn't support DNA methylation increasing caused by RdDM pathway strengthening. So the DNG/ROS highly expressed in the younger stage would be a perfect explanation. And the same time, I noticed pear fruit would transformed via Agrobacterium. So why not just repress ROS expression in the early stage via this system, and then detect the genome-wide DNA methylation change, as well as detect whether the ROS knock-down would early-mature the pear or not? It would be direct evidence to say ROS expression pattern is the key reason for the DNA dynamics.

*Response: Thanks for your suggestion. We also hope to validate the participation of ROS in the process of fruit development and ripening through pear transformation. However, it is* *important to note that pear, being a perennial woody fruit tree, undergoes a lengthy juvenile period of at least five years from seedling to flowering. If everything is going well, we will get the transformed plant at the next year. The transformed plant will flower and fruit at the sixth year. It is evident that while pear transformation can be accomplished in few cultivars, the novelty and advancement of the study are compromised when the transformed plant reaches the flowering and fruiting stage. This inherent contradiction presents significant challenges that are difficult to overcome.*

5. For questions 5 and 6, I think the authors have shown sufficient evidence to show the pbZFP1 function.

*Response: Thank you for your affirmation.*

6. I am curious about the 5'-aza solvent, is it DMSO or water? If it is DMSO another solvent, fruit and molecular phenotype in the Fig. 5 deserve suspicion. Please make it clear in your manuscript.

*Response: Thanks for your suggestion. The 5'-Aza compound was dissolved in water. Please see details in page 14 lines 5 and 15 in the last version of this manuscript. This method is consistent with previous studies conducted on orange and strawberry (Cheng et al., 2018; Huang et al., 2019), ensuring consistency in the experimental procedures. We have also added the description in the Methods. Please see details in page 27, line 4 in the revised manuscript.*

*References:*

*Cheng JF, Niu QF, Zhang B, Chen KS, Yang RH, Zhu JK, Zhang YJ, Lang ZB. Downregulation of RdDM during strawberry fruit ripening. Genome Biol. 2018; 19: 212.*

*Huang H, Liu RE, Niu QF, Tang K, Zhang B, Zhang H, Chen KS, Zhu JK, Lang ZB. Global increase in DNA methylation during orange fruit development and ripening. Proc Natl Acad Sci USA. 2019; 116: 1430-1436.*

**Third round of review**

**Reviewer 1**

The manuscript is now suitable for publication.

**Reviewer 3**

The revised manuscript has dispelled all my concerns, I agree to accept this paper and recommend this paper to be published in the Genome biology journal. Shaoli Zhou
